# Origins of unique gold-catalysed chemo- and site-selective C–H functionalization of phenols with diazo compounds†
†Electronic supplementary information (ESI) available: Data for new compounds, experimental procedures and theoretical studies on mechanisms. See DOI: 10.1039/c5sc04319k


**DOI:** 10.1039/c5sc04319k

**Published:** 2015-11-27

**Authors:** Yuan Liu, Zhunzhun Yu, John Zenghui Zhang, Lu Liu, Fei Xia, Junliang Zhang

**Affiliations:** a Shanghai Key Laboratory of Green Chemistry and Chemical Processes , School of Chemistry and Molecular Engineering , East China Normal University , 3663 N. Zhongshan Road , Shanghai 200062 , China . Email: lliu@chem.ecnu.edu.cn ; Email: fxia@chem.ecnu.edu.cn ; Email: jlzhang@chem.ecnu.edu.cn; b State Key Laboratory of Precision Spectroscopy , Institute of Theoretical and Computational Science & NYU-ECNU Center for Computational Chemistry at NYU Shanghai , 3663 Zhongshan Road North , Shanghai 200062 , China

## Abstract

In past decade, gold revealed more and more unique properties in carbene chemistry. It was disclosed in our recent communication (*J. Am. Chem. Soc.* 2014, **136**, 6904) that gold carbenes have unprecedented chemo- and site-selectivity and ligand effect toward the functionalization of C–H bonds in phenols. In this full article, we report a comprehensively combined theoretical and experimental study on the mechanism of the insertion of gold carbenes into C–H and O–H bonds in phenol. It significantly revealed that the ligands have an important effect on C–H insertion and the reaction proceeds through a pathway involving the formation of an enolate-like intermediate. Moreover, two water molecules serving as a proton shuttle are believed to be the key issue for achieving chemoselective C–H functionalization, which is strongly supported by the DFT calculations and control experiments. It is the first time that a clear explanation is given about the prominent catalysis of gold carbenes toward C–H functionalization based on a theoretical and experimental study.

## Abstract 2

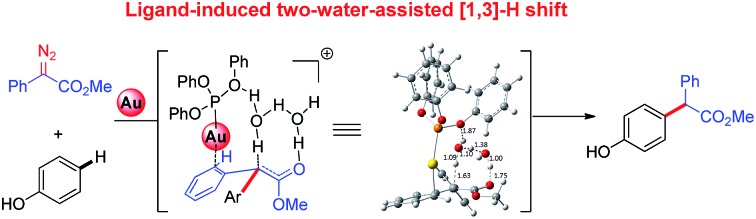
The origins of unique gold-catalyzed C–H functionalization of phenols with diazo compounds were disclosed by combined computational and experimental studies.

## Introduction

Phenols are not only frequently found in natural products and pharmaceutics, but are also powerful versatile platforms in organic synthesis.^1^ Thus, the catalytic functionalization of phenols in a chemo- and site-selective manner is the most encompassing issue in organic chemistry.^2^ In this context, the transition-metal-promoted decomposition of diazo compounds to generate metal carbenes has broad applications^3^ in the direct site-selective functionalization of phenols. In most cases, O–H insertion takes place catalysed by a series of transition-metal catalysts such as copper, palladium, iron, and rhodium complexes.^4^ In contrast, very recently, we and Shi independently found the unprecedented C(sp^2^)–H bond functionalization of phenols with α-diazoesters,^5^ furnishing *para*-substituted phenols **Pro-c8***via para* C–H functionalization rather than the O–H insertion product **Pro-o8**. Meanwhile, the chemoselectivity is heavily dependent on the nature of the ligand of the gold catalyst^6^ (Table 1). Although Nakamura and Pérez *etc.*^7^ have theoretically studied the mechanism of C(sp^3^)–H insertion with carbenes, and Shi and co-workers5*b* analysed the electronic structures of gold carbenes by using Density Functional Theory (DFT) calculations, no comprehensive mechanistic insight on the overall pathways of aromatic C(sp^2^)–H insertion with carbenes has been provided so far. To further understand the origin of this unique behaviour of gold carbenes^8^ in site selective C–H functionalization of phenol, we decided to carry out DFT calculations and an experimental study to gain insights on the mechanism. Herein, we present a combined computational and experimental study to elucidate the origin of the ligand effect on the chemoselectivity. Furthermore, the trace amount of water in the reaction mixture serving as a proton shuttle is proposed to be the key to give rise to the product **Pro-c8** of C–H insertion, which is supported by our current DFT calculations and control experiments.

**Table 1:** C(sp^2^)–H bond functionalization of phenols with α-diazoesters under various conditions

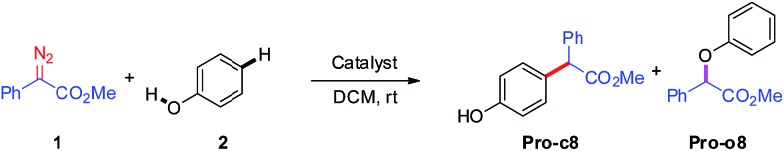				
Entry	Catalyst (5 mol%)	Time	**Pro-c8** ^*a*^	**Pro-o8** ^*a*^
1^*b*^	Cu(OTf)_2_	5 min	0	11
2^*b*^	Cu(OTf)·toluene	5 min	0	9
3^*b*^	[Pd(CH_3_CN)_2_Cl_2_]	12 h	0	33
4	Fe(OTf)_2_	12 h	NR	
5^*c*^	FeCl_2_	12 h	0	0
6^*b*^	[Rh_2_(OAc)_4_]	5 min	0	36
7	Ph_3_PAuSbF_6_	5 min	33	45
8	(PhO)_3_PAuSbF_6_	5 min	82	0

^*a*^NMR yield.

^*b*^The conversion of **1** is 100% and the major product is dimer of **1**.

^*c*^Trace amount of dimer from **1** was detected.

In a previous work on rhodium catalysed intramolecular aromatic C(sp^2^)–H insertion reactions, Padwa and Doyle assumed that the reactions were triggered by the electrophilic addition of the metallocarbene intermediate to the aromatic ring, then followed by a [1,2]-H migration step.^9^ Nevertheless, the reaction mechanisms for the prominent activity of gold carbenes toward C–H bonds still remain elusive. On the other hand, despite Yu and coworkers^10^ having performed the mechanistic study of the copper- and rhodium-catalysed O–H insertion of water with carbene using DFT calculations, the mechanism of gold-catalyzed O–H insertion has not yet been explored. In principle, there are three pivotal questions unsolved in this transformation: (1) is the mechanism of C(sp^2^)–H insertion with gold carbenes the same as that with other metals such as rhodium? (2) What is the origin of the ligand effect on the chemoselectivity? (3) Besides the ligand, any other factors affect the chemoselectivity?

## Computational methods

All of the DFT calculations were carried out using the Gaussian 09 software package.^11^ The geometric structure of the intermediates and transition states were optimized and located by using the M06 functional,^12^ since the M06 functional has been demonstrated to generate accurate results for organometallics especially in the description of noncovalent interactions. The Lanl2dz basis set^13^ combined with the relativistic effective core potential for inner electrons and double zeta basis for covalent electrons was used to describe the heavy elements Au and P, and the 6-31G* basis set^14^ was used to describe the nonmetallic elements C, N, O and H. Frequency analyses were also performed based on the structures obtained in the gas phase to confirm that the intermediates are local minima and the transition states have only one imaginary frequency. Intrinsic reaction coordinate (IRC) calculations^15^ were performed to make sure that the crucial transition states connect the correct reactants and products. The solvent effect of CH_2_Cl_2_ was evaluated with single point calculations using the integral equation formalism model (IEFPCM)^16^ with the dielectric constant *ε* = 8.93 based on the structures in the gas phase. All discussed energy values of intermediates and transition states throughout this paper are the Gibbs free energies in units of kcal mol^–1^ that were calculated at the temperature of 298 K, including the corrections of solvation free energies from the IEFPCM model. The 3D images of the calculated structures were plotted using GaussView.^17^ More structural details of intermediates and transition states can be found in the ESI.†

## Results and discussion

### On the pathway of C–H functionalization catalysed by (PhO)_3_PAuSbF_6_

It has been widely accepted that metal catalysts could catalyse diazo compounds to release N_2_ and form reactive Au-carbenes, which were regarded as the precursors responsible for C–H bond insertion.^5^,8a–e To account for the generation of crucial precursors preceding C–H insertion, we calculated the reaction pathways of α-diazoesters and metal catalysts AuL (L = (PhO)_3_P and Ph_3_P) in solution (Fig. S1 of ESI†). The calculated free energy profiles indicate that the generation of Au-carbene species is quite facile and drastically exothermic, with the low barriers of 6.5 and 11.4 kcal mol^–1^, respectively. Therefore, the gold carbenes and phenol are regarded as the reactants for the subsequent C–H bond insertion reactions and the sum of their free energies is set as the benchmark, with the value of 0.0 kcal mol^–1^.

**Fig. 1:**
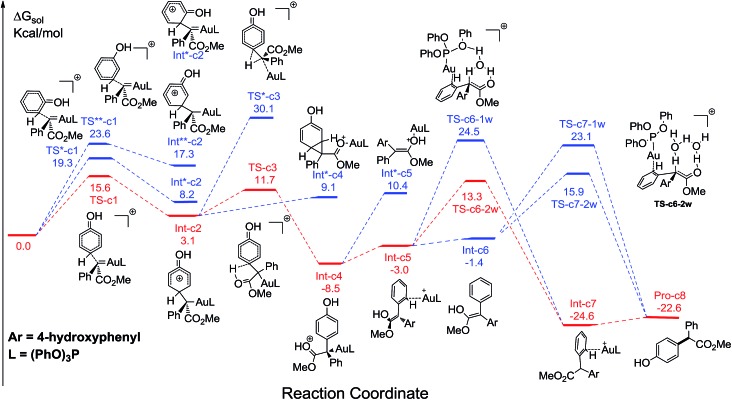
The calculated free energy profiles Δ*G* and the corresponding structures of intermediates and transition states along different possible C–H insertion pathways catalyzed by (PhO)_3_PAuSbF_6_. The reasonable pathways are shown in red and other possible pathways are in blue. All values of free energies are relative to the gold carbene and phenol reactants and in the units of kcal mol^–1^.

**Fig. 2:**
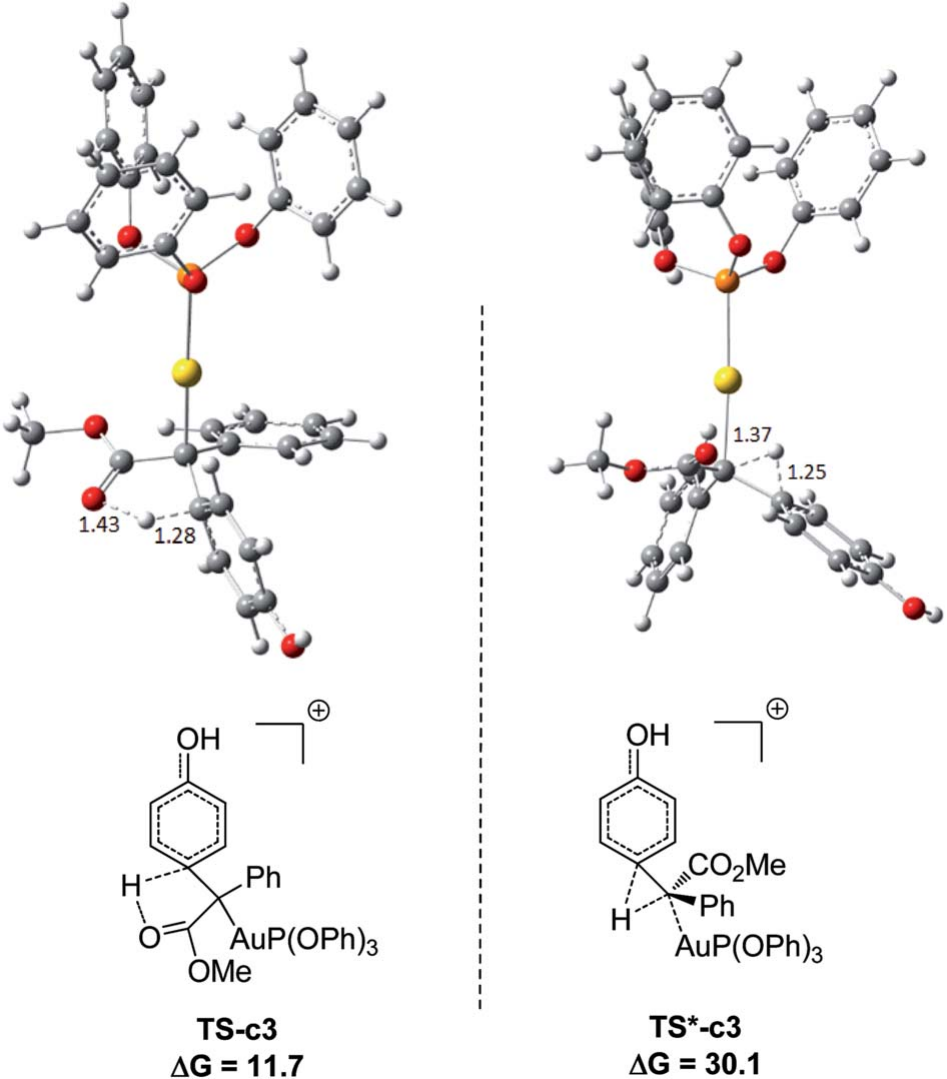
Optimized structures of transition states **TS-c3** and TS*-c3 corresponding to Fig. 1. The distances are in the units of angstroms and the values of free energies Δ*G* are in kcal mol^–1^.

Fig. 1 displays the calculated free energy profiles for the C(sp^2^)–H functionalization of phenol with a gold carbene with (PhO)_3_P as the ligand, where the structural details of intermediates and transition states are provided in the ESI.† The previous theoretical investigations on the C(sp^3^)–H insertion of rhodium, copper or silver carbenes, performed by Nakamura and Pérez, indicate that the first step of the reactions involves the formation of σ-bond complexes of carbenes and the C(sp^3^)–H bond.^7^ Recently, Pérez and coworkers suggested that the mechanism of aromatic C(sp^2^)–H bond insertion might be entirely different from that of aliphatic C(sp^3^)–H bond insertion.8*c* In our DFT calculations, a transition state **TS-c1** with a barrier of 15.6 kcal mol^–1^ is located for the addition of the gold carbene at the *para*-position of phenol. **TS-c1** represents the transition state of the direct C(sp^2^)–C(sp^2^) coupling of phenol and the gold carbene, leading to the final *para*-C–H insertion product **Int-c2**. Accordingly, the addition of the carbene at the *ortho* site of phenol gives rise to an intermediate Int*-c2 with a higher energy (8.2 kcal mol^–1^*vs.***Int-c2**'s 3.1 kcal mol^–1^) *via* the transition state TS*-c1 with 19.3 kcal mol^–1^. It is not surprising to find that the *meta*-position of phenol is the most unfavourable site for addition with the gold carbene, which needs an activation energy barrier up to 23.6 kcal mol^–1^. Such high activation barriers of TS*-c1 and TS**-c1 prevent the carbenes from adding at the *ortho*- and *meta*-positions of phenol. The current DFT results are consistent with our experimental observations that the addition at the *para*-sites of substituted phenols was the predominant even with bulky steric hindrance (*i.e.* 3,5-dimethyl phenol).5*a* According to the previous calculations,^10^ the intermediate **Int-c2** may be isomerized to the enolate *via* the mechanism of [1,3]-metal migration and yield a stable intermediate. However, it is found that such a stable intermediate hardly exists after the [1,3]-Au migration in **Int-c2**, since a kind of cyclopropanation product of the Büchner reaction is obtained, as shown by Int*-c4.^18^ Meanwhile, this process of [1,3]-Au migration seems to be energetically unfavorable, with the endothermicity of 9.1 kcal mol^–1^ to give Int*-c4. Additionally, in order to examine whether the commonly accepted mechanism of direct [1,2]-H shift is feasible, we located the transition state TS*-c3 with the corresponding structure displayed in Fig. 2. Our calculated results imply that TS*-c3 has a considerable barrier of 30.1 kcal mol^–1^, which could definitely rule out the mechanism of direct [1,2]-H shift in **Int-c2**. During the course of optimization, we happened to hypothesize that the carbonyl oxygen atom may intend to directly abstract the proton at the original *para*-site of phenol *via* the five-membered transition state **TS-c3** in Fig. 2, to generate **Int-c4**. This process is quite facile according to the current calculations, merely with a small barrier of 8.6 kcal mol^–1^. Also, **Int-c4** is more stable than Int*-c4 by 17.6 kcal mol^–1^. Subsequently, the intermediate **Int-c4** undergoes an unexpected isomerization to the enol **Int-c5** through [1,3]-migration of the metal complex (PhO)_3_PAu^+^ to the phenyl ring rather than to Int*-c5.

**Fig. 3:**
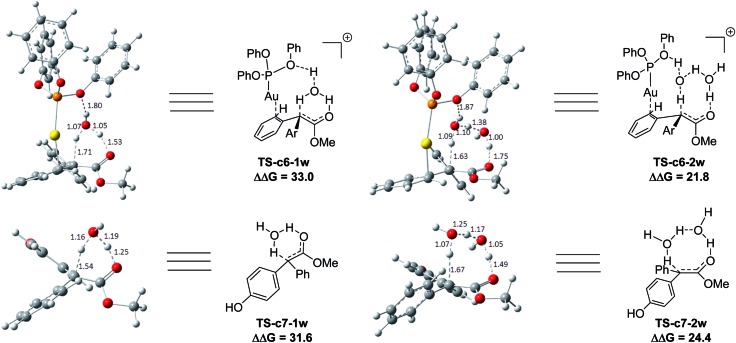
The structures of **TS-c6-1w**, **TS-c6-2w**, **TS-c7-1w** and **TS-c7-2w** in the pathways of C–H functionalization of phenol catalysed by (PhO)_3_PAuSbF_6_. The values of the free energy barriers ΔΔ*G* are in kcal mol^–1^.

Two possible pathways might exist between the enol **Int-c5** and the final product **Pro-c8**. One is the gold-associated pathway, where the proton transfers through **TS-c6-1w** or **TS-c6-2w** to yield the intermediate **Int-c7**. Then, the metal complex (PhO)_3_PAu^+^ dissociates from **Int-c7** to yield **Pro-c8**. The other is the gold-free pathway, where the catalyst (PhO)_3_PAu^+^ first dissociates from **Int-c5** to yield the gold-free **Int-c6** and then proton transfer occurs *via***TS-c7-1w** or **TS-c7-2w** to yield **Pro-c8**. As far as the two pathways are concerned, the key question is: how does the proton transfer from the hydroxyl to the final carbon in the enol? In this work, we propose that the water in the solvent plays a crucial role in undertaking the remote proton shuttle. Based on this proposal, we located the one-water assisted transition states **TS-c6-1w** and **TS-c7-1w**, as well as the two-water assisted transition states **TS-c6-2w** and **TS-c7-2w** along the reaction pathways, with the corresponding activation energy barriers of the elementary steps displayed in Fig. 3. The DFT results indicate that **TS-c6-1w** and **TS-c7-1w** involving one water molecule has higher energy barriers than two-water assisted **TS-c6-2w** and **TS-c7-2w**. Fig. 3 shows that the carboxyl group in **TS-c6-1w** and **TS-c7-1w** has to distort from the planar structures to form hydrogen bonds with H_2_O, but they still remain planar in **TS-c6-2w** and **TS-c7-2w**. The structural distortion in **TS-c6-1w** and **TS-c7-1w** might be the reason why they are in high energy states. Interestingly, it is found that the oxygen atoms in the ligand (PhO)_3_P form hydrogen bonding interactions with the shuttle water molecules in **TS-c6-2w**, while this effect does not exist in the other located transition state TS*-c6-2w (Fig. S2 in ESI†). The calculated distance between the oxygen and hydrogen atoms in **TS-c6-2w** of Fig. 3 is 1.87 Å and the energy of the transition state is 2.2 kcal mol^–1^ lower than that of TS*-c6-2w, indicating a possible stabilization effect of the metal complex (PhO)_3_PAu^+^ for the water chain in **TS-c6-2w**.

**Scheme 1:**
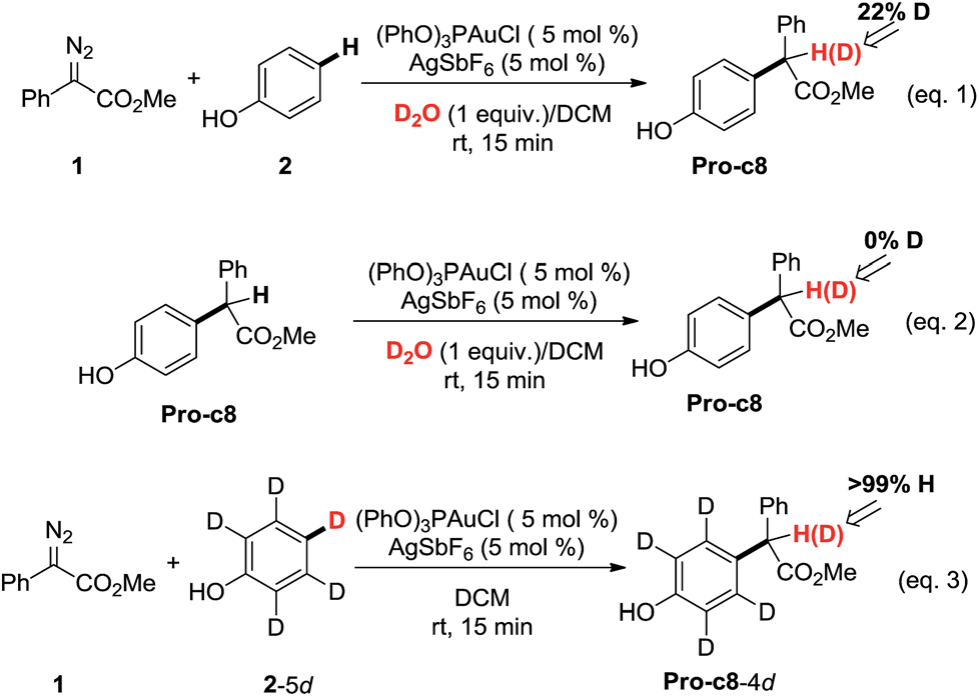
Control experiments.

Control experiments were carried out in our lab (Scheme 1) to demonstrate the proposed mechanisms of gold carbene insertion into the C–H bond of phenol. When 1 equivalent of D_2_O was added into the reaction system, product **Pro-o8** was deuterated with the percentage of 22% (eqn (1)). The relatively low deuterium ratio might be attributed to the existence of trace amount of H_2_O^19^ that could also undertake the function of proton transfer. Additionally, it is well known that the hydrogen in the OH group of phenol could readily exchange with the deuterium in D_2_O, which leads to an increased H_2_O or DOH amount in solution. If a great number of H_2_O molecules participate in the process of the proton shuttle, the ratio of deuterated product **Pro-o8** will definitely decrease. We also considered the possibility whether the phenols can serve as the proton shuttle. However, the calculated energy barrier between the transition state structure **TS-c6-2p** (ESI†) and **Int-c5** for [1,3]-H shift is highly up to 36.1 kcal mol^–1^, which implies that it hardly occurs. Eqn (2) manifests that the direct proton exchange of **Pro-o8** with D_2_O is impossible under the reaction conditions. More importantly, eqn (3) demonstrates that the reaction of **1** and deuterated phenol **2**-5d gives rise to the product **Pro-o8** without deuterium, which supports that direct [1,2]-H shift^9^ (TS*-c3) does indeed not occur in this case. The control experiments demonstrate that this kind of C–H bond insertion reactions governed by a gold carbene follows a novel mechanism involving a water assisting process, rather than previous commonly proposed direct [1,2]-H shift.^5^,8a,8c,^9^

### Proposed pathway of O–H insertion catalysed by (PhO)_3_PAuSbF_6_

**Fig. 4:**
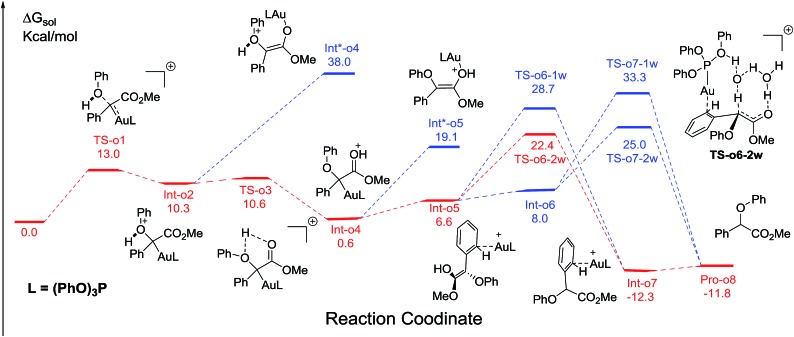
The calculated free energy profiles Δ*G* and the corresponding structures of the intermediates and transition states along different possible O–H insertion pathways catalyzed by (PhO)_3_PAuSbF_6_. The reasonable pathway is shown in red and other possible pathways are in blue. All values of free energies are relative to the gold carbene and phenol reactants and in the units of kcal mol^–1^.

**Fig. 5:**
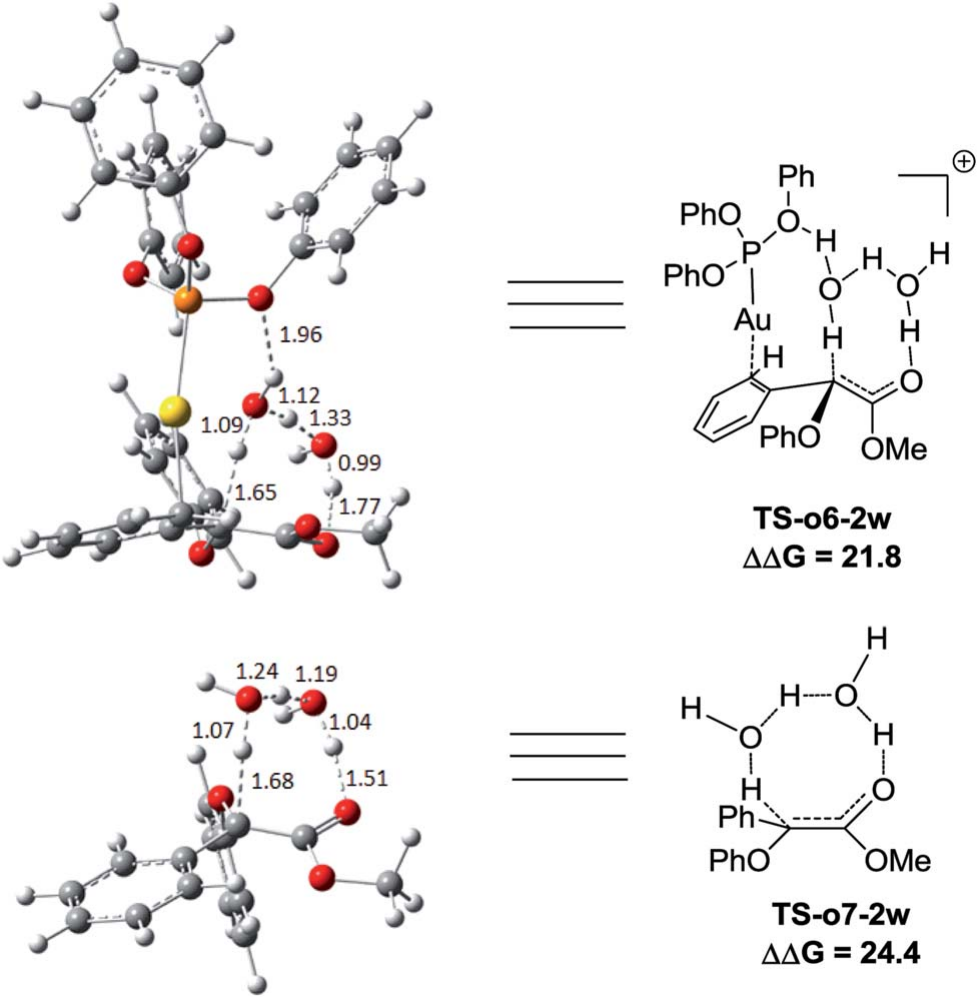
The structures of **TS-o6-2w** and **TS-o7-2w** in the proposed O–H functionalization of phenols catalysed by (PhO)_3_PAuSbF_6_. The distances are in the units of angstroms and the values of free energy barriers ΔΔ*G* are in kcal mol^–1^.

We next turn to address the question why the O–H insertion of phenol does not occur with (PhO)_3_PAuSbF_6_ as the catalyst. In Fig. 4, the calculated results show that oxonium ylide^20^**Int-o2** formed through **TS-o1** with a modest barrier. In the case of O–H bond insertion, a possible migration of complex LAu^+^ to the carbonyl group in **Int-o2** is also taken into account, but the resulted structure Int*-o4 is quite high in energy. The oxonium ylide **Int-o2** tends to isomerize to the more stable **Int-o4***via***TS-o3**. The migration of the gold catalyst to the phenyl group in **Int-o4** leads to the isomer **Int-o5**. It should be noted that Int*-o5 is quite energetically unstable, with the gold complex attached to the oxygen atom in the hydroxyl group. Similarly to the mechanism of C–H insertion in Fig. 1, the rearrangements of **Int-o5** to **Pro-o8** pass through the gold-associated or gold-free pathways *via* the specific water-assisted transition states. We also locate the two-water assisted transition states **TS-o6-2w** for the gold-associated pathway and **TS-o7-2w** for the gold-free pathway, respectively (Fig. 5). The activation barrier of **TS-o6-2w** (15.8 kcal mol^–1^) is relatively lower that of **TS-o7-2w** (18.4 kcal mol^–1^), indicating that the transformation of **Int-o5** to **Pro-o8** favours the gold-associated pathway *via***TS-o6-2w** rather than **TS-o7-2w**.

### Rationale of the chemoselectivity

Comparing these two competitive reaction pathways in Fig. 1 and 4, it is revealed that both rearrangements from **Int-c5** or **Int-o5** to the final products are the rate-determining steps in the overall pathways, with almost the same total barriers of 21.8 kcal mol^–1^. Nevertheless, the DFT calculations reveal that the thermal stability of the intermediate of C–H insertion remarkably differs from that of O–H insertion. For example, the intermediate **Int-c4** is more stable than **Int-o4** by 9.1 kcal mol^–1^, with an exothermic energy of 8.5 kcal mol^–1^. As for the final products, the energy of **Pro-c8** is 10.8 kcal mol^–1^ lower than that of the O–H insertion product **Pro-o8**. Besides, the endothermicity of **TS-c6-2w** is 13.3 kcal mol^–1^, whereas the corresponding energy of **TS-o6-2w** is up to 22.4 kcal mol^–1^, relative to the sum of the reactants. Such a large endothermicity of **TS-o6-2w** means that it is more difficult to pass through it to reach the product though collision in solution. The calculated reaction pathways in Fig. 1 and 4 reveal that the stability of intermediates plays an important role in chemoselectivity and accounts for the unique chemoselectivity toward C(sp^2^)–H insertion.

**Fig. 6:**
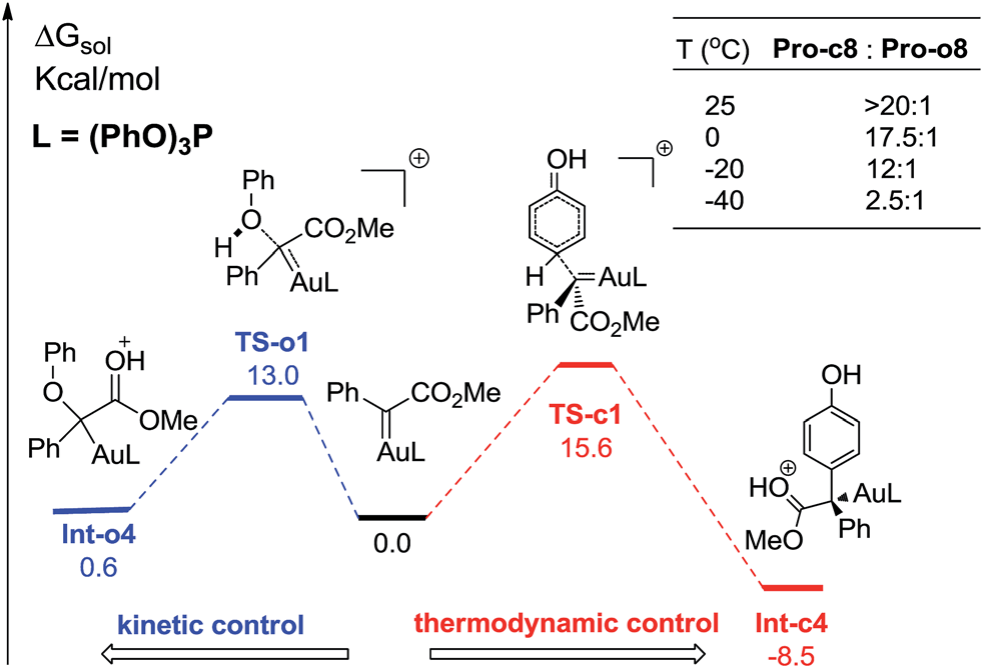
The reaction temperature on the chemoselectivity and the competitive pathways for the formation of two key intermediates. All values of free energies are relative to the gold carbene and phenol reactants and in the units of kcal mol^–1^.

In order to further understand the chemoselectivity, the reactions of phenol with α-phenyldiazoester catalysed by (PhO)_3_PAuSbF_6_ at different temperatures were also carried out. The ratios of **Pro-c8** : **Pro-o8** decreased from >20 : 1 to 2.5 : 1 along with lowering the reaction temperature from rt to –40 °C (Fig. 6; for details, see ESI†). These results indicated that the C–H functionalization process was more favored at high temperature than at low temperature. According to the free energy showed in Fig. 1 and 4, **Int-c4** and **Int-o4** should be the key intermediates for C–H functionalization and O–H insertion. As shown in Fig. 6, **Int-c4** was more stable *via* a higher barrier whilst **Int-o4** was more unstable *via* a lower barrier. That is, the formation of **Int-c4** was the process of thermodynamic control, leading to C–H functionalization. In contrast, the generation of **Int-o4** underwent kinetic control, leading to O–H insertion. The control experiments at different temperatures and DFT calculations are in excellent agreement.

### Two competitive pathways catalyzed by Ph_3_PAuSbF_6_

**Fig. 7:**
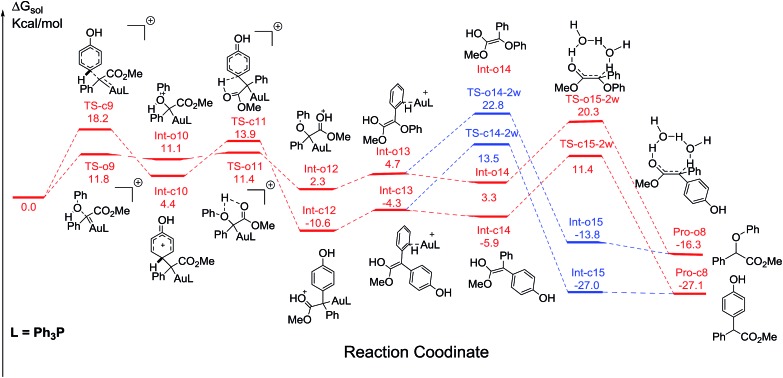
The competitive pathways of C–H and O–H insertion catalyzed by Ph_3_PAuSbF_6_. The two reasonable pathways are shown in red and the other possible pathways in blue. All values of free energies are relative to the gold carbene and phenol reactants and in the units of kcal mol^–1^.

Additionally, the mechanisms of C–H and O–H insertions of phenol catalyzed by Ph_3_PAuSbF_6_ are also shown in Fig. 7, with the corresponding barriers listed in Table 2. On the one hand, compared to the corresponding pathways in Fig. 1 and 4, we found that the ligand Ph_3_P has a remarkable electronic effect on the change of dynamics barriers of electrophilic addition to intermediates **Int-o12** and **Int-c12**. For C–H insertion, the addition barrier *via***TS-c9** was enhanced to be 18.2 kcal mol^–1^, 2.6 kcal mol^–1^ higher than that of **TS-c1**. In contrast, the barrier of O–H insertion *via***TS-o9** decreases from 13.0 to 11.8 kcal mol^–1^. It is no doubt that the change of the barriers of addition is disadvantageous to C–H insertion but advantageous to O–H insertion. On the other hand, the calculated barrier of hydrogen transfer *via***TS-o15-2w** in O–H insertion is 18.0 kcal mol^–1^, relatively lower than the barrier of 22.0 kcal mol^–1^ for C–H insertion. Therefore, the reaction of C–H insertion shown in Fig. 7 is significantly slowed down in comparison to the case when it is catalysed by (PhO)_3_PAuSbF_6_ in Fig. 1. Product of C–H insertion is still more stable than the counterpart of O–H insertion in terms of thermodynamics according to the current DFT calculations. Considering both the dynamic and thermodynamic effects, the calculated pathways (Fig. 7) could account for the low chemoselectivity for **Pro-c8** and **Pro-o8** catalysed by Ph_3_PAuSbF_6_ that has been observed in our experiments (Table 1, entry 7).

**Table 2:** The calculated free energetic spans ΔΔ*G* of hydrogen transfer through transition states (TSs) assisted by one water molecule and two water molecules in Fig. 6. The values of ΔΔ*G* are in the units of kcal mol^–1^

TSs	**TS-c14-2w**	**TS-c15-2w**	**TS-o14-2w**	**TS-o15-2w**
ΔΔ*G*	24.1	22.0	20.5	18.0

## Conclusions

In summary, the origins of the unique chemoselectivity of C–H bond functionalization of phenol with diazo compounds catalyzed by (PhO)_3_PAuSbF_6_ are due to the high thermodynamic stability of intermediates and products, as well as the modest energy barrier of the rate-determining step. It is proposed for the first time that the reactions of gold carbenes with phenol pass through the specific pathways involving the formation of stable enols. The hydrogen transfers in the rearrangements from enol to final products follow a novel reaction pathway with two water molecules serving as a proton shuttle rather than the previous commonly proposed direct [1,2]-H shift. The DFT calculations also reveal that the oxygen atoms in the ligand (PhO)_3_P play an important role in stabilizing the transition states of hydrogen transfer. The observed ligand effects of metal complexes on the reaction pathways are well rationalized based on the results of the present DFT calculations. The novel mechanistic insights delineated above will refine the model of gold catalyst in carbene chemistry and contribute to the future development of this field as well as the rational designing in chemo- and site-selective organic transformation.

## Supplementary Material

Supplementary information
