# Supplementary material for: Origins of unique gold-catalysed chemo- and site-selective C–H functionalization of phenols with diazo compounds
Source: Chem Sci. 2015 Nov 27;7(3):1988–95. doi: 10.1039/c5sc04319k (PMC5968449; doi:10.1039/c5sc04319k)
Supplement: Supplementary file 1 [file SC-007-C5SC04319K-s001.pdf]

Electronic Supplementary Material (ESI) for Chemical Science.
This journal is © The Royal Society of Chemistry 2015

Supporting Information

Origins of Unique Gold-Catalyzed Chemo- and Site-Selective C-H Functionalization of Phenols with Diazo Compounds

Yuan Liu,[ab‡] Zhunzhun Yu,[a‡] John Zenghui Zhang,[ab] Lu Liu,*[a] Fei Xia,*[ab] and Junliang Zhang*[a]

[a] Shanghai Key Laboratory of Green Chemistry and Chemical Processes, School of Chemistry and Molecular Engineering, [b] State Key Laboratory of Precision Spectroscopy, Institute of Theoretical and Computational Science & NYU-ECNU Center for Computational Chemistry at NYU Shanghai, 3663 Zhongshan Road North, Shanghai 200062, China.

Section 1: Variation of metal catalyst -- S 2
Section 2: Control experiments I --S 3
Section 3: Control experiments II --- S 5
Section 4: Control experiments III--- S 7
Section 5: Kinetic studies I --- S 8
Section 6: Kinetic studies II -- S 12
Section 7: Figures S1 and S2--- S 13
Section 8: Computational methods -- S 14
Section 9: Cartesian coordinates of structures in Figure 1 ------------------------------------- S 15
Section 10: Cartesian coordinates of structures in Figure 4 ------------------------------------ S 51
Section 11: Cartesian coordinates of structures in Figure 6 -------------------------------------S 75
Section 12: ^{1}H-NMR spectrum -- S 109

Section 1: Variation of Metal Catalyst.[a]

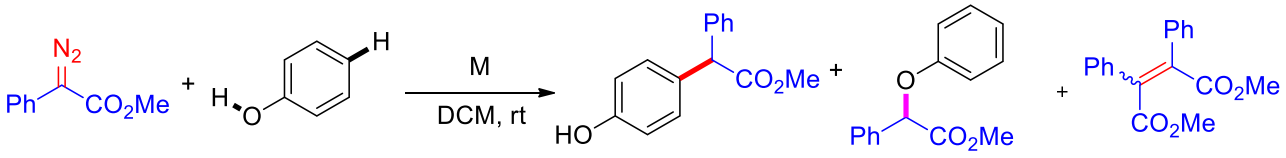

Entry	M	Time[e]	**Pro-c8**[b]	**Pro-o8**[b]	**dimer**[b]
1[c]	$Cu(OTf)_2$	5 min	0	11	28
2[c]	Cu(OTf) toluene	5 min	0	9	10
3[d]	$[Pd(CH_3CN)_2Cl_2]$	12 h	0	33	18
4	$Fe(OTf)_2$	12 h	NR		ND
5[d]	$FeCl_2$	12 h	0	0	trace
6[c]	$[Rh_2(OAc)_4]$	5 min	0	36	32
7[f]	$Ph_3PAuSbF_6$	5 min	33	45	trace
8	$(PhO)_3PAuSbF_6$	5 min	82	0	trace

[a] The reaction was carried out with **1** (0.4 mmol), **2** (0.6 mmol), catalyst (5 mol%) in CH_2Cl_2 (4 mL) at room temperature. [b] NMR yield. [c] Then conversion of **1** is 100% and the major product is dimer of **1**. [d] Trace of dimer was detected. [e] The reaction time means the time after finishing the injection of **1.** [f] Isolated yields.

General procedure for this process:

In a dried glass tube, to a mixture of metal catalyst (5 mol%) and Phenol (56.4 mg, 0.6 mmol) in CH_2Cl_2 (4 mL) was added diazo compound (70 mg, 0.4 mmol) in 1 mL of CH_2Cl_2 by a syringe in a period of 15 min at room temperature. The resulting mixture was continually stirred at room temperature until **1** was consumed completely determined by TLC analysis. The mixture was passed through a short silica gel column and then concentrated under reduced pressure. The yield was determined by ^{1}H-NMR of crude product, using CH_2Br_2 as internal standard

Section 2: Control experiments I.

1. Procedure for eq. 1:

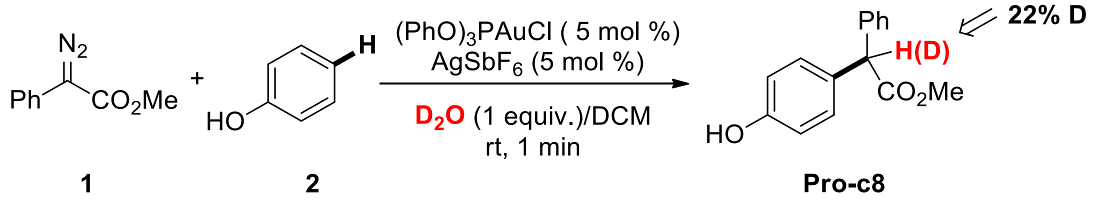

(eq. 1)

In a dried glass tube, to a mixture of gold catalyst $(PhO)_3PAuCl$ (10.8 mg, 0.02 mmol) in CH_2Cl_2 (4 mL) was added $AgSbF_6$ (6.9 mg, 0.02 mmol) and stirred for 15 min at room temperature. Then, phenol (56.4 mg, 0.6 mmol) and D_2O (7.2 μL, 0.4 mmol) were added and also stirred for 15 min. Subsequently, diazo compound **1** (70 mg, 0.4 mmol) in 1 mL of CH_2Cl_2 was added by a syringe in a period of 15 min at room temperature. The resulting mixture was continually stirred at room temperature until **1** was consumed completely determined by TLC analysis. The mixture was passed through a short silica gel column and then concentrated under reduced pressure. The deuterium ratio was determined by ^{1}H-NMR of crude product, using CH_2Br_2 as internal standard.

2. Procedure for eq. 2:

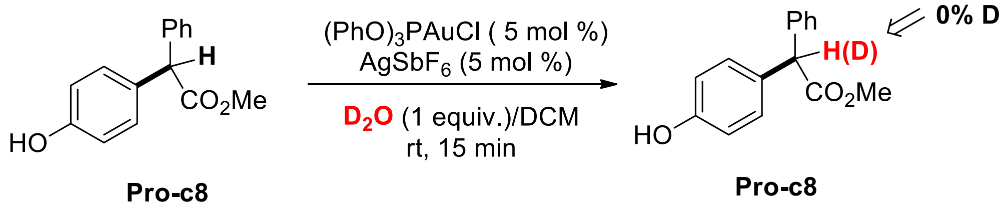

(eq. 2)

In a dried glass tube, to a mixture of gold catalyst $(PhO)_3PAuCl$ (10.8 mg, 0.02 mmol) in CH_2Cl_2 (4 mL) was added $AgSbF_6$ (6.9 mg, 0.02 mmol) and stirred for 15 min at room temperature. Then, compound **3** (96.8 mg, 0.6 mmol) and D_2O (7.2 μL, 0.4 mmol) were added. The reaction was continually stirred for 15 min at room temperature. The mixture was passed through a short silica gel column and then concentrated under reduced pressure. The deuterium ratio was determined by ^{1}H-NMR of crude product, using CH_2Br_2 as internal standard.

3. Procedure for eq. 3:

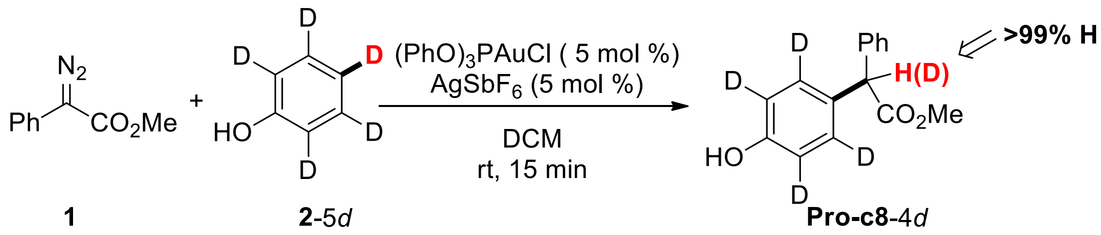

(eq. 3)

In a dried glass tube, to a mixture of gold catalyst $(PhO)_3PAuCl$ (10.8 mg, 0.02 mmol) in CH_2Cl_2 (4 mL) was added $AgSbF_6$ (6.9 mg, 0.02 mmol) and stirred for 15 min at room temperature. Then, phenol **2**-5*d* (59.4 mg, 0.6 mmol) was added and diazo compound **1** (70 mg, 0.4 mmol) in 1 mL of CH_2Cl_2 was added by a syringe in a period of 15 min at room temperature. The resulting mixture was continually stirred at room temperature until **1** was consumed completely determined by TLC analysis. The mixture was passed through a short silica gel column and then concentrated under reduced pressure. The deuterium ratio was determined by ^{1}H-NMR of crude product, using CH_2Br_2 as internal standard.

Section 3: Control experiments II.

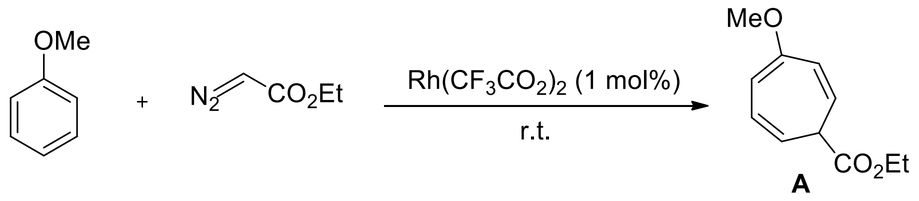

(eq. 4)

To a mixture of $Rh(CF_3CO_2)_2$ (66 mg, 0.1 mmol) in anisole (5 mL) was added ethyl diazoacetate (1.14 g, 10 mmol) over a period of 10 min at room temperature. The reaction was done when the diazo compounds was added completely. The reaction was purified by silica chromatography (PE / EA = 30:1) immediately and desired product **A** was obtained (592 mg, 30%) . ^{1}H NMR (400 MHz, $CDCl_3$) δ 6.21(t, *J* = 8.0 Hz, 1H), 6.07 (d, *J* = 9.6 Hz, 1H), 5.80-5.95 (m, 1H), 5.61 (dd *J* = 10 Hz, 6.0 Hz, 1H), 5.26 (dd *J* = 9.2 Hz, 5.6 Hz, 1H), 4.25 (q, *J* = 7.2 Hz, 2H), 3.66 (s, 3H), 2.69 (t, *J* = 5.6 Hz, 1H), 1.30 (t, *J* = 7.2 Hz, 3H); ^{13}C NMR (100 MHz, $CDCl_3$) δ:14.20, 44.03, 54.68, 61.03, 104.82, 113.29, 119.99, 122.08, 124.77, 159.97, 172.91

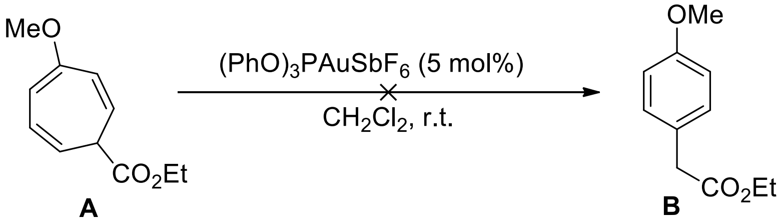

(eq. 5)

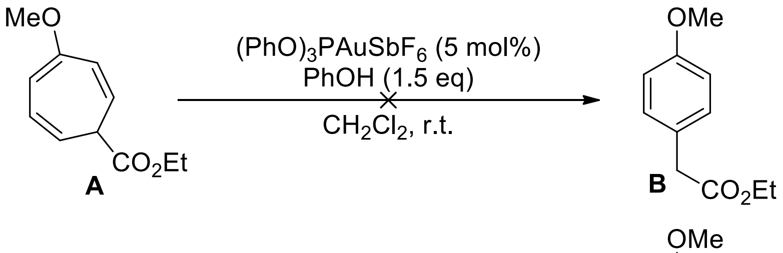

(eq. 6)

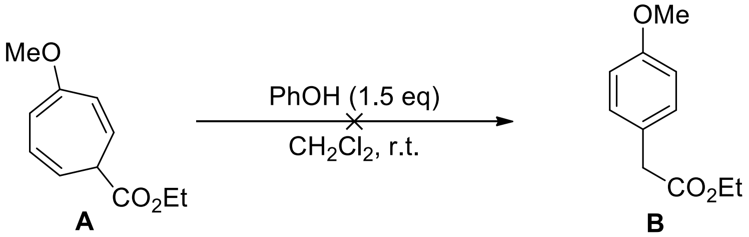

(eq. 7)

In order to rule out the pathway that the C-H functionalization product was formed via rearrangement of the product of Büchner reaction, cycloheptatriene **A** was synthesized (eq. 4) and treated with gold, gold/phenol, phenol respectively (eq. 5-7). No desired rearrangement product **B** was determined in these conditions, indicating that the rearrangement of cycloheptatriene did not occur in our catalytic system.

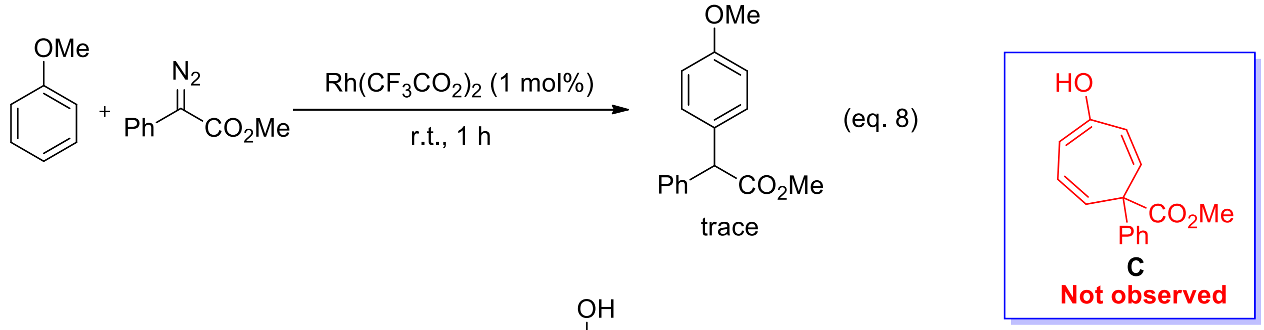

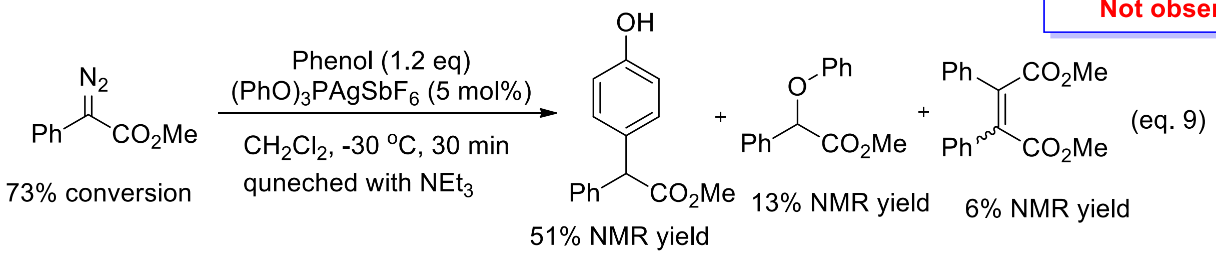

Meanwhile, the reaction of anisole and *α*-phenyl-*α*-diazoester under the condition used in eq. 4 did not give any cycloheptatriene **C** (eq 8). If the reaction was catalyzed by $(PhO)_3PAuSbF_6$ at -50 ^{o}C and quenched by NEt_3 after 30 mins, there are still 27% diazoester left while no cycloheptatriene **C** was detected by crude NMR.

Combined above results and DFT calculations, the pathway involving Büchner reaction might be ruled out.

Section 4: Control experiments III.

Reaction at various temperatures.

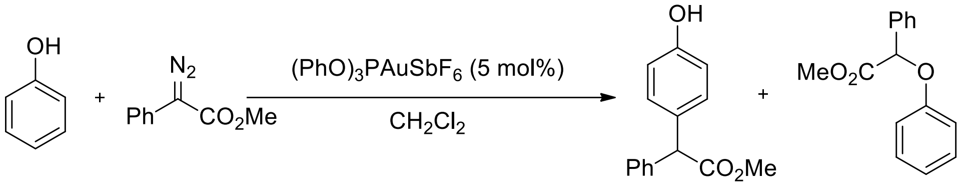

1.5 equiv **Pro-c8** **Pro-o8**

Temperature (°C)	Ratio of **Pro-c8** vs **Pro-o8**
25	> 20:1
0	17.5:1
-20	12:1
-40	2.5:1

Section 5: Kinetic studies I

According to the reference[1], initial rates were used to determine the orders of the individual components for the reaction.

1. Order for phenol

To a dried glass tube was added Phenol solution (0.1 mmol, 0.2 mmol, 0.3 mmol, 0.4 mmol, 0.5 mmol, respectively, 1 M in CH_2Cl_2) and (2, 4-^{t}Bu$C_6H_3O)_3PAuSbF_6$ (0.01 mmol, 0.01 M in CH_2Cl_2), which was preformed prior to be used. Subsequently, CH_2Cl_2 was added, keeping the reaction volume is equal to 3.8 mL. The reaction was cooled to -45 °C and diazo (0.2 mmol, 0.2 mL solution in CH_2Cl_2) was added and stirred at -45 °C for 1h. The reaction was quenched with diluted Et_3N solution in acetone. The yield was determined by GC-MS with dodecane (7.8 mg, 10 μL) as internal standard.

Plot ofΔ[pro-c8]/Δt * 10^4 vs initial concentration of phenol **1**.

**[PhOH]$_0$ (M)**	**Initial Δ[pro-c8]/Δt * 10^4 (M/min)**		
	Run 1	Run 2	Average
**0.025**	1.05	1.60	1.33
**0.075**	1.05	0.96	1.01
**0.100**	1.76	0.67	1.22
**0.125**	0.81	1.12	0.97

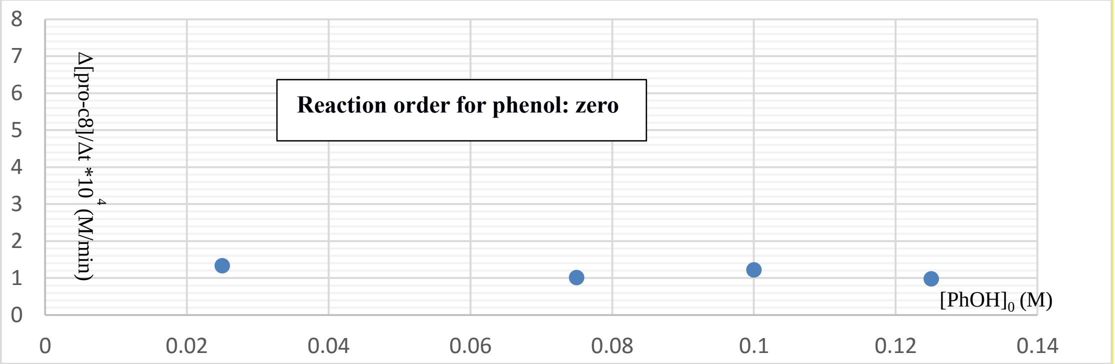

2. Order for dizao compound.

The procedure was followed as phenol

Plot of Δ[**pro-c8**]/Δt *10^5 vs initial concentration of diazo compound **2**.

**[Diazo]$_0$ (M)**	**Initial Δ[pro-c8]/Δt *10^5 (M/min)**		
	Run 1	Run 2	Average
**0.000**	0	0	0
**0.025**	0.58	0.67	0.63
**0.050**	2.45	1.15	1.80
**0.075**	3.16	1.89	2.53
**0.100**	4.11	2.55	3.33
**0.125**	4.37	4.46	4.42

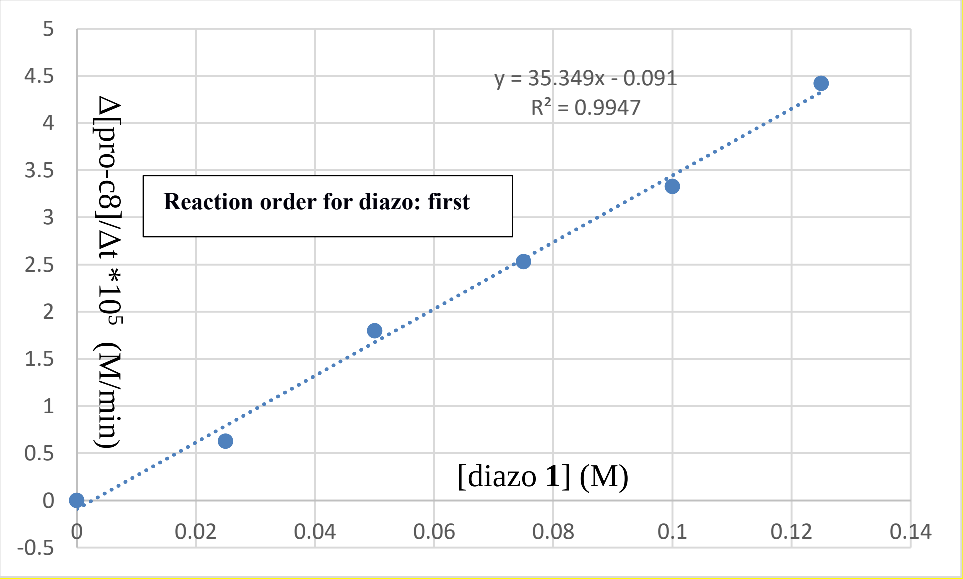

3. Order for gold catalyst.

The procedure was followed as phenol

Plot of Δ[**pro-c8**]/Δt $*10^4$ vs initial concentration of gold catalyst.

**[Diazo]$_0$ (M)**	**Initial Δ[pro-c8]/Δt $*10^4$ (M/min)**		
	Run 1	Run 2	Average
**0.00000**	0	0	0
**0.00125**	0.08	0.065	0.0725
**0.00250**	1.73	1.41	1.57
**0.00375**	3.13	2.97	3.05
**0.00500**	4.02	4.08	4.05
**0.00625**	4.63		4.63

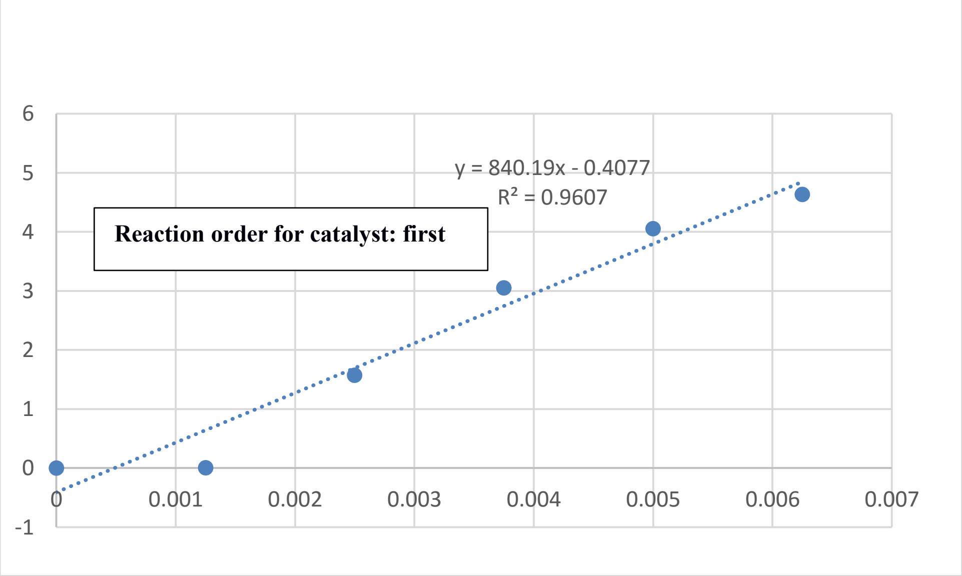

4. Order for H_2O

The procedure was followed as phenol

Plot of Δ[**pro-c8**]/Δt $*10^5$ vs volume of added H_2O.

**Volume of H_2O (μL)**	**Initial Δ[pro-c8]/Δt $*10^5$ (M/min)**		
	Run 1	Run 2	Average

**1**	-	1.08	1.08
**2**	1.40	1.70	1.45
**3**	1.03	1.00	1.02
**4**	1.46	1.17	1.32
**5**	1.76	1.12	1.44

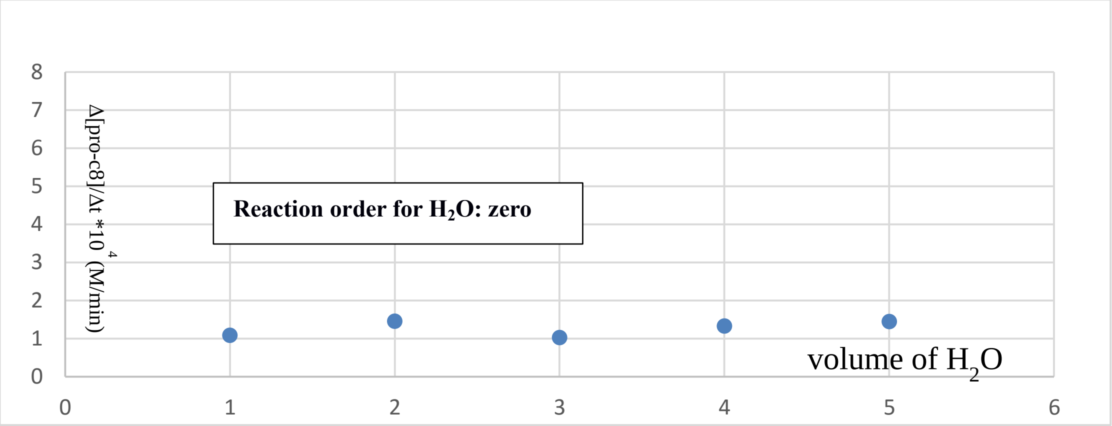

5. Explanation for reaction order of each components.

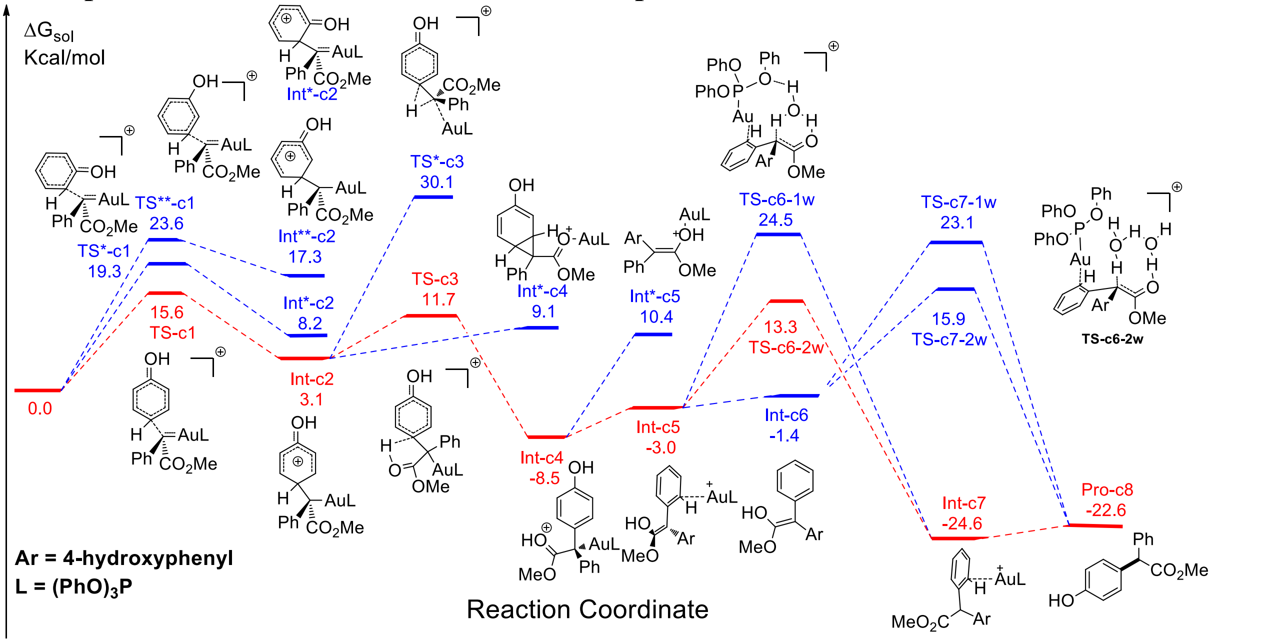

Figure 1. The calculated free energy profiles ΔG and the corresponding structures of intermediates and transition states along different possible C-H insertion pathways catalysed by $(PhO)_3PAuSbF_6$. The reasonable pathways are shown in red and other possible pathways are in blue. All values of free energies are relative to the reactants of Au-carbene and phenyl and in the units of kcal/mol.

Although there was huge difference between the reactions run at different temperatures, the results of kinetic studies at -45 °C provided certain reference significance to the mechanistic insight at room temperature. As shown in figure 1, the rate-determining step was transformation from **Int-**

c4 to **Int-c7**. As a result, the reaction rate is depended on the concentration of **Int-c4** and water. Besides, the concentration of **Int-c4** was determined by the process of formation of **Int-c2**. It is known that the generation rate of **Int-c2** was determined by concentration of phenol and gold carbene. However, compared to concentration of gold carbene, the concentration of phenol was regarded as constant. Based on this point, the formation rate of **Int-c2** is just determined by concentration of gold carbene, while the formation rate of gold carbene is relied on the concentration of diazo compound and gold catalyst. As a result, the reaction rate is depended on concentration of diazo compound, H_2O, gold catalyst. These results are consistent with the data of kinetic experiment except that of H_2O. The possible reason for zero order of H_2O is that H_2O is frozen to ice at -45 ^{o}C.

Reference:

1) An, L.; Xiao, Y.-L.; M, Q.-Q.; Zhang, X. *Angew. Chem., Int. Ed*. **2015**, *54*, 9079.

Section 6: Kinetic studies II.

To a dried glass tube was added phenol solution (0.3mmol, 0.3 mL, 1 M in CH_2Cl_2), gold catalyst (0.01 mmol, 0.01 M in CH_2Cl_2), which was preformed prior to be used. Subsequently, CH_2Cl_2 was added, keeping the reaction volume is equal to 3.8 mL. The reaction was cooled to -20 °C and diazo (0.2 mmol, 0.2 mL solution in CH_2Cl_2) was added and stirred at -20 °C. The reaction was quenched with diluted Et_3N solution in acetone at different time. Subsequently, dodecane (7.8 mg, 10 μL) was added as internal standard. The reaction mixture was monitored by GC-MS.

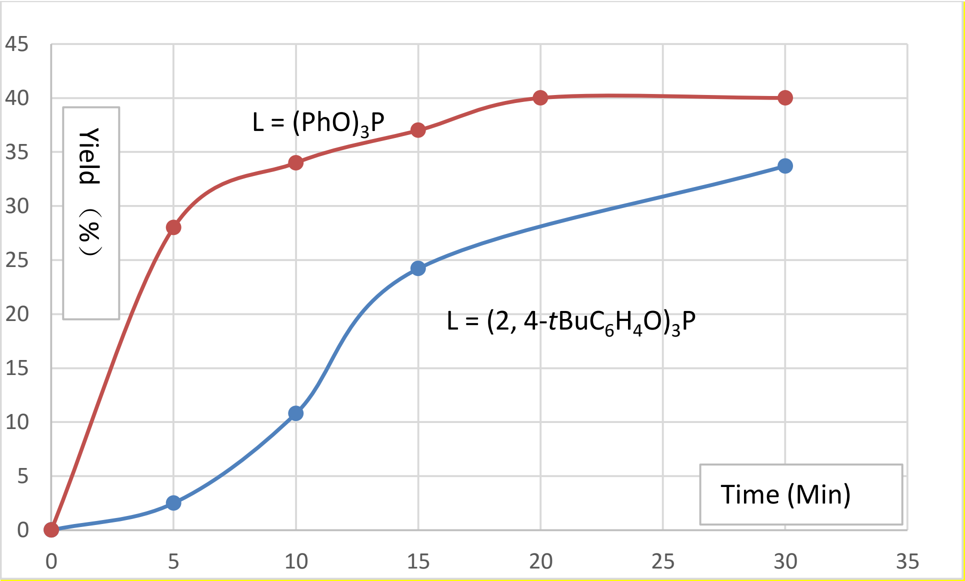

From the figure above, the more bulky ligand (2,4-*t*-$BuC_6H_4O)_3P$ exhibited lower reaction rate than smaller ligand $(PhO)_3P$ at -20 °C, which cannot be observed at room temperature due to the fast rates of the two reactions. These results supported our proposed mechanism that water severed as proton shuttle and the ligand oxygen was important for stabilizing transition state (**TS-c6-2w**) through H-bond. The lower rate of (2,4-*t*-$BuC_6H_4O)_3P$ was attributed to the worse H-bond due to the steric hindrance.

Section 7: Figures S1 and S2.

Figure S1 Calculated reaction pathways of diazoacetates and gold catalysts $(PhO)_3PAuSbF_6$ and $Ph_3PAuSbF_6$, denoted by the red and black lines respectively, to generate the products N_2 and Au-carbenes.

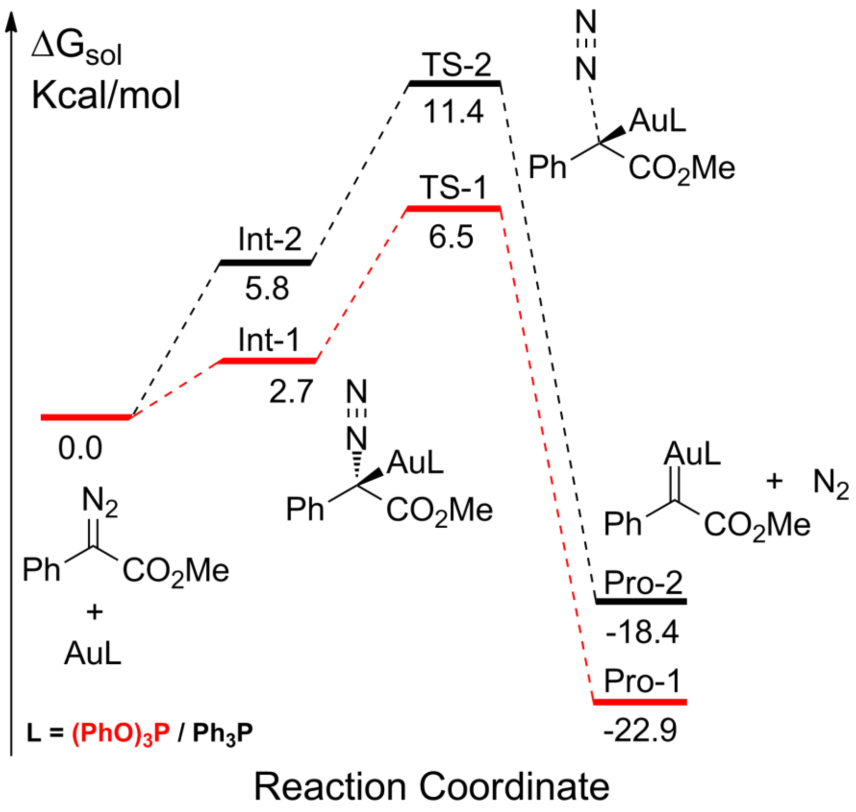

Figure S2 It shows the structure of located transition state **TS*-c6-2w**, where the water-assisted proton transfer occurs without forming hydrogen-interaction with the oxygen atom in the ligand $(PhO)_3PAu$.

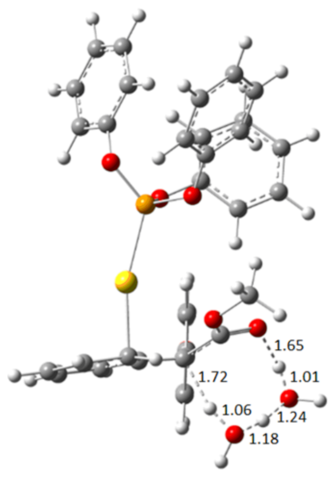

TS*-c6-2w
ΔG = 15.5

Section 8: Computational methods

All the Density Functional Theory (DFT) calculations were carried out in the Gaussian09 software package [1]. The geometric structures of intermediates and transition states were optimized with the M06 [2] functional method combined with the Lanl2dz basis set [3] for Au, P elements and 6-31G* basis set for C, N, O, H elements. The frequency analysis was also performed based on the optimized structures to verify the energy minima and transition states. The solvent effect of CH_2Cl_2 was evaluated using the PCM model [4] based on the gas-phase optimized structures. All discussed energy values are the Gibbs free energies calculated at the temperature 298 K and in the units of kcal/mol, including the corrections of solvation free energies estimated from the PCM model.

References

[1] Gaussian 09, Revision B.01, M. J. Frisch, G. W. Trucks, H. B. Schlegel, G. E. Scuseria, M. A. Robb, J. R. Cheeseman, G. Scalmani, V. Barone, B. Mennucci, G. A. Petersson, H. Nakatsuji, M. Caricato, X. Li, H. P. Hratchian, A. F. Izmaylov, J. Bloino, G. Zheng, J. L. Sonnenberg, M. Hada, M. Ehara, K. Toyota, R. Fukuda, J. Hasegawa, M. Ishida, T. Nakajima, Y. Honda, O. Kitao, H. Nakai, T. Vreven, J. A. Montgomery, Jr., J. E. Peralta, F. Ogliaro, M. Bearpark, J. J. Heyd, E. Brothers, K. N. Kudin, V. N. Staroverov, R. Kobayashi, J. Normand, K. Raghavachari, A. Rendell, J. C. Burant, S. S. Iyengar, J. Tomasi, M. Cossi, N. Rega, J. M. Millam, M. Klene, J. E. Knox, J. B. Cross, V. Bakken, C. Adamo, J. Jaramillo, R. Gomperts, R. E. Stratmann, O. Yazyev, A. J. Austin, R. Cammi, C. Pomelli, J. W. Ochterski, R. L. Martin, K. Morokuma, V. G. Zakrzewski, G. A. Voth, P. Salvador, J. J. Dannenberg, S. Dapprich, A. D. Daniels, Ö. Farkas, J. B. Foresman, J. V. Ortiz, J. Cioslowski, and D. J. Fox, Gaussian, Inc., Wallingford CT, **2009**.

[2] Y. Zhao, D. G. Truhlar, Theor. Chem. Acc. **2008**, *120*, 215.

[3] a) P. J. Hay, W. R. Wadt, J. Chem. Phys. **1985**, *82*, 299; b) W. R. Wadt, P. J. Hay, J. Chem. Phys. **1985**, *82*, 284.

[4] G. Scalmani, M. J. Frisch, J. Chem. Phys. **2010**, *132*, 114110.

Section 9: Cartesian coordinates of structures in Figure 1

Structure and coordinates of **TS-c1p**

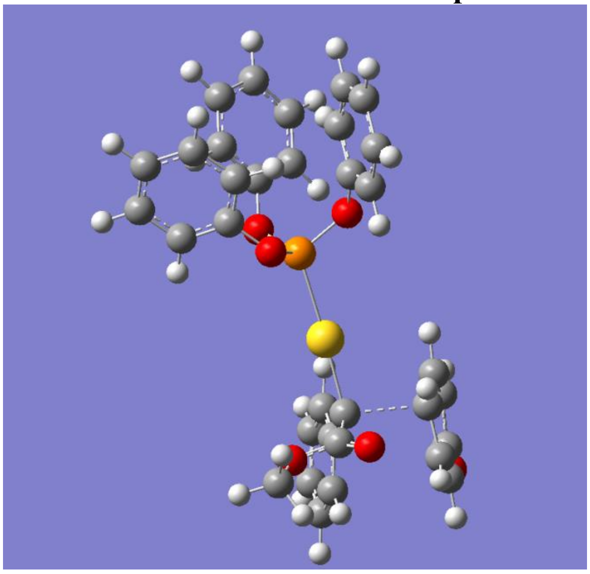

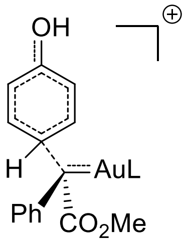

TS-c1p

L = $(PhO)_3P$

C	0.34089300	-3.39565900	3.41676600
C	0.95233400	-2.33023700	2.77817700
C	1.06594900	-1.06904800	3.40693900
C	0.50234500	-0.92545700	4.69340900
C	-0.11660300	-1.98863300	5.33011900
C	-0.19840900	-3.22578600	4.69151000
H	0.27841000	-4.36236900	2.92234900
H	1.37074200	-2.47026700	1.78208200
H	0.57630400	0.04198000	5.19255300
H	-0.52596400	-1.86072400	6.33003700
H	-0.68082800	-4.06364700	5.19137400
C	1.70891200	0.04631500	2.75804400
C	2.66514700	-0.19826000	1.62441500
O	2.66796000	0.33234200	0.53797800
O	3.61823400	-1.03645700	2.04993100
C	4.71538600	-1.21840300	1.14544600
H	5.39495700	-1.91047300	1.64390000
H	4.36315600	-1.63520300	0.19685300
H	5.20901000	-0.26046200	0.95316900
C	-0.24700900	-0.03472700	0.77364300
C	0.11675600	1.06171500	1.59646400
C	-0.74441600	1.41537000	2.66758500
C	-1.75090200	0.57135800	3.05941000
C	-1.97301900	-0.61777700	2.33052900
C	-1.25618200	-0.88549600	1.14941500
H	0.33669500	-0.22913800	-0.12281300
H	-0.55741100	2.33521700	3.22138700
H	-2.37952400	0.80163900	3.91995600
H	-1.51863300	-1.77182500	0.57631100
O	-2.87027800	-1.52405300	2.70703200
H	-3.32201900	-1.25044000	3.52153400
H	0.81748700	1.79398800	1.20116300
Au	2.40724700	1.68941900	3.82812900
P	3.35334600	3.57994400	5.05372000
O	2.49257700	4.99371400	4.83771700
O	3.41533000	3.31132900	6.70003900
O	4.90972800	3.88937000	4.54719800
C	5.96754800	4.26859400	5.38594600

C	6.38436200	5.59172100	5.38626000
C	6.61547500	3.29256000	6.13224300
C	7.47286100	5.94662600	6.17736900
H	5.86959800	6.31931600	4.76267500
C	7.70008500	3.66330100	6.92098600
H	6.27085600	2.26082500	6.09059300
C	8.12606200	4.98920900	6.94849100
H	7.81266500	6.98016500	6.18468800
H	8.21711500	2.90923400	7.51079000
H	8.97754700	5.27345700	7.56322400
C	3.08288000	6.25840500	4.69173600
C	3.55879800	6.63022200	3.44096500
C	3.11129600	7.12445600	5.77519900
C	4.09772600	7.90246800	3.27951300
H	3.50693900	5.92550400	2.61297700
C	3.65028400	8.39512600	5.59791100
H	2.70874000	6.80547100	6.73394800
C	4.14785200	8.78385200	4.35739100
H	4.47552100	8.20663600	2.30557300
H	3.67693800	9.08474600	6.43905500
H	4.56841200	9.77870700	4.22648700
C	3.19829700	4.30129100	7.66819900
C	1.88915600	4.62568400	8.00121400
C	4.28293100	4.87361200	8.31678200
C	1.66231300	5.56742400	8.99903800
H	1.06680400	4.14353100	7.47566800
C	4.04079700	5.81194900	9.31591600
H	5.29425600	4.57505600	8.04921900
C	2.73701800	6.16456000	9.65378800
H	0.64147100	5.83216300	9.26687500
H	4.88178000	6.26810100	9.83403100
H	2.55760500	6.90008900	10.43508800

Structure and coordinates of **TS-c1o**

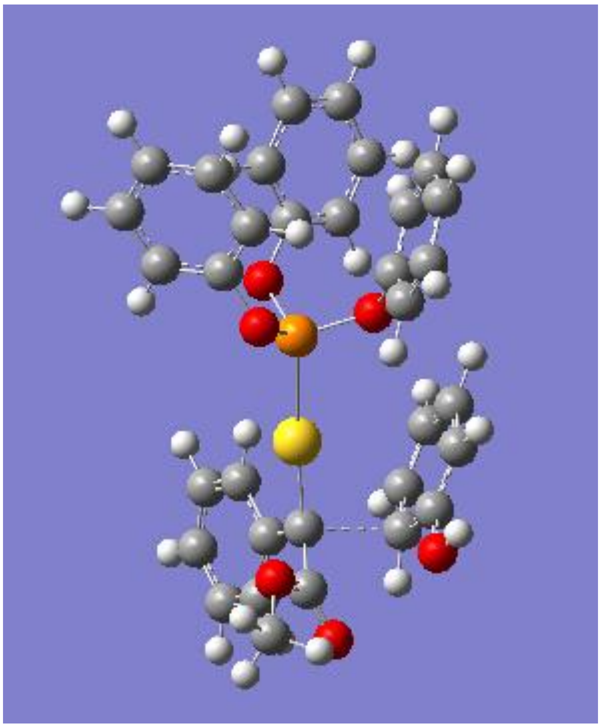

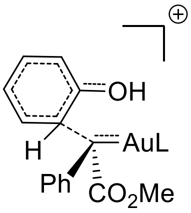

TS-c1o
L = (PhO)$_3$P

C	0.56058300	-3.60808900	3.48322100
C	0.92881800	-2.49572900	2.74268200
C	1.33139300	-1.30379300	3.38483000
C	1.33125800	-1.27948400	4.79635200
C	0.96652500	-2.39324800	5.53183300
C	0.57955500	-3.56208200	4.87541500
H	0.25797100	-4.51923500	2.97176200
H	0.90177300	-2.53897300	1.65676600
H	1.62617500	-0.36295200	5.30927100
H	0.98477600	-2.35652200	6.61870600
H	0.29262700	-4.43907200	5.45254600
C	1.73436200	-0.11246400	2.64073700
C	2.21943600	-0.39766500	1.22919000
O	1.57553200	-0.86797900	0.32086900
O	3.51203300	-0.08433100	1.13922100
C	4.12272300	-0.39159900	-0.12127500
H	5.16078600	-0.07015600	-0.03268100
H	4.06466700	-1.46622300	-0.31893000
H	3.60912800	0.14830500	-0.92329800
C	-0.87148300	0.74339800	3.24735700
C	-0.10847300	0.76680900	2.04741900
C	0.32723000	2.03450500	1.56481500
C	0.15915500	3.18547000	2.34004500
C	-0.51973000	3.09681400	3.54289300
C	-1.05000000	1.87573500	4.00237500
H	0.55881900	4.14055600	1.99869100
H	-0.22461900	-0.02927900	1.31126400
Au	2.71088800	1.45575100	3.62086800
P	3.64641500	3.35108800	4.84111900
O	2.75167800	4.71840100	4.46253700
O	3.46286000	3.10931400	6.48514600
O	5.24851600	3.76338000	4.63148700
C	6.07785700	4.20315800	5.67542400
C	6.37910100	5.55384900	5.77891000
C	6.63055100	3.25972600	6.53199400

C	7.25150100	5.96653400	6.78191100
H	5.94655600	6.26223700	5.07547300
C	7.49620000	3.68791000	7.53294400
H	6.37917100	2.20771200	6.41056500
C	7.80463600	5.04031600	7.66141000
H	7.50045600	7.02194700	6.87087200
H	7.93360300	2.95932900	8.21236900
H	8.48544700	5.37086900	8.44299500
C	3.26673100	6.02064900	4.43982400
C	3.98645300	6.43445600	3.32517200
C	2.97947400	6.88437600	5.48749900
C	4.44703800	7.74596700	3.26976400
H	4.18963200	5.72711600	2.52281400
C	3.44453200	8.19427400	5.41731200
H	2.39665100	6.53169400	6.33615400
C	4.18033600	8.62503200	4.31667200
H	5.01656800	8.07950400	2.40472000
H	3.22640400	8.88205700	6.23167800
H	4.54105600	9.65034300	4.27057200
C	2.93993400	4.05950100	7.36579400
C	1.56142300	4.22349200	7.42402800
C	3.79735300	4.75467300	8.20650800
C	1.02868300	5.12528700	8.33878400
H	0.92864500	3.64157900	6.75388700
C	3.25019400	5.65001600	9.12094700
H	4.87062800	4.58611800	8.14309800
C	1.87250500	5.84135100	9.18536500
H	-0.04908300	5.26421300	8.39598100
H	3.90918800	6.20058100	9.78904300
H	1.45376100	6.54351000	9.90319600
H	-0.64009200	3.99864300	4.14177600
H	-1.60494500	1.84102500	4.93641000
H	-1.26355200	-0.21418800	3.58764300
O	0.95166100	2.05088100	0.38726800
H	1.23374500	2.95245600	0.16310400

Structure and coordinates of **TS-c1m**

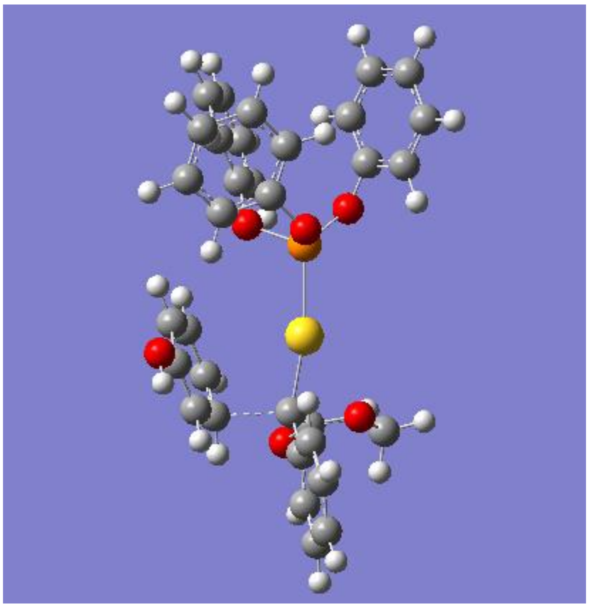

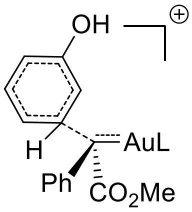

TS-c1m

L = $(PhO)_3P$

C	0.75222600	-3.54130800	3.40795200
C	1.04314000	-2.36803300	2.73078400
C	1.11176000	-1.13414400	3.40719800
C	0.83892800	-1.12609700	4.78765600
C	0.54706800	-2.29773300	5.46858400
C	0.50499100	-3.50773600	4.77901300
H	0.71611700	-4.48499300	2.86835800
H	1.22885000	-2.40213800	1.65665700
H	0.87200200	-0.17781100	5.32742800
H	0.35579800	-2.27065700	6.53897900
H	0.27492000	-4.42850300	5.31153200
C	1.45652900	0.09915100	2.70545300
C	2.30253900	-0.00952900	1.46167500
O	2.09393700	0.49130500	0.38012600
O	3.41617600	-0.68296200	1.76999400
C	4.41615800	-0.71291100	0.74434300
H	5.24620100	-1.28367700	1.16202200
H	4.02605500	-1.19714700	-0.15614800
H	4.73243800	0.30488800	0.49466100
C	-0.65047200	-0.15122000	1.07466000
C	-0.22642000	0.90669300	1.92496600
C	-0.94900600	1.15743000	3.12596500
C	-1.86622000	0.23344400	3.57462300
C	-2.13877700	-0.90098300	2.80580800
C	-1.56565500	-1.07856200	1.53595500
H	-0.16967000	-0.26232800	0.10358100
H	-0.70574400	2.03871200	3.71831400
H	-2.37735900	0.36979200	4.52471600
H	0.31497700	1.72664200	1.45244200
Au	2.26086000	1.70119900	3.79952900
P	3.28763700	3.53386400	5.04428400
O	2.47368900	4.97495300	4.83694600
O	3.32459100	3.24416000	6.68795400
O	4.86019100	3.79544700	4.56070300
C	5.91126000	4.14981000	5.41960300
C	6.36388700	5.46127700	5.42682200
C	6.51551200	3.15819200	6.18181200
C	7.44371700	5.78733800	6.24193500

H	5.88334600	6.20310100	4.79228900
C	7.59215900	3.50020600	6.99391500
H	6.14040500	2.13740300	6.13531700
C	8.05320700	4.81415000	7.02904500
H	7.81111600	6.81133100	6.25596300
H	8.07463400	2.73372500	7.59685700
H	8.89785400	5.07701600	7.66239700
C	3.09999100	6.22447400	4.71612100
C	3.59870300	6.60070200	3.47556300
C	3.13787400	7.07492400	5.81154700
C	4.17100100	7.86094600	3.33693000
H	3.53703600	5.90885400	2.63745600
C	3.71090400	8.33361900	5.65714500
H	2.71648400	6.75377000	6.76139200
C	4.23129400	8.72628100	4.42722900
H	4.56592700	8.16899100	2.37103400
H	3.74513400	9.01133600	6.50763400
H	4.67663200	9.71247300	4.31378800
C	3.10669300	4.22680700	7.66359500
C	1.79717100	4.56373600	7.98224500
C	4.18950800	4.78061900	8.33130600
C	1.56800200	5.49876700	8.98578900
H	0.97616900	4.09559000	7.44198000
C	3.94488100	5.71245100	9.33598200
H	5.20108600	4.47462500	8.07280300
C	2.64091000	6.07697700	9.66022100
H	0.54684700	5.77290300	9.24266100
H	4.78415200	6.15429500	9.86904600
H	2.45968300	6.80747600	10.44579800
O	-1.94666400	-2.17304500	0.85160600
H	-1.54921800	-2.17904100	-0.03194600
H	-2.83341000	-1.66189300	3.15907900

Structure and coordinates of **Int-c2p**

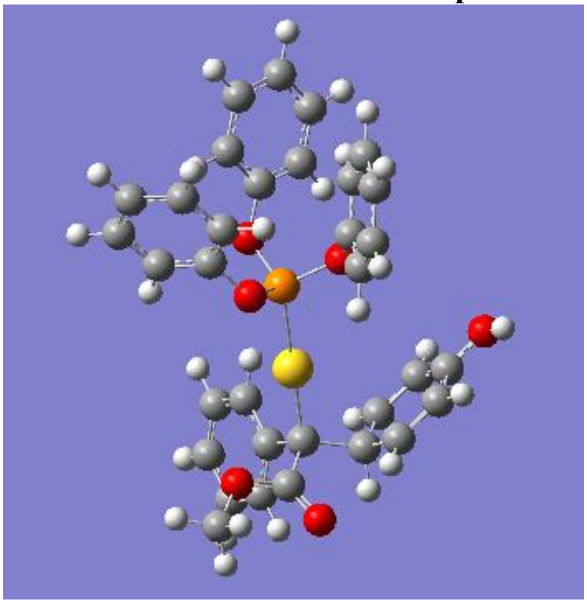

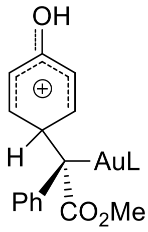

Int-c2p

L = $(PhO)_3P$

C	0.85008300	-3.58316300	3.43807100
C	1.07268700	-2.39300000	2.75591900
C	1.27404300	-1.19113300	3.44719000
C	1.22554900	-1.21572600	4.84316500
C	0.99753500	-2.40479300	5.52950300
C	0.81087300	-3.59181100	4.82934700
H	0.70689400	-4.50725900	2.88124700
H	1.10245800	-2.40044500	1.66356400
H	1.37182200	-0.28959200	5.40260000
H	0.97165300	-2.40176000	6.61747300
H	0.63679800	-4.52254400	5.36546100
C	1.46532400	0.08284200	2.64493700
C	2.42220400	-0.16705100	1.50693900
O	2.16004900	-0.02806100	0.32759700
O	3.60802200	-0.58860000	1.95087400
C	4.56049100	-0.92472800	0.94019400
H	5.44476300	-1.27595300	1.47350300
H	4.16610200	-1.71171500	0.28910900
H	4.80126800	-0.04669300	0.33204200
C	-0.99697500	0.49246000	3.01579000
C	0.07739100	0.53899900	2.00973200
C	0.22075700	1.84502600	1.34161100
C	-0.52564600	2.93019200	1.67306900
C	-1.50899200	2.80417500	2.68576800
C	-1.75065800	1.56972600	3.34438500
H	-1.16500800	-0.45855600	3.51922100
H	0.97143900	1.90582000	0.55627700
H	-0.38302300	3.89134400	1.17960900
H	-2.53677500	1.53389100	4.09435100
O	-2.25317800	3.81492900	3.06396500
H	-2.03432900	4.63278700	2.58259900
H	-0.13686600	-0.21249800	1.21921300
Au	2.28636900	1.68938100	3.76172800
P	3.25493000	3.56897500	5.00782100
O	2.41325800	4.99820200	4.75159200
O	3.26834300	3.32364900	6.66081900
O	4.82667500	3.90563000	4.55627500
C	5.86609100	4.21027400	5.44358000

C	6.30752300	5.52249800	5.54083400
C	6.47172500	3.17740600	6.14785500
C	7.38041400	5.80299600	6.38156800
H	5.81919600	6.30221900	4.95965200
C	7.53893500	3.47419600	6.98921900
H	6.10563400	2.15852200	6.03252000
C	7.99216800	4.78585100	7.10940400
H	7.74068400	6.82636400	6.46436400
H	8.02158300	2.67427300	7.54689300
H	8.83033800	5.01376300	7.76448300
C	3.02958100	6.25060400	4.63734800
C	3.62933700	6.59749800	3.43247200
C	2.96152000	7.14004500	5.70100400
C	4.19190200	7.86251200	3.29927900
H	3.66452400	5.87272900	2.62090500
C	3.52594600	8.40341600	5.55291400
H	2.46221100	6.84399200	6.62114800
C	4.14470900	8.76505500	4.35955100
H	4.66943800	8.14243800	2.36244200
H	3.47776600	9.10911400	6.37974700
H	4.58520400	9.75401800	4.25135600
C	3.04583100	4.32519700	7.61328800
C	1.73413600	4.66247100	7.92383000
C	4.12409600	4.89520600	8.27484500
C	1.49881000	5.61597300	8.90857600
H	0.91676100	4.17656600	7.39368500
C	3.87371500	5.84699000	9.25901900
H	5.13696900	4.58570100	8.02690900
C	2.56771700	6.21333600	9.57272400
H	0.47582300	5.88786900	9.16113900
H	4.71012500	6.30171100	9.78579400
H	2.38188700	6.95744000	10.34440100

Structure and coordinates of **Int-c2o**

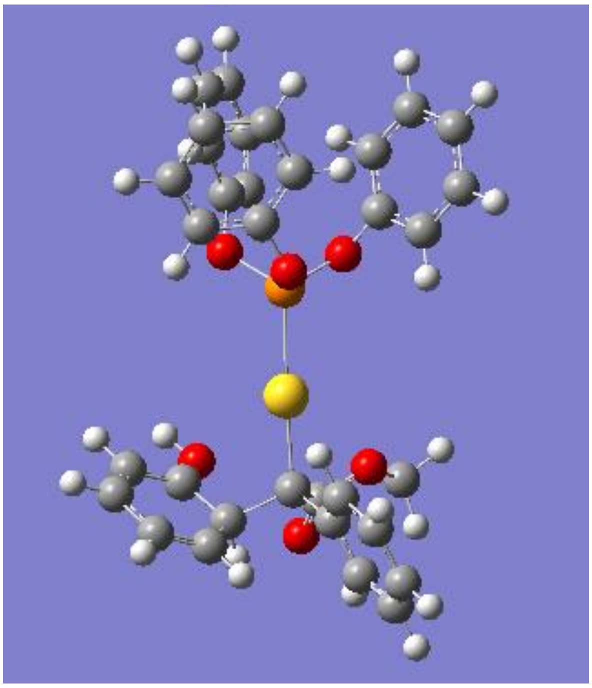

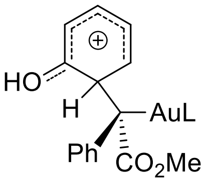

Int-c2o

L = $(PhO)_3P$

C	1.58453300	-3.51428900	3.37983300
C	1.53739400	-2.31263400	2.68063600
C	1.51118600	-1.08505100	3.35675600
C	1.51598900	-1.10383600	4.75612800
C	1.55439300	-2.30409900	5.45682300
C	1.59127100	-3.51433100	4.77055100
H	1.61259100	-4.45455400	2.83238500
H	1.51968200	-2.33309900	1.58966800
H	1.48362500	-0.16081100	5.30601200
H	1.56163600	-2.29196600	6.54501400
H	1.62629500	-4.45371800	5.31853700
C	1.39471500	0.20820400	2.56532700
C	2.18160600	0.03491000	1.28159100
O	1.68952400	-0.17652900	0.19149300
O	3.49437200	0.05906100	1.50496400
C	4.31425800	-0.27319000	0.38102500
H	5.34265200	-0.22655000	0.74114400
H	4.07650400	-1.28116700	0.02450500
H	4.15527300	0.44147100	-0.43247100
C	-1.10986500	-0.08891100	3.05687400
C	-0.12211700	0.45175800	2.11381700
C	-0.36887700	1.84903400	1.71515100
C	-1.41347300	2.59897200	2.25540700
C	-2.26702200	2.00280700	3.16187400
C	-2.13572100	0.64726500	3.55593400
H	-1.54652300	3.64063100	1.96584400
H	-0.16147700	-0.14315200	1.16619300
Au	2.16476300	1.86139000	3.64970400
P	3.17240900	3.70555200	4.91038700
O	2.61324100	5.23061700	4.49975600
O	2.94903800	3.54551700	6.55900500
O	4.82305300	3.76630800	4.65807100
C	5.77318200	3.93615600	5.67049000
C	6.39232800	5.16944000	5.81959900
C	6.11626700	2.84228300	6.45480200
C	7.37632500	5.30638700	6.79461000

H	6.10733100	5.99985600	5.17630800
C	7.09700700	2.99592800	7.42923700
H	5.61599900	1.88801900	6.29610100
C	7.72517100	4.22717700	7.60193500
H	7.87250100	6.26655200	6.92114500
H	7.37404800	2.14719200	8.05116700
H	8.49460100	4.34296700	8.36245100
C	3.43228700	6.36593800	4.42901300
C	4.23382100	6.54472800	3.30770300
C	3.36677200	7.31560500	5.43911100
C	5.00275800	7.69934100	3.20658500
H	4.25720100	5.77839300	2.53512700
C	4.13673900	8.46920600	5.32112200
H	2.71495800	7.15302800	6.29530300
C	4.95713100	8.66116300	4.21309200
H	5.63777500	7.84760400	2.33561700
H	4.08985000	9.22323100	6.10421100
H	5.55735900	9.56476100	4.13027600
C	2.78486800	4.61514900	7.44643200
C	1.52483600	5.18757300	7.57039700
C	3.85305100	5.01997800	8.23432800
C	1.33784700	6.21137200	8.49338400
H	0.70877800	4.82889300	6.94553300
C	3.65119300	6.04505500	9.15379100
H	4.81871600	4.52968400	8.13232800
C	2.40164000	6.64517200	9.28121800
H	0.35562100	6.66755200	8.60041800
H	4.48070000	6.37059000	9.77819100
H	2.25238100	7.44343700	10.00522300
H	-3.07689800	2.59779200	3.58248600
H	-2.85011600	0.21803300	4.25280500
H	-0.98131400	-1.12800500	3.35384600
O	0.46072000	2.34398100	0.82956300
H	0.25301900	3.27202200	0.62022000

Structure and coordinates of **Int-c2m**

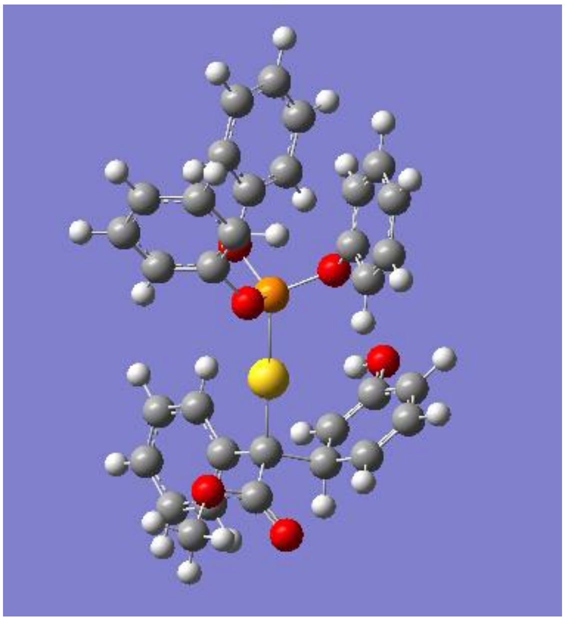

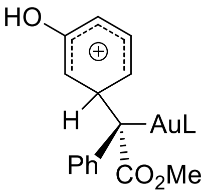

Int-c2m
L = (PhO)$_3$P

C	1.44926800	-3.54789400	3.52458200
C	1.47499100	-2.35738900	2.81060800
C	1.26100500	-1.12673100	3.44606900
C	0.99505500	-1.13840600	4.81935800
C	0.96025300	-2.32834900	5.53851400
C	1.19022300	-3.53775000	4.89263800
H	1.63541600	-4.48957200	3.01154300
H	1.69014600	-2.38701300	1.74150900
H	0.82114700	-0.19229700	5.33699900
H	0.76211700	-2.30691200	6.60831100
H	1.17102900	-4.47034300	5.45285400
C	1.26280900	0.15906400	2.66342100
C	2.15684700	0.19933200	1.45230800
O	1.84698100	0.66403600	0.37229600
O	3.37118600	-0.28503300	1.72505100
C	4.32767500	-0.16975500	0.66796200
H	5.24946300	-0.60439200	1.05673000
H	3.98841000	-0.71340500	-0.21953000
H	4.47859200	0.88221100	0.40470800
C	-0.70844300	-0.56981900	1.24830900
C	-0.25002800	0.51602900	2.09941400
C	-1.10259800	0.86606800	3.21781500
C	-2.18253200	0.09107100	3.55639500
C	-2.48967300	-1.03036400	2.77804200
C	-1.77135500	-1.36198100	1.61076400
H	-0.13577500	-0.76496000	0.34111400
H	-0.82371500	1.73089200	3.82024100
H	-2.79794500	0.32849600	4.42006200
H	0.01448600	1.39006400	1.47639200
Au	2.04010400	1.80246900	3.78009800
P	3.08527900	3.63637300	5.01449000
O	2.42872100	5.13998300	4.70463100
O	3.00486300	3.41702000	6.66781900
O	4.70015800	3.75081200	4.60804600
C	5.73749200	3.99245500	5.51682800
C	6.30258200	5.25836400	5.58164300
C	6.21674700	2.93249900	6.27591600
C	7.37064300	5.46490400	6.44954000

H	5.91339500	6.05818500	4.95453100
C	7.28161600	3.15562200	7.14250000
H	5.75019200	1.95279800	6.18448500
C	7.85664200	4.42141200	7.23294600
H	7.82576900	6.45134900	6.51087300
H	7.66455600	2.33522400	7.74592000
H	8.69070900	4.59209200	7.91026700
C	3.18687400	6.31211900	4.56825600
C	3.80812800	6.56641500	3.35192500
C	3.23391600	7.21715400	5.61886900
C	4.51070600	7.75633100	3.19304100
H	3.73846800	5.83562600	2.54810000
C	3.93644600	8.40581700	5.44336000
H	2.71911400	6.99290000	6.55083200
C	4.57842700	8.67507600	4.23786400
H	5.00290200	7.96636600	2.24563400
H	3.97799400	9.12601500	6.25788800
H	5.12437300	9.60711800	4.10783500
C	2.89823200	4.45498100	7.60370700
C	1.64326400	4.99222700	7.86232800
C	4.02666700	4.86132500	8.30148100
C	1.52227700	5.98033300	8.83431200
H	0.77931500	4.63964100	7.30184600
C	3.88975900	5.84830300	9.27296000
H	4.98932100	4.40148500	8.08899300
C	2.64474000	6.41194400	9.53706800
H	0.54535700	6.41163000	9.04321500
H	4.76682600	6.17330700	9.82890900
H	2.54545800	7.18243500	10.29877000
O	-2.21121600	-2.43459300	0.93485300
H	-1.67306800	-2.59581400	0.14519800
H	-3.32645000	-1.67520200	3.04641900

Structure and coordinates of **TS-c3**

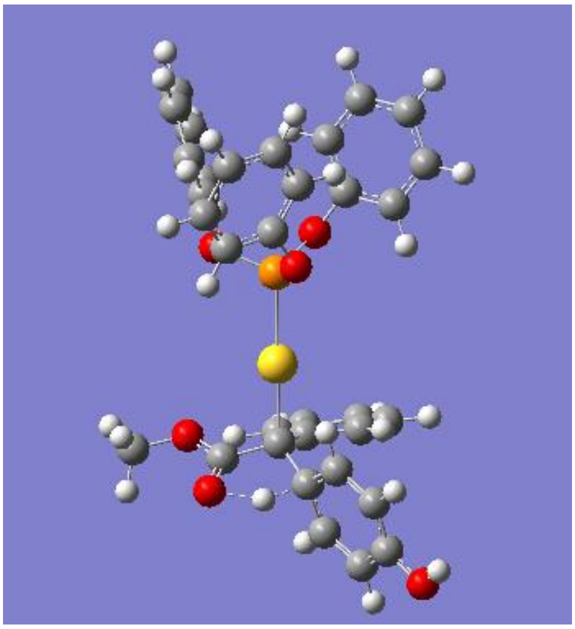

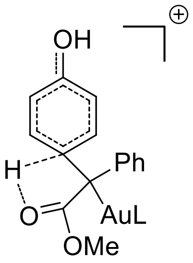

TS-c3
L = (PhO)$_3$P

C	2.34375600	-3.11246300	4.71402400
C	2.40305800	-2.13767800	3.72096000
C	1.44808300	-1.11726100	3.67005500
C	0.43957900	-1.10611900	4.64702300
C	0.38306800	-2.07970000	5.63365000
C	1.33889700	-3.09167900	5.67296800
H	3.09568700	-3.89937400	4.73006400
H	3.20362800	-2.17650700	2.98806300
H	-0.31314600	-0.31466800	4.63044700
H	-0.41029300	-2.04541300	6.37801800
H	1.29797600	-3.85659500	6.44536700
C	1.42888800	-0.02433000	2.62535900
C	2.31468600	-0.08512200	1.43591600
O	1.86165000	0.28709900	0.33397700
O	3.55911800	-0.47526800	1.57369600
C	4.39031100	-0.40142600	0.40239500
H	5.36927000	-0.76040000	0.72025700
H	3.98160400	-1.03365200	-0.39076600
H	4.45026600	0.63370900	0.05406300
C	-0.74252600	-0.94562300	1.68120900
C	0.02862400	0.22477900	2.02426300
C	-0.72619600	1.41673900	2.28129600
C	-2.08278600	1.47624300	2.08702800
C	-2.77587200	0.31655500	1.69085100
C	-2.09376200	-0.89982300	1.48477200
H	-0.20612000	-1.88459400	1.54075500
H	-0.18458400	2.31156500	2.59243000
H	-2.62763200	2.40623100	2.24697700
H	-2.66986800	-1.77343800	1.19030600
O	-4.08501000	0.30146100	1.48887600
H	-4.48139900	1.17358100	1.64896700
H	0.55501100	0.46569100	0.88352200
Au	2.26979800	1.63959700	3.68981800
P	3.28206600	3.44560100	4.98170200
O	2.46879700	4.89381300	4.80325500
O	3.33009200	3.13400500	6.62079300
O	4.85601200	3.72758600	4.50575400

C	5.90093200	4.05722900	5.38011700
C	6.36591700	5.36421500	5.41305200
C	6.48951900	3.04825200	6.13211500
C	7.44261300	5.66640900	6.24131500
H	5.89581500	6.12155800	4.78880400
C	7.56280600	3.36635800	6.95796800
H	6.10078600	2.03338400	6.06944400
C	8.03702900	4.67480100	7.01703200
H	7.81955700	6.68650000	6.27542100
H	8.03196100	2.58578600	7.55334600
H	8.87902600	4.91947900	7.66111600
C	3.10063700	6.14101000	4.70480400
C	3.60692900	6.53448200	3.47244600
C	3.13734400	6.97559000	5.81282500
C	4.18474100	7.79417000	3.35446600
H	3.54754600	5.85466400	2.62425600
C	3.71601900	8.23414800	5.67924300
H	2.71175400	6.64176300	6.75660100
C	4.24323300	8.64322100	4.45760000
H	4.58574600	8.11476500	2.39511600
H	3.74960200	8.89886700	6.54000100
H	4.69299000	9.62919300	4.36074300
C	3.09395000	4.09441800	7.61275800
C	1.77932300	4.41221300	7.93001600
C	4.16723600	4.64696600	8.29728200
C	1.53546500	5.32588600	8.94965500
H	0.96629400	3.94350700	7.37825000
C	3.90773300	5.55665000	9.31826900
H	5.18345400	4.35681200	8.03852600
C	2.59836500	5.90204900	9.64151100
H	0.51010500	5.58421400	9.20636000
H	4.73954700	5.99626300	9.86475900
H	2.40499400	6.61527300	10.43998100

Structure and coordinates of **TS-c3A**

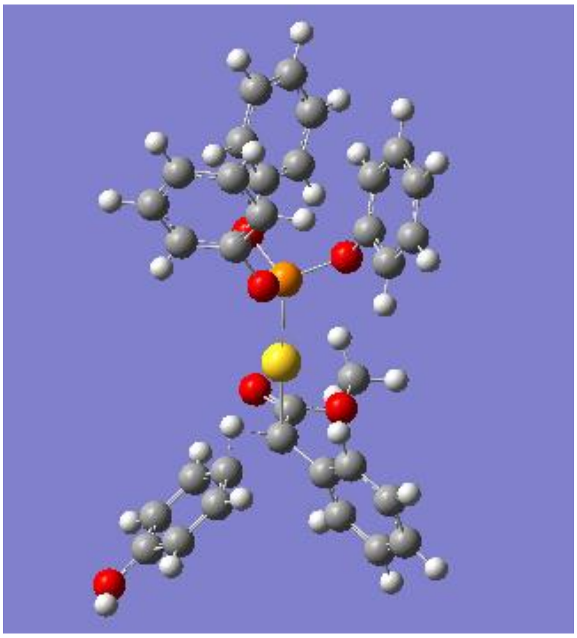

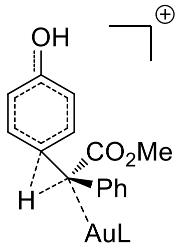

TS-c3A

L = $(PhO)_3P$

C	-0.43694400	-3.00485900	4.60433300
C	0.26731000	-2.22607900	3.70002600
C	0.62361200	-0.90862900	4.01638500
C	0.22953000	-0.38555000	5.24713300
C	-0.49100600	-1.16257800	6.15183700
C	-0.82056300	-2.47324900	5.83510100
H	-0.69260000	-4.03129600	4.34894300
H	0.55574900	-2.64215000	2.73350200
H	0.47710400	0.64539100	5.50949300
H	-0.79145400	-0.73724800	7.10715700
H	-1.37962100	-3.08255700	6.54213400
C	1.39655900	-0.13663100	2.97832400
C	2.72698600	-0.67846200	2.52405600
O	3.31412200	-0.31696500	1.52624400
O	3.21168300	-1.55126100	3.40335200
C	4.52842300	-2.03802900	3.12000300
H	4.78068000	-2.69880900	3.94988500
H	4.53738600	-2.58574800	2.17286200
H	5.23535000	-1.20437900	3.05747400
C	0.72210300	-0.26319600	0.35978100
C	0.39941700	0.10639600	1.68688800
C	-0.92947800	0.48289500	1.99141700
C	-1.90581400	0.46563800	1.02344000
C	-1.57833600	0.06663000	-0.28240400
C	-0.25954600	-0.28875900	-0.60371400
H	1.74797600	-0.50955100	0.10334200
H	-1.17962000	0.78479100	3.00841700
H	-2.92671000	0.75867800	1.26645900
H	-0.03575500	-0.57266100	-1.62916200
O	-2.47290700	0.01921100	-1.26961900
H	-3.35188500	0.28617800	-0.95829800
H	1.10504400	0.98107000	2.24006700
Au	2.27808900	1.67863500	3.93149500
P	3.32715400	3.58246800	5.02565900
O	2.21811300	4.75936100	5.42945900
O	4.08572600	3.09796600	6.42663700

O	4.47943600	4.31225300	4.07264400
C	5.70362200	4.80112700	4.55678000
C	5.86307600	6.16649000	4.74201300
C	6.74172500	3.90239600	4.76322100
C	7.10333400	6.63784700	5.16272500
H	5.03162600	6.84080400	4.54739300
C	7.97362400	4.38791500	5.18908200
H	6.57601600	2.83985000	4.59482200
C	8.15430400	5.75420800	5.39234900
H	7.24610400	7.70658700	5.30842600
H	8.79491700	3.69456700	5.35745300
H	9.11977700	6.13141200	5.72277500
C	2.49795000	6.13611800	5.40262400
C	2.40070700	6.80704500	4.19055400
C	2.78830700	6.79503200	6.58812400
C	2.62220200	8.18002900	4.16583300
H	2.15792300	6.25373000	3.28511200
C	3.00376600	8.16934500	6.54801500
H	2.83109600	6.23783400	7.52151500
C	2.92777300	8.86104900	5.34218600
H	2.55135600	8.71854300	3.22308700
H	3.22964300	8.70063600	7.47026500
H	3.09740400	9.93538100	5.31927300
C	4.12291700	3.84290100	7.61596000
C	3.02838400	3.78357700	8.46910300
C	5.26958400	4.55487600	7.93666700
C	3.08001100	4.47831500	9.67309100
H	2.15405800	3.20049600	8.18475000
C	5.30776400	5.24080500	9.14700300
H	6.11519400	4.56036600	7.25192400
C	4.21634000	5.20980600	10.01078500
H	2.22925400	4.44390800	10.35040600
H	6.20075300	5.80187400	9.41450600
H	4.25387000	5.74943800	10.95472900

Structure and coordinates of **Int-c4**

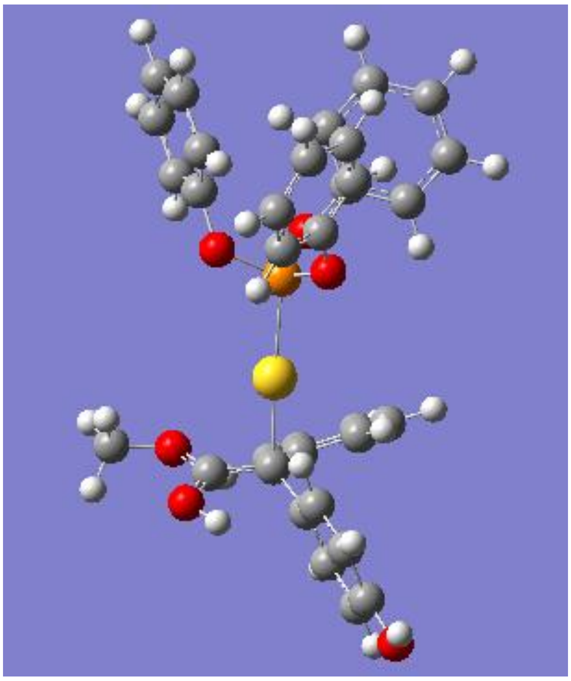

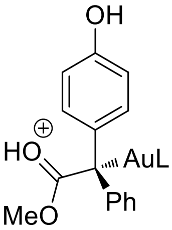

Int-c4

L = $(PhO)_3P$

C	3.82840400	0.95118700	4.60434000
C	3.32491800	0.82450200	3.31304200
C	2.07230500	1.35161900	2.98613300
C	1.33274800	1.98912300	3.99466400
C	1.83853500	2.11563400	5.28056300
C	3.09427500	1.59989200	5.59009100
H	4.80436300	0.53048500	4.83878500
H	3.91888500	0.30771800	2.56394100
H	0.33513800	2.36774100	3.76370800
H	1.24776600	2.61598700	6.04523800
H	3.49464800	1.69752600	6.59695300
C	1.42373900	1.21491200	1.62986700
C	2.20749500	0.85370300	0.48589200
O	1.68284700	0.29334200	-0.57644900
O	3.48867200	1.07074500	0.42663900
C	4.16062800	0.95763900	-0.84252400
H	5.20233400	1.19868300	-0.63211900
H	4.06922700	-0.05777300	-1.23609200
H	3.73135600	1.67795400	-1.54760000
C	-0.11273000	-0.60911800	2.42015400
C	0.06727100	0.54511900	1.63800700
C	-1.01459600	0.98168300	0.86410600
C	-2.22573000	0.29483000	0.85634300
C	-2.38046300	-0.84615800	1.64160400
C	-1.31178600	-1.29451100	2.42717500
H	0.71060100	-0.96461100	3.03938000
H	-0.92300400	1.89182000	0.26550600
H	-3.05588300	0.66129800	0.25222600
H	-1.45169400	-2.18645200	3.03361800
O	-3.52020200	-1.55859900	1.69510800
H	-4.19558700	-1.16037100	1.12686600
Au	1.30024700	3.33551800	1.04819200
P	1.20633000	5.71899200	0.60250400
O	-0.36007200	6.27585200	0.50259200
O	1.96662600	6.57381600	1.81204800

O	1.96634000	6.14907700	-0.81669800
C	2.74104400	7.31159300	-0.96173500
C	2.20578800	8.40304700	-1.63059700
C	4.04896600	7.30337600	-0.49479800
C	3.01027700	9.52186700	-1.82546600
H	1.18266600	8.36594700	-1.99929900
C	4.83945000	8.43058200	-0.69319900
H	4.43040900	6.42425500	0.02160400
C	4.32073200	9.54073700	-1.35602500
H	2.60597800	10.38380700	-2.35213500
H	5.86541500	8.43809300	-0.33123300
H	4.94268500	10.41922100	-1.51373000
C	-0.77671800	7.30693600	-0.35449500
C	-1.05053200	6.99571100	-1.68020000
C	-0.97665400	8.58024700	0.15900600
C	-1.51976200	7.99925500	-2.52121000
H	-0.89296700	5.98071100	-2.04041400
C	-1.44939900	9.57321400	-0.69426400
H	-0.77725700	8.78045500	1.20974000
C	-1.71500200	9.28840000	-2.03077600
H	-1.73753600	7.77021600	-3.56240800
H	-1.61334100	10.57591400	-0.30505500
H	-2.08531800	10.06975700	-2.69106000
C	1.48415500	7.77013900	2.36271100
C	0.47253800	7.70458700	3.31175800
C	2.07449300	8.97158300	1.99659400
C	0.02407500	8.88474500	3.89581400
H	0.04744700	6.73897600	3.58097400
C	1.61813200	10.14330600	2.59339500
H	2.88158700	8.98021300	1.26625200
C	0.59254300	10.10405300	3.53420000
H	-0.76969300	8.84858700	4.63898200
H	2.07218900	11.09333600	2.31946900
H	0.24058000	11.02515500	3.99389300
H	0.73619900	0.09762100	-0.39196300

Structure and coordinates of **Int-c4A**

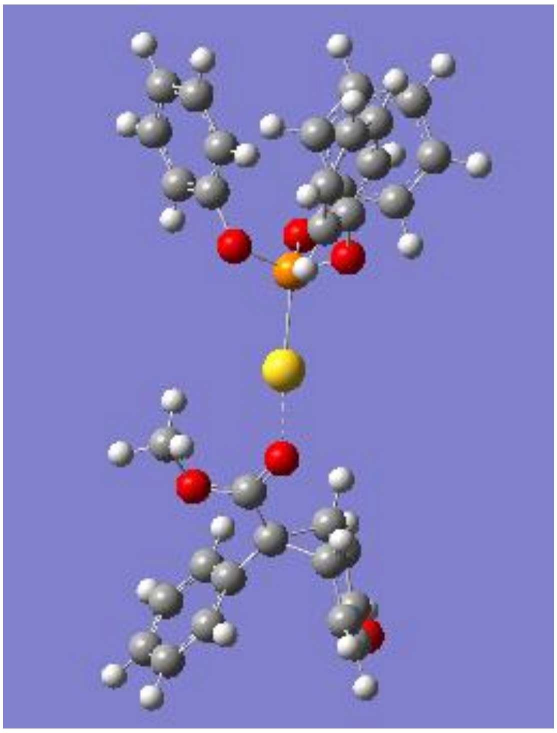

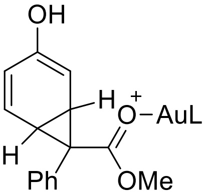

Int-c4A

L = $(PhO)_3P$

C	1.28925500	-2.18687600	4.40528000
C	1.22467300	-1.16235500	3.46810100
C	1.82164000	0.07148100	3.72763700
C	2.49383600	0.26252800	4.93542000
C	2.56055200	-0.75997600	5.87430200
C	1.95352400	-1.98474000	5.61149500
H	0.81920400	-3.14567300	4.19485800
H	0.70635500	-1.31961600	2.52143600
H	2.96932400	1.22342000	5.13565400
H	3.08591500	-0.60136300	6.81427000
H	2.00116500	-2.78480100	6.34781000
C	1.79944600	1.15214700	2.70066800
C	2.89261400	1.19446900	1.74809900
O	2.91234400	2.01923500	0.80338100
O	3.83509300	0.29642000	1.94688200
C	4.90579000	0.16792000	1.01724600
H	5.43452200	-0.74208200	1.30491100
H	4.52551700	0.07447700	-0.00628900
H	5.59287900	1.02249300	1.09167500
C	-0.80783200	1.16727800	2.61288100
C	0.46346100	1.69628300	2.11467200
C	1.23576800	2.58281300	3.03248500
C	0.68140600	2.85838700	4.35289600
C	-0.46483000	2.25926200	4.75754900
C	-1.22441700	1.40747900	3.86841000
H	-1.43140500	0.59849000	1.92553900
H	1.83088000	3.37119000	2.57164200
H	1.19612900	3.57127900	4.99707100
H	-2.16986800	1.02108600	4.24338300
O	-1.04352800	2.43557900	5.96056100
H	-0.50593100	3.03089000	6.50368900
H	0.52801100	1.89440900	1.04559900
Au	4.40231600	3.16517600	-0.17753200

P	5.97179000	4.55717000	-1.26581700
O	5.27144000	5.41309200	-2.49965500
O	6.62296400	5.64462500	-0.19581500
O	7.22840600	3.67425600	-1.89210800
C	8.55985100	4.13486800	-1.90177200
C	9.09907300	4.62269900	-3.08254600
C	9.30743200	4.01849400	-0.73798500
C	10.43479600	5.01491600	-3.08556900
H	8.48414700	4.67948700	-3.97900700
C	10.63975500	4.41781600	-0.75638000
H	8.84152900	3.62810700	0.16499100
C	11.20259800	4.91857000	-1.92840400
H	10.87564500	5.39581300	-4.00444600
H	11.23874700	4.33506000	0.14797100
H	12.24574100	5.22689600	-1.94097100
C	5.92016500	5.69862500	-3.71621300
C	5.92021500	4.72561600	-4.70601500
C	6.46834400	6.95708900	-3.91306100
C	6.50984400	5.02056100	-5.93093700
H	5.46244500	3.75712700	-4.51271500
C	7.05248300	7.23732800	-5.14535900
H	6.42179800	7.70122500	-3.12080000
C	7.08005000	6.27302500	-6.14902100
H	6.51844300	4.26967000	-6.71804700
H	7.48447900	8.22059400	-5.31912800
H	7.53768400	6.50033500	-7.10944000
C	6.86273300	6.99499600	-0.50954500
C	5.79312900	7.87961700	-0.49108900
C	8.16211700	7.40876400	-0.76234300
C	6.03420400	9.22458700	-0.75011000
H	4.79079800	7.50934600	-0.28236800
C	8.38797200	8.75938200	-1.01226800
H	8.97453200	6.68454200	-0.75578700
C	7.32983600	9.66443500	-1.01206300
H	5.20626900	9.93016500	-0.74255600
H	9.40120700	9.10374300	-1.20803300
H	7.51588600	10.71790900	-1.20987700

Structure and coordinates of **Int-c5**

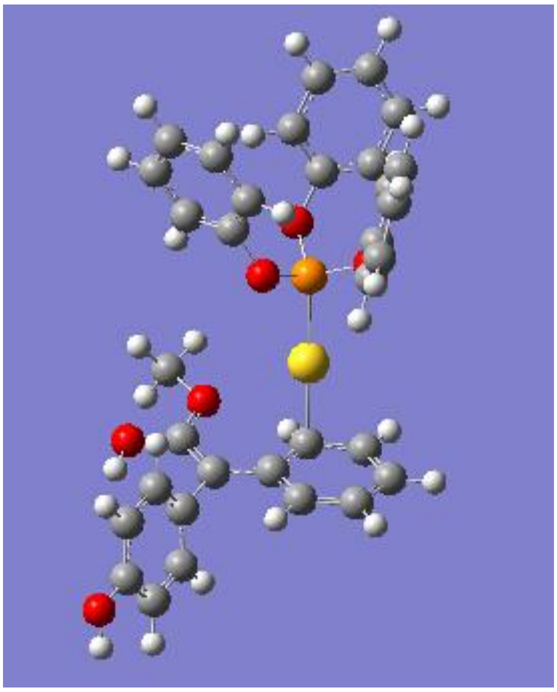

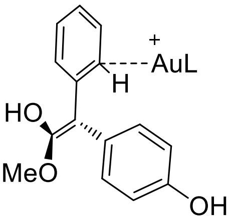

Int-c5
L = (PhO)$_3$P

C	6.22982600	-4.24832300	1.75370200
C	6.27537700	-2.84377500	1.48394600
C	6.48127300	-2.37374600	0.13299000
C	6.50428200	-3.36641300	-0.87699200
C	6.41474900	-4.71367100	-0.58192600
C	6.27966800	-5.17680500	0.73453800
H	6.17058600	-4.56675100	2.79359400
H	6.52295000	-2.17764500	2.31075700
H	6.62693200	-3.05674800	-1.91213500
H	6.45760100	-5.43218300	-1.39891400
H	6.23028600	-6.24139600	0.94718500
C	6.68314100	-0.98877500	-0.23091600
C	6.45699300	0.07361900	0.60943600
O	6.65170100	1.34164800	0.26095100
O	5.98029800	-0.07385800	1.83812000
C	5.81877100	1.08689400	2.66287400
H	5.42987600	0.71094900	3.61056900
H	6.78147200	1.58420700	2.81549400
H	5.11055600	1.78841800	2.21236100
C	6.18572800	-0.10109500	-2.53535600
C	7.10158600	-0.64537300	-1.62106300
C	8.41115600	-0.87564100	-2.05989300
C	8.79465300	-0.58087100	-3.36062200
C	7.86790600	-0.03747300	-4.25404500
C	6.55803900	0.20713600	-3.83458400
H	5.15738700	0.07322000	-2.21434900
H	9.13720200	-1.30067800	-1.36639400
H	9.81877500	-0.76758200	-3.68593800
H	5.85198700	0.62799800	-4.54699800
O	8.17315800	0.27352500	-5.52919100
H	9.10016100	0.06266400	-5.71245900
H	6.99641300	1.36015700	-0.65298700
Au	4.07171100	-2.63641800	1.50011700
P	1.66513000	-2.40203000	1.46702900
O	0.90817000	-3.88438400	1.39965900

O	1.04542400	-1.62324700	2.80220400
O	1.18570200	-1.53760700	0.12479100
C	0.09705500	-0.65333700	0.09905100
C	-1.10116700	-1.06448100	-0.46682500
C	0.28010300	0.63643200	0.58054200
C	-2.15121100	-0.15293600	-0.53213100
H	-1.20004600	-2.07603700	-0.85580000
C	-0.77947100	1.53530600	0.51076200
H	1.24029300	0.91845100	1.00909700
C	-1.99635100	1.13994500	-0.04009500
H	-3.09682600	-0.46036700	-0.97379700
H	-0.65178500	2.54783000	0.88813100
H	-2.82249600	1.84568900	-0.09441100
C	-0.18084000	-4.17105700	0.56411900
C	0.05786100	-4.42573800	-0.78006300
C	-1.44978900	-4.25645000	1.11733000
C	-1.01643800	-4.75779900	-1.59882800
H	1.07190500	-4.35628200	-1.17056700
C	-2.51399700	-4.59505400	0.28667700
H	-1.58838100	-4.07082300	2.18041500
C	-2.30185500	-4.83883800	-1.06774700
H	-0.84603500	-4.95788900	-2.65465400
H	-3.51524000	-4.67093600	0.70563100
H	-3.13944500	-5.10273900	-1.70991600
C	-0.17590100	-1.96752400	3.40769800
C	-0.20127700	-3.03584500	4.29459200
C	-1.29734700	-1.18823400	3.16225000
C	-1.39409500	-3.34047600	4.94222600
H	0.70376000	-3.61443600	4.46992000
C	-2.48234900	-1.50283400	3.82108200
H	-1.23410100	-0.34419900	2.47872900
C	-2.53513800	-2.57760000	4.70371600
H	-1.42853900	-4.17521200	5.63911500
H	-3.36869700	-0.89816600	3.64087900
H	-3.46486300	-2.81672200	5.21563900

Structure and coordinates of **Int-c5A**

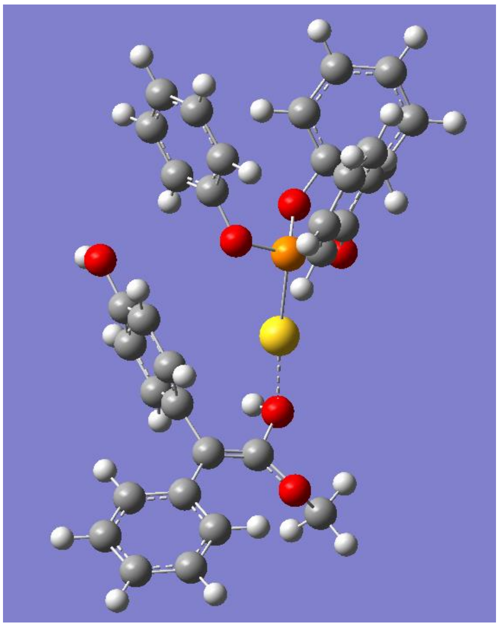

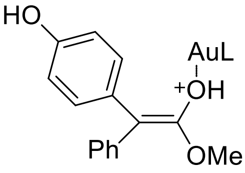

Int-c5A
L = (PhO)$_3$P

C	7.35698800	-4.02075900	2.67804400
C	7.13526400	-2.72466600	2.23212500
C	7.08267400	-2.44473900	0.85872900
C	7.24720800	-3.50410800	-0.04589200
C	7.47436500	-4.79752200	0.40505300
C	7.53081800	-5.06094300	1.76972400
H	7.38825300	-4.22055700	3.74753800
H	6.99229200	-1.92524300	2.95351800
H	7.20459000	-3.30781300	-1.11686600
H	7.60754500	-5.60254500	-0.31509400
H	7.70513600	-6.07454100	2.12529000
C	6.83608100	-1.09108100	0.32677600
C	7.09161500	0.05030600	0.99345000
O	6.77855500	1.31653800	0.41072300
O	7.48722700	0.17874200	2.25230400
C	8.61079500	1.03247500	2.49307200
H	8.85149900	0.92009000	3.55124500
H	9.46599200	0.71382100	1.88321600
H	8.35934400	2.07654800	2.27943000
C	4.82938300	-1.28200400	-1.15548000
C	6.18867600	-0.95886000	-1.00843700
C	6.87772600	-0.47785400	-2.12631700
C	6.23024800	-0.28657100	-3.34335300
C	4.87207700	-0.58499800	-3.46094800
C	4.17281600	-1.09659400	-2.36490100
H	4.28653700	-1.69406800	-0.30308600
H	7.94861300	-0.27628400	-2.05031100
H	6.78461100	0.08416800	-4.20612800
H	3.11937600	-1.34548900	-2.47975300
O	4.17535200	-0.39770200	-4.60476800
H	4.76839500	-0.10703200	-5.31322500
H	7.21058100	1.37690800	-0.45954300
Au	4.59639100	1.51655700	-0.04451700
P	2.30007400	1.93398600	-0.46650100
O	1.56390700	0.60358900	-1.13584600
O	1.46672500	2.31077600	0.91812800
O	2.13463600	3.19323700	-1.53434700
C	1.10084100	4.14627000	-1.46748700

C	0.01637100	4.03095700	-2.32436500
C	1.24363700	5.21351400	-0.59149700
C	-0.96560200	5.01664900	-2.28271800
H	-0.04666200	3.19146600	-3.01395100
C	0.25183000	6.18845400	-0.55972400
H	2.11910900	5.26985600	0.05295000
C	-0.85415100	6.08853200	-1.40119100
H	-1.82216300	4.94479500	-2.94958900
H	0.34751500	7.03007900	0.12290500
H	-1.62645100	6.85420200	-1.37516700
C	0.64399200	0.68239700	-2.19773600
C	1.13485500	0.79226400	-3.49177700
C	-0.71145700	0.57719100	-1.92387800
C	0.22810700	0.80801400	-4.54706200
H	2.20831300	0.85944800	-3.66571900
C	-1.60448800	0.59231900	-2.99132000
H	-1.05003300	0.47674100	-0.89500900
C	-1.13901700	0.71205200	-4.29821000
H	0.59604300	0.89031700	-5.56745900
H	-2.67141900	0.50722300	-2.79638800
H	-1.84352800	0.72195400	-5.12710200
C	0.13543900	1.91947300	1.15909300
C	-0.10300100	0.63304500	1.62402400
C	-0.88106500	2.84897800	0.99995500
C	-1.41040700	0.26231900	1.92103200
H	0.72701300	-0.05967200	1.74919400
C	-2.18297900	2.46437300	1.30684700
H	-0.64881900	3.85457900	0.65523100
C	-2.45015900	1.17517600	1.75960700
H	-1.61396600	-0.74192900	2.28657000
H	-2.99130100	3.18353900	1.19290800
H	-3.47077600	0.88372100	1.99813000

Structure and coordinates of **Int-c6**

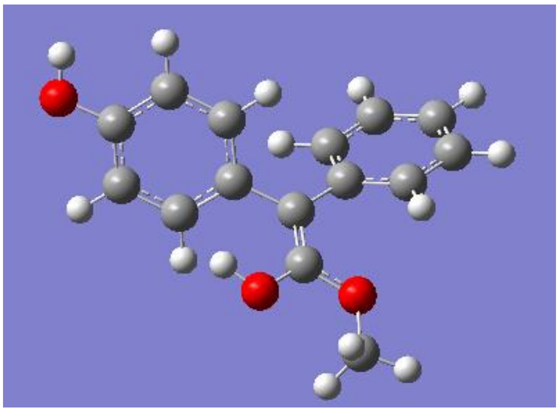

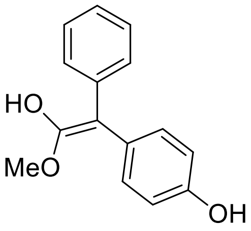

Int-c6

C	2.57205400	-3.63580300	0.98296100
C	2.60864400	-2.26890900	1.22599600
C	2.73217600	-1.35003000	0.17292600
C	2.83274800	-1.86646300	-1.12838400
C	2.79865600	-3.23311300	-1.36970600
C	2.66601700	-4.12944900	-0.31420300
H	2.47898200	-4.32462200	1.82193200
H	2.54590800	-1.90363600	2.24761700
H	2.93243600	-1.17503600	-1.96474300
H	2.87260200	-3.59982800	-2.39279000
H	2.63958300	-5.20191500	-0.49994400
C	2.79387400	0.10965700	0.37537800
C	2.14951000	0.75922800	1.37973800
O	2.24039200	2.08360100	1.61793500
O	1.35214000	0.13216800	2.25218200
C	0.65829000	0.90481000	3.21700900
H	0.06807900	0.18799900	3.79247900
H	1.35031900	1.43440300	3.88179600
H	-0.00800100	1.63478200	2.74245000
C	3.08700600	1.99110600	-1.29208100
C	3.61837500	0.91817100	-0.55922500
C	4.97364700	0.61825000	-0.75269200
C	5.76326500	1.35702700	-1.62026500
C	5.21517300	2.43335000	-2.32004500
C	3.87032000	2.75047200	-2.15261900
H	2.02398900	2.21878300	-1.19916400
H	5.40839100	-0.22010000	-0.20898900
H	6.81680200	1.10402500	-1.75320100
H	3.45161400	3.57881400	-2.72038500
O	5.94132900	3.19669100	-3.17651700
H	6.84632700	2.85743600	-3.21226400
H	2.87859900	2.45528500	0.98408400

Structure and coordinates of **TS-c6-2w**

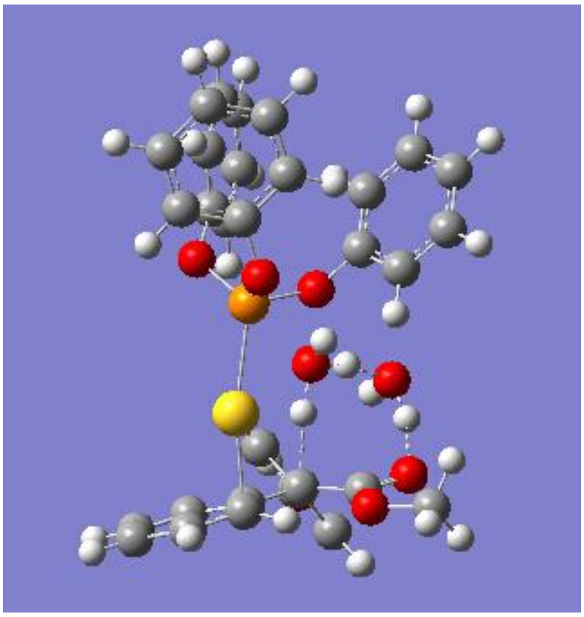

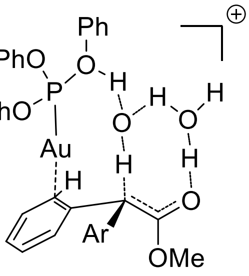

TS-c6-2w

C	-3.56151200	-4.57901000	-8.61438900
C	-3.69928100	-3.42957400	-7.86468200
C	-4.95805000	-2.96209000	-7.40103900
C	-6.10059800	-3.75744000	-7.83313500
C	-5.90537000	-4.97353300	-8.57500100
C	-4.66006800	-5.37018100	-8.99316800
H	-2.55818700	-4.89079600	-8.90158000
H	-2.80101600	-2.89617100	-7.56827600
H	-7.01255000	-3.68791800	-7.24124600
H	-6.78468900	-5.58219400	-8.78389300
H	-4.52003600	-6.28160700	-9.56791200
C	-5.03759700	-1.74630200	-6.60681600
C	-6.20534800	-1.34360000	-5.82895500
O	-6.27163100	-0.30209300	-5.17100100
O	-7.29361500	-2.13097500	-5.92458300
C	-8.44020600	-1.69560000	-5.19194900
H	-9.20059900	-2.46246500	-5.35185100
H	-8.20587600	-1.60708100	-4.12725800
H	-8.79124000	-0.72494900	-5.55791700
C	-2.84927000	-0.51522500	-7.01064000
C	-3.75916600	-1.11091800	-6.12899500
C	-3.44888500	-1.05339700	-4.76082000
C	-2.31131800	-0.41754000	-4.29385500
C	-1.41974500	0.17333900	-5.19353100
C	-1.69373700	0.11981400	-6.56039200
H	-3.04085900	-0.54050800	-8.08483500
H	-4.13202400	-1.50297200	-4.04221700
H	-2.08128300	-0.37629400	-3.23139500
H	-1.00010700	0.56475300	-7.27541700
H	-5.38368300	1.14855600	-5.59516500
O	-5.44018000	0.38659500	-8.20641900
H	-5.37137400	-0.56770800	-7.67756400
H	-6.32721600	0.52347700	-8.61093500
Au	-6.72404000	-2.53148200	-9.52254400
P	-7.44852700	-0.76774900	-11.02169700
O	-6.35715900	-0.29416300	-12.18267400
O	-8.92757700	-0.93380500	-11.74970800
O	-7.60566100	0.56654800	-9.97987600

C	-8.65736100	1.49936400	-10.06610400
C	-8.40647400	2.74805500	-10.61503600
C	-9.89292400	1.15767300	-9.53266900
C	-9.44079000	3.67929000	-10.63948700
H	-7.41861100	2.98199400	-11.00652100
C	-10.91734500	2.09774400	-9.56882800
H	-10.04349000	0.16645600	-9.10703700
C	-10.69306100	3.35576000	-10.12424300
H	-9.26262800	4.66498900	-11.06393000
H	-11.89301500	1.84588900	-9.15890100
H	-11.49646600	4.08873600	-10.14847800
C	-6.02970100	1.04868400	-12.43573000
C	-5.09297700	1.66185100	-11.61400100
C	-6.59354700	1.69404500	-13.52572000
C	-4.72439700	2.97528500	-11.88603100
H	-4.66731200	1.10794600	-10.77825200
C	-6.21095400	3.00688600	-13.78707400
H	-7.30551200	1.16852800	-14.15864900
C	-5.28542400	3.64910000	-12.96883000
H	-3.99160300	3.47130000	-11.25300700
H	-6.63969000	3.52726800	-14.64085400
H	-4.99235600	4.67497000	-13.18147700
C	-9.26218000	-0.40457900	-13.01129200
C	-8.81951300	-1.06187500	-14.15120400
C	-10.08492200	0.71031000	-13.07240100
C	-9.20230000	-0.57037300	-15.39525600
H	-8.18901100	-1.94443000	-14.06183400
C	-10.46429000	1.18494800	-14.32451800
H	-10.43039900	1.18257200	-12.15519300
C	-10.02069100	0.55325000	-15.48310300
H	-8.86405700	-1.07437600	-16.29805000
H	-11.11481100	2.05450800	-14.38993000
H	-10.32330900	0.92997800	-16.45773400
O	-0.32680000	0.77515200	-4.67877100
H	0.23647800	1.10952200	-5.39127400
O	-4.97893400	1.79030600	-6.23971700
H	-5.23493400	1.11401000	-7.41010100
H	-4.02301500	1.75370700	-6.06584600

Structure and coordinates of **TS-c6-2p**

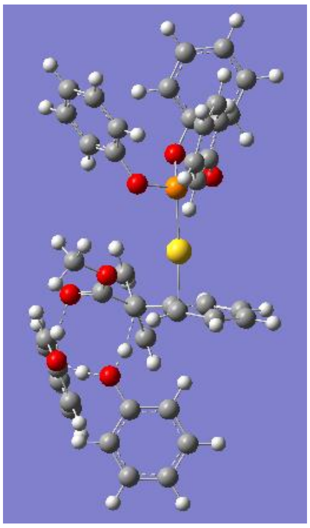

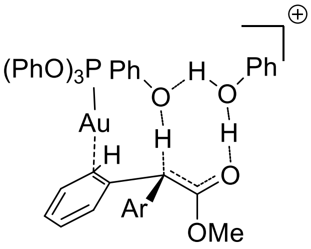

TS-c6-2p

C	6.44570200	-4.80807500	-8.05462000
C	6.01578400	-3.62944700	-7.47265700
C	6.51871000	-3.17750600	-6.22514300
C	7.58442100	-3.97107400	-5.65610400
C	7.96910700	-5.20996800	-6.26181900
C	7.41834900	-5.62350500	-7.45678100
H	6.00951500	-5.10906200	-9.00615100
H	7.82143300	-3.83400100	-4.59825100
H	8.70358900	-5.82537800	-5.74382600
H	7.72660300	-6.55641400	-7.92119900
C	5.88832800	-2.07342100	-5.52233700
C	6.60727800	-1.20213600	-4.61663600
O	6.08890200	-0.31468100	-3.92267900
O	7.93887300	-1.39823100	-4.53593100
C	8.66461900	-0.55826200	-3.63798000
H	9.72031000	-0.78861200	-3.81232700
H	8.46167300	0.49669800	-3.84540600
H	8.39105400	-0.77550500	-2.60022800
C	3.62447400	0.38396700	-7.34997600
C	2.41208800	-0.30624500	-7.37021900
C	2.32602500	-1.56776100	-6.78265700
C	3.44056500	-2.11610200	-6.16809500
C	4.67165800	-1.44609300	-6.13440600
C	4.73550200	-0.18890900	-6.74491800
H	3.69997600	1.36862900	-7.81423700
H	1.37157300	-2.08943900	-6.80371300
H	3.34919900	-3.10378000	-5.70902500
H	5.67800100	0.35895000	-6.74287000
O	1.28793400	0.19640600	-7.93125100
H	4.66715600	-0.60490400	-3.21035500
O	3.91556500	-1.12900000	-2.77258700
O	4.82910400	-3.25914600	-3.34312100
H	4.26281400	-2.26809800	-2.97717800
H	5.24051200	-2.96191000	-4.28737800
H	1.48136000	1.05332900	-8.33727700
C	4.15058700	-4.47179700	-3.29556300
C	3.44054100	-4.76415300	-2.13795800
C	4.21333400	-5.35976900	-4.36165500
C	2.76217500	-5.97519800	-2.05508000
H	3.44029400	-4.05497900	-1.31027300
C	3.53079900	-6.56915200	-4.25880600

H	4.78417400	-5.11352500	-5.25817300
C	2.80437700	-6.87858300	-3.11372000
H	2.20371000	-6.21555100	-1.15283700
H	3.57028100	-7.27357700	-5.08709200
H	2.27451400	-7.82575400	-3.04377600
C	2.66426800	-0.75796000	-3.28305100
C	2.51040100	0.49979100	-3.84315900
C	1.61873500	-1.66212400	-3.18704400
C	1.25885200	0.85587600	-4.33245400
H	3.35807400	1.18183100	-3.89669000
C	0.37311800	-1.28772200	-3.68149900
H	1.77644400	-2.64266800	-2.73729200
C	0.19136000	-0.03415200	-4.25738000
H	1.12055700	1.83847400	-4.77971600
H	-0.45916700	-1.98532600	-3.61449300
H	-0.78319500	0.25137600	-4.64658000
H	5.24009500	-3.04725700	-7.96419500
Au	9.28474600	-2.85829300	-6.53389100
P	11.26871900	-1.68317200	-7.25179100
O	11.01777000	-0.42355500	-8.31360600
O	11.98963000	-1.03864400	-5.88697400
O	12.40183200	-2.66507200	-7.97872300
C	13.76619400	-2.66310600	-7.66027000
C	14.66299300	-2.09458700	-8.55337100
C	14.18508700	-3.29484300	-6.49656000
C	16.02163300	-2.15213200	-8.25741200
H	14.29423500	-1.63006600	-9.46572200
C	15.54567000	-3.33858400	-6.21089600
H	13.44836900	-3.73805100	-5.82828500
C	16.46360200	-2.76503500	-7.08809600
H	16.73817500	-1.71602600	-8.95042400
H	15.88876500	-3.82756000	-5.30150500
H	17.52727600	-2.80443900	-6.86303600
C	11.92949700	-0.06741100	-9.32116800
C	11.93683500	-0.79716100	-10.50298700
C	12.74035400	1.04375300	-9.13877700
C	12.79525600	-0.40800800	-11.52599200
H	11.27910300	-1.65783900	-10.60819600
C	13.59052500	1.42352900	-10.17312200
H	12.69349300	1.60434900	-8.20754400
C	13.62408000	0.69949900	-11.36147400
H	12.81140200	-0.97186100	-12.45638100
H	14.22866600	2.29547900	-10.04583600
H	14.29088200	1.00318900	-12.16569400
C	12.65818600	0.19159400	-5.85000900
C	11.90423100	1.35235800	-5.72793800
C	14.04566500	0.21360200	-5.85596700
C	12.56304300	2.57384900	-5.63014100
H	10.81680500	1.28755100	-5.72396400
C	14.69044300	1.44268000	-5.75366200
H	14.60337400	-0.71789000	-5.92940000
C	13.95521000	2.61991900	-5.64728900
H	11.98562300	3.49157100	-5.53922900
H	15.77783800	1.47632300	-5.75504300
H	14.46727100	3.57654000	-5.56743900

Structure and coordinates of **TS-c6-1w**

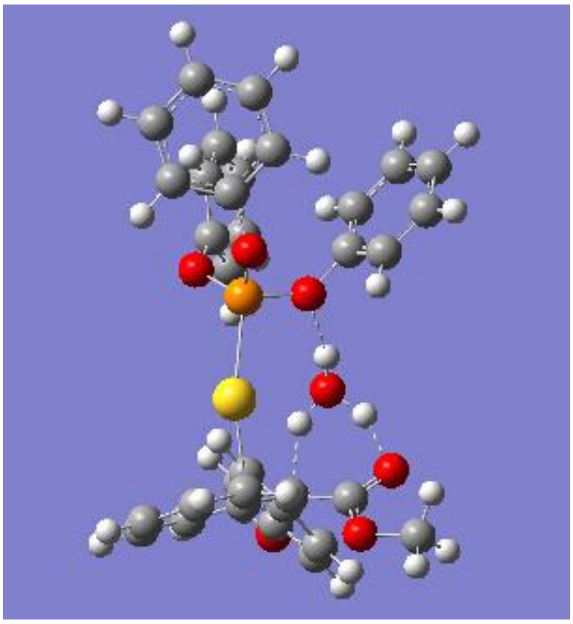

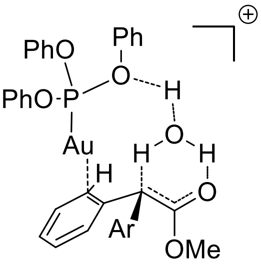

TS-c6-1w
L = (PhO)$_3$P

C	-3.26136963	-4.32352866	-8.84679204
C	-3.39861353	-3.11374618	-8.20477111
C	-4.64050388	-2.70560446	-7.64941195
C	-5.77881171	-3.58904187	-7.88138711
C	-5.56311711	-4.86549336	-8.51381141
C	-4.33835636	-5.22165835	-9.01176744
H	-2.27842345	-4.60338810	-9.22330024
H	-2.53577437	-2.46584872	-8.07563072
H	-6.60973303	-3.52608409	-7.17542317
H	-6.41064049	-5.54705744	-8.57678597
H	-4.18772367	-6.17985465	-9.50141386
C	-4.73398824	-1.42956527	-7.01041936
C	-5.80701021	-0.94550681	-6.15269994
O	-5.95828620	0.25687869	-5.89861086
O	-6.67694163	-1.86001388	-5.71914096
C	-7.78276584	-1.36094660	-4.95805142
H	-8.37662030	-2.23699229	-4.69337363
H	-7.42553006	-0.85349133	-4.05762778
H	-8.37525937	-0.65768967	-5.55291506
C	-2.56053769	0.03068636	-6.38443828
C	-3.41746369	-1.03243164	-6.11674828
C	-3.25822869	-1.81125664	-4.97590328
C	-2.22375769	-1.50768164	-4.09251828
C	-1.36143569	-0.44805264	-4.34461128
C	-1.53424769	0.31867436	-5.49556028
H	-2.71866269	0.61708336	-7.28739628
H	-3.92539269	-2.64822264	-4.77448528
H	-2.09551869	-2.11466464	-3.19814428
H	-0.86508569	1.15164436	-5.70275428
H	-5.56528359	0.99864615	-7.34023804
O	-5.36395111	0.78952567	-8.32433176
H	-5.13751641	-0.24143897	-8.14531197
H	-6.19671779	0.84311527	-8.86510389
Au	-6.62983604	-2.47505325	-9.54815022
P	-7.46647014	-0.76537954	-11.05664597
O	-6.45606158	-0.39874532	-12.32050481
O	-9.00519388	-0.91481915	-11.64521644
O	-7.48703549	0.64091643	-10.08153631

C	-8.51130006	1.60940988	-10.14468707
C	-8.27873289	2.79601770	-10.82275169
C	-9.69264377	1.36174031	-9.45887635
C	-9.28009130	3.76302551	-10.82045142
H	-7.33162240	2.95652885	-11.33365274
C	-10.68498315	2.33651703	-9.46998221
H	-9.82659260	0.41692273	-8.93389265
C	-10.47992192	3.53404494	-10.15222184
H	-9.11602813	4.70200091	-11.34450443
H	-11.61901625	2.16087792	-8.94085536
H	-11.25687375	4.29535184	-10.15668416
C	-6.08396721	0.90773667	-12.68321171
C	-5.03291872	1.50357687	-11.99877086
C	-6.72061959	1.52756064	-13.74746843
C	-4.62189743	2.77603077	-12.38186357
H	-4.54149208	0.96589604	-11.18854599
C	-6.29316392	2.79805735	-14.12356759
H	-7.52276096	1.01463338	-14.27371125
C	-5.25376370	3.42440184	-13.44113070
H	-3.79881558	3.25683643	-11.85767951
H	-6.77584459	3.29604645	-14.96171864
H	-4.92549293	4.41650700	-13.74328355
C	-9.43819659	-0.42910433	-12.89673435
C	-9.11308330	-1.14399654	-14.04090497
C	-10.23642928	0.70448372	-12.93145445
C	-9.59155914	-0.69091924	-15.26645970
H	-8.50109202	-2.04106075	-13.96764335
C	-10.71028738	1.14119149	-14.16492756
H	-10.49274595	1.22010247	-12.00835810
C	-10.38489199	0.45224794	-15.33011753
H	-9.34784493	-1.24089676	-16.17277654
H	-11.34314401	2.02490439	-14.21113988
H	-10.76016057	0.79874895	-16.29051095
O	-0.30440706	-0.14675666	-3.42983742
H	0.48458482	-0.63283097	-3.68048376

Structure and coordinates of **Int-c7**

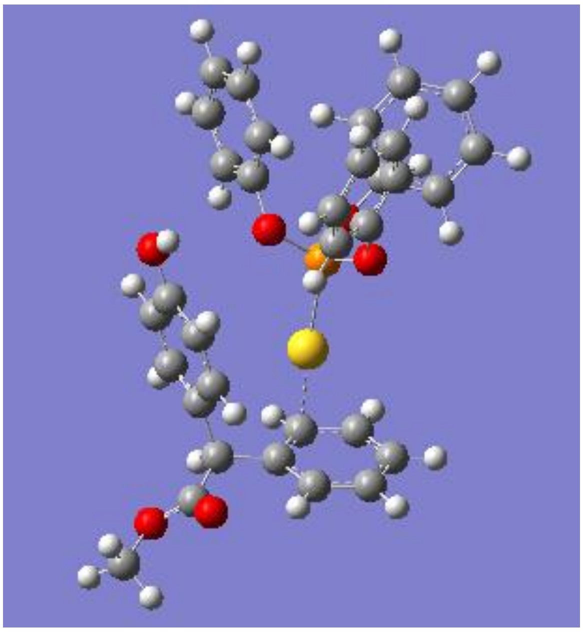

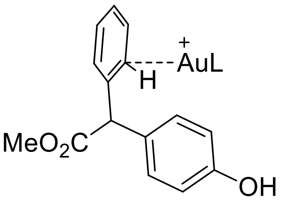

Int-c7
L = (PhO)$_3$P

C	-8.26240000	-4.79690500	-7.42127600
C	-6.95697300	-4.45093400	-7.06278600
C	-6.69956000	-3.25686500	-6.39541700
C	-7.79554300	-2.38952600	-6.11012300
C	-9.11411400	-2.76047200	-6.47947500
C	-9.33947400	-3.96096700	-7.14925300
H	-8.43403600	-5.74655400	-7.92464700
H	-6.13216400	-5.12489400	-7.27868600
H	-7.65573200	-1.57392100	-5.39358000
H	-9.94640000	-2.12236800	-6.18694500
H	-10.34947200	-4.24951900	-7.42939900
C	-5.31599000	-2.77907600	-5.99621700
C	-4.34237600	-3.89839200	-5.66363400
O	-4.14262600	-4.87413800	-6.35066600
O	-3.69895200	-3.62914600	-4.53243300
C	-2.69840500	-4.58129400	-4.15014500
H	-2.27055500	-4.20299700	-3.22113100
H	-3.15262100	-5.56482800	-3.99499200
H	-1.93242800	-4.65906800	-4.92783000
C	-4.67233700	-0.51104700	-6.91719500
C	-4.72528900	-1.89738600	-7.08968400
C	-4.28630000	-2.44057400	-8.30156800
C	-3.81990300	-1.61936500	-9.31738200
C	-3.79359600	-0.23299600	-9.14438300
C	-4.21606400	0.31980800	-7.93426000
H	-4.99061600	-0.07069500	-5.97065400
H	-4.29481000	-3.52092500	-8.44178000
H	-3.45468500	-2.03689800	-10.25423400
H	-4.19236400	1.40126600	-7.79123500
H	-5.41826000	-2.16280600	-5.09223000
Au	-7.63068800	-1.17096200	-8.02376800
P	-7.58245700	0.27816000	-9.95478200
O	-7.58949500	-0.53305400	-11.40479000
O	-8.89145600	1.30736100	-9.95063900
O	-6.21130400	1.22314700	-9.96414300
C	-6.15234500	2.58855800	-10.25236400
C	-5.58234100	2.99000300	-11.45367800
C	-6.57439400	3.49991200	-9.29171100

C	-5.45564500	4.35383200	-11.70243100
H	-5.24047900	2.23701700	-12.16339500
C	-6.44793800	4.85925900	-9.56000900
H	-7.00084700	3.13725200	-8.35705400
C	-5.89388300	5.28603400	-10.76508400
H	-5.01205400	4.68788400	-12.63802800
H	-6.78156400	5.58629700	-8.82261100
H	-5.79536100	6.34992100	-10.96959300
C	-6.85093600	-0.09572900	-12.52156600
C	-5.49813700	-0.39781000	-12.57554200
C	-7.50275200	0.56623000	-13.55183700
C	-4.76681500	-0.00058900	-13.68990600
H	-5.03160200	-0.92129400	-11.74384100
C	-6.76006900	0.94770400	-14.66576200
H	-8.56982100	0.76783600	-13.48255600
C	-5.39653500	0.67357900	-14.73397200
H	-3.70236700	-0.22145100	-13.73703200
H	-7.25627700	1.46309900	-15.48555300
H	-4.82380100	0.97783500	-15.60761900
C	-9.54050000	1.74067800	-11.12005600
C	-10.50194300	0.91434700	-11.68576400
C	-9.25289500	2.99719700	-11.63285500
C	-11.18436700	1.35691200	-12.81411500
H	-10.70214200	-0.06037300	-11.24450900
C	-9.94715300	3.42787500	-12.75977300
H	-8.50623400	3.62379800	-11.14914400
C	-10.90548300	2.61088600	-13.35355200
H	-11.93864600	0.71922300	-13.27016100
H	-9.73700300	4.41200600	-13.17320900
H	-11.44354700	2.95422000	-14.23448400
O	-3.36141600	0.51212100	-10.18367800
H	-3.46069700	1.45387300	-9.97845000

Structure and coordinates of **TS-c7-2w**

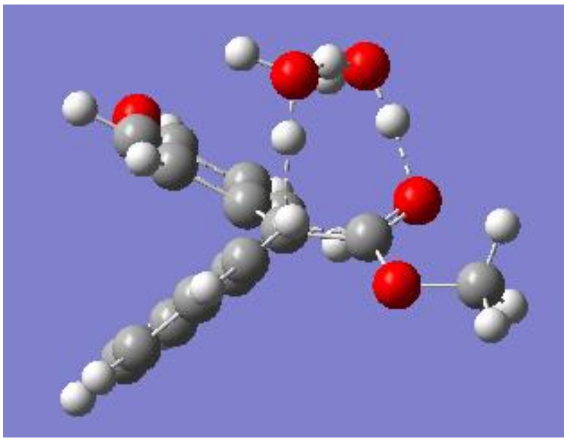

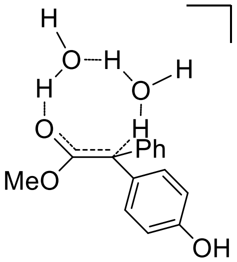

TS-c7-2w

C	5.57020800	-4.68418700	-8.67403400
C	5.00861000	-3.76270400	-7.79791300
C	5.68413400	-3.35059500	-6.64168600
C	6.94868900	-3.90716500	-6.40052200
C	7.51859000	-4.81753100	-7.28024400
C	6.83040400	-5.21625300	-8.42271300
H	5.01938600	-4.98175600	-9.56573100
H	4.02332500	-3.34676100	-8.00871000
H	7.49370800	-3.60844700	-5.50419600
H	8.50436000	-5.22896500	-7.06572000
H	7.27146700	-5.93711300	-9.10928000
C	5.06466400	-2.40009200	-5.67747400
C	5.74295000	-1.20121200	-5.31642000
O	5.36292100	-0.36081900	-4.45839900
O	6.93503100	-1.00516200	-5.91114300
C	7.70309400	0.08596300	-5.43154000
H	8.63611300	0.06211700	-5.99962000
H	7.18581200	1.03754600	-5.59671700
H	7.91081100	-0.01402800	-4.35977100
C	1.39733200	-1.41479300	-5.20307900
C	0.80029800	-2.65891800	-5.01518800
C	1.59022500	-3.80625600	-5.08160900
C	2.95143500	-3.69454800	-5.31503500
C	3.58970800	-2.45322200	-5.50454600
C	2.76404300	-1.31980500	-5.46045100
H	0.78751800	-0.50933800	-5.17491800
H	1.11632400	-4.77662100	-4.94507200
H	3.55187600	-4.60466600	-5.37763600
H	3.20442200	-0.33524400	-5.60682400
O	-0.52691500	-2.81741100	-4.76393900
H	4.53861400	-0.88372800	-3.33319500
O	4.04710300	-1.47197500	-2.61454900
O	5.38633300	-3.39472900	-3.16998800
H	4.63999500	-2.47510200	-2.75720600
H	5.39874700	-3.10706000	-4.20138100
H	3.16266300	-1.60482600	-3.00415100
H	4.94941200	-4.25849100	-3.13107900
H	-0.95922300	-1.95299600	-4.79704700

Structure and coordinates of **TS-c7-1w**

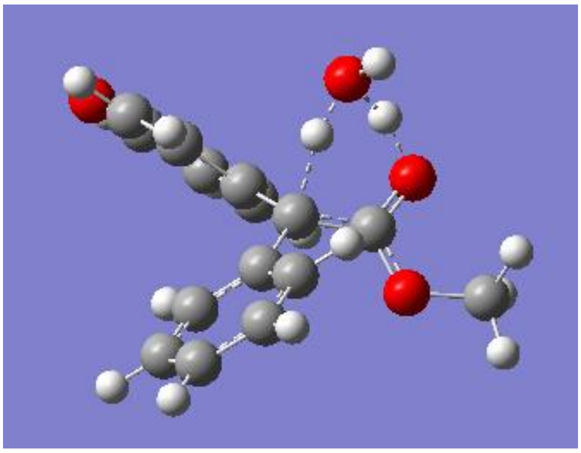

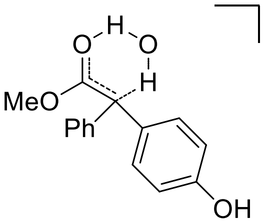

TS-c7-1w

C	1.16317000	-2.87177400	3.86023100
C	1.33743000	-1.64394800	3.23744000
C	1.84995700	-1.54854900	1.93686000
C	2.16448900	-2.74075800	1.27624300
C	1.98123300	-3.97400100	1.89237900
C	1.48188200	-4.04818500	3.18767400
H	0.76346100	-2.91193900	4.87282100
H	1.07085800	-0.72853200	3.76891800
H	2.56070300	-2.69740800	0.26237000
H	2.23704500	-4.88535400	1.35320500
H	1.33720200	-5.01443700	3.66822100
C	2.01203500	-0.21745300	1.28593700
C	2.82340200	0.75906200	1.94241400
O	2.76374600	2.00393500	1.66611500
O	3.59487000	0.37780000	2.95514300
C	4.21520300	1.40482000	3.72142100
H	4.75436300	0.89000300	4.51903200
H	4.90922100	1.98530800	3.10507800
H	3.46893600	2.08566700	4.14556900
C	0.90836900	-0.65949300	-0.92668000
C	2.01883800	-0.18200600	-0.21470200
C	3.09970500	0.29440500	-0.95875300
C	3.07522500	0.30826200	-2.34933500
C	1.96422600	-0.17875100	-3.03055600
C	0.87628600	-0.67169600	-2.31017100
H	0.05045000	-1.03690800	-0.36856800
H	3.98475500	0.66908900	-0.44567700
H	3.93357100	0.68746100	-2.90715800
H	0.01235700	-1.04562500	-2.85589100
O	1.88347200	-0.20078000	-4.38817700
H	2.69651800	0.17312700	-4.75442700
H	1.58863400	2.15840100	1.28207900
H	0.83210600	0.77075100	1.33372100
O	0.45084400	1.84568400	1.13451800
H	-0.04317900	2.14305300	1.91359900

Structure and coordinates of **pro-c8**

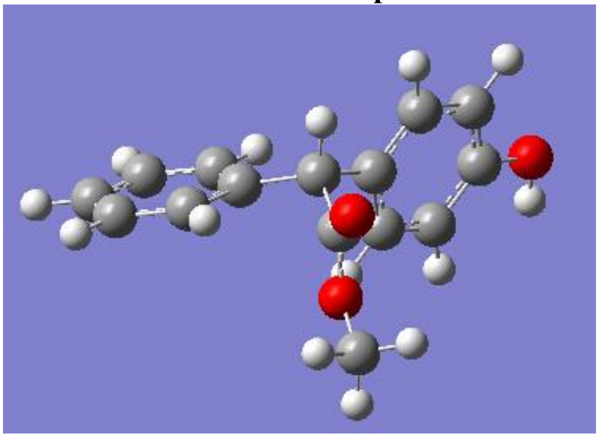

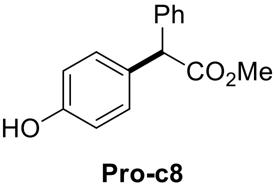

Pro-c8

C	-0.14328100	3.86915800	2.74796600
C	0.25296600	3.19791000	1.59857800
C	0.82235200	1.92389100	1.67345700
C	0.97127500	1.33189800	2.92645100
C	0.57228200	2.00209300	4.07881200
C	0.01731000	3.27327900	3.99519200
H	-0.58181400	4.86256000	2.66740000
H	0.10868300	3.66843500	0.62438300
H	1.40459800	0.33584700	3.00198500
H	0.69772600	1.52385700	5.04911800
H	-0.29092000	3.79869900	4.89746300
C	1.18463800	1.18192400	0.39334300
C	1.66148200	2.15810600	-0.67044500
O	1.06195200	2.41145200	-1.68709900
O	2.82407500	2.73928900	-0.33480900
C	3.30808200	3.69307700	-1.27395200
H	4.24957000	4.06732000	-0.86733800
H	3.46911700	3.22585000	-2.25134000
H	2.58909600	4.51140400	-1.39257600
C	1.79885100	-1.24380700	0.11684800
C	2.15389000	0.03151200	0.55927900
C	3.41542000	0.20007900	1.13520000
C	4.29043300	-0.86926600	1.26369800
C	3.91716300	-2.13564500	0.81274800
C	2.66394000	-2.32203000	0.23599700
H	0.81884300	-1.39678100	-0.33608000
H	3.71496500	1.18558600	1.48831200
H	5.27211900	-0.72196800	1.71760500
H	2.38717800	-3.31559900	-0.10950700
O	4.73141200	-3.21727000	0.91203400
H	5.56522900	-2.94871900	1.32201700
H	0.25381600	0.77496200	-0.03048300

Section 10: Cartesian coordinates of structures in Figure 4

Structure and coordinates of **TS-o1**

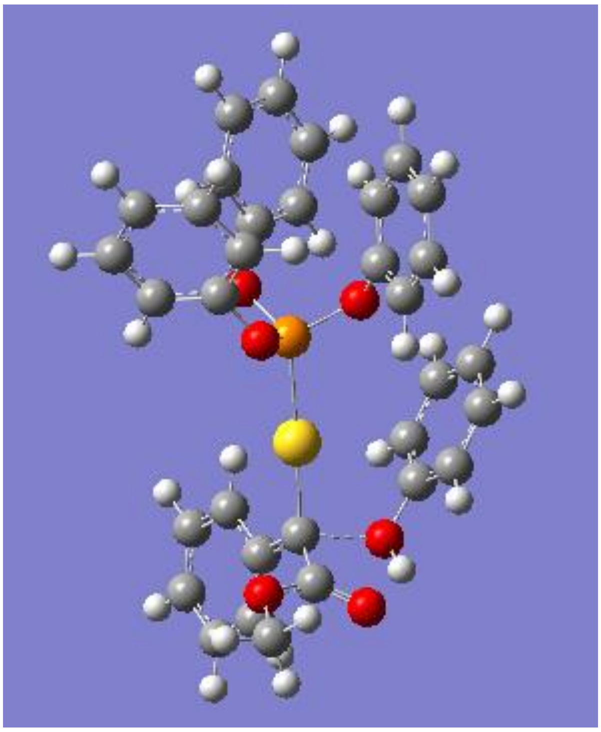

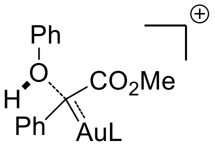

TS-o1

L = (PhO)$_3$P

C	1.90368600	3.29754800	4.48668200
C	2.22423800	2.53335600	3.37929100
C	1.23035000	2.17782300	2.44432800
C	-0.09348500	2.60024700	2.66556400
C	-0.41144800	3.36989400	3.77272700
C	0.58693900	3.71783800	4.68090300
H	2.67464800	3.56972700	5.20367300
H	3.25122000	2.19555400	3.23593700
H	-0.86535200	2.32159600	1.94693800
H	-1.43454400	3.70245700	3.93140900
H	0.33738500	4.32223200	5.55089900
C	1.55418700	1.36653500	1.29363100
C	2.98895700	1.32417600	0.85991800
O	3.72984600	0.36093000	0.96684000
O	3.32259600	2.47178200	0.30256800
C	4.65451600	2.54947100	-0.23554000
H	4.73612400	3.54736600	-0.66595300
H	5.39015900	2.40763700	0.56155700
H	4.79334200	1.78113100	-1.00165900
C	-0.56588400	-1.37325000	1.99731900
C	0.72633600	-1.30912600	1.49469700
C	1.12896800	-2.04633800	0.38624000
C	0.20045700	-2.87909300	-0.22991000
C	-1.10387600	-2.95556000	0.25299200
C	-1.48368800	-2.20520100	1.36356300
H	-0.82901700	-0.77882600	2.87056300
H	2.14940700	-1.96187100	0.01084900
H	0.49757700	-3.46771000	-1.09545100
H	-2.49835800	-2.27670900	1.75008400
O	1.59549900	-0.40981100	2.09936600
H	2.53590400	-0.60052100	1.83912400
H	-1.82493700	-3.60669100	-0.23588600
Au	0.24364500	1.11926700	-0.31726500
P	-1.22749600	0.92200200	-2.25251200

O	-0.54938300	1.64229700	-3.59505600
O	-1.55369200	-0.66134600	-2.65587700
O	-2.70967200	1.65115600	-2.01740200
C	-3.92047900	1.11650000	-2.47804100
C	-4.52097000	1.66219500	-3.60296700
C	-4.51834900	0.09889700	-1.74540400
C	-5.75323800	1.15770300	-4.00895400
H	-4.03036300	2.47323000	-4.13752400
C	-5.74727500	-0.39835800	-2.16536000
H	-4.01325200	-0.29387100	-0.86430400
C	-6.36386500	0.12798400	-3.29847900
H	-6.23795700	1.57813700	-4.88766700
H	-6.22688100	-1.19678900	-1.60287200
H	-7.32685600	-0.26019800	-3.62343700
C	-1.27971100	2.34040300	-4.56892500
C	-1.63813100	3.65770200	-4.31242600
C	-1.55685900	1.72462400	-5.78081200
C	-2.31235400	4.37212700	-5.29724600
H	-1.39096300	4.10657100	-3.35200500
C	-2.22863400	2.45351700	-6.75787200
H	-1.23864900	0.69809800	-5.95131500
C	-2.61117400	3.77042600	-6.51756300
H	-2.59971300	5.40469500	-5.11004600
H	-2.44926200	1.98561700	-7.71508100
H	-3.13520600	4.33321100	-7.28716900
C	-1.61132300	-1.15528600	-3.96517400
C	-0.42136900	-1.45836900	-4.61439400
C	-2.84799400	-1.39759800	-4.54701400
C	-0.47451300	-2.00337000	-5.89323700
H	0.52780700	-1.26196900	-4.11778800
C	-2.88515600	-1.94815500	-5.82478100
H	-3.75945600	-1.16586900	-3.99945000
C	-1.70484400	-2.24463800	-6.50062500
H	0.44984700	-2.24286900	-6.41486800
H	-3.84752300	-2.14532100	-6.29269000
H	-1.74300000	-2.67318800	-7.49984200

Structure and coordinates of **Int-o2**

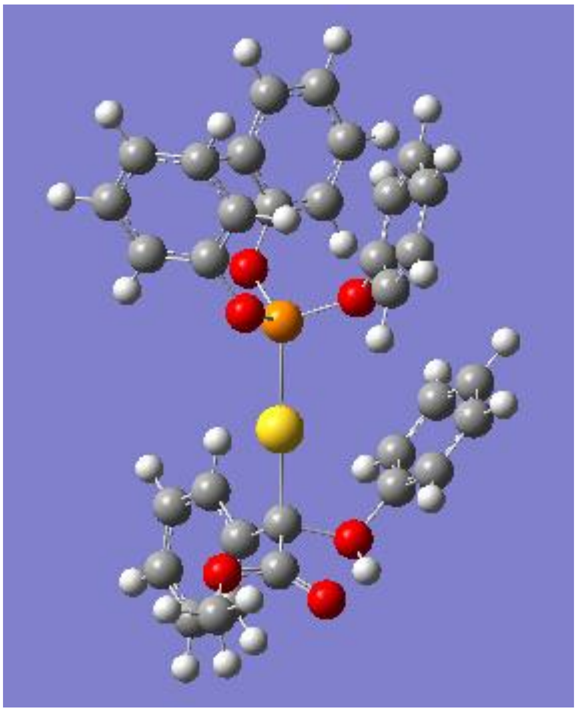

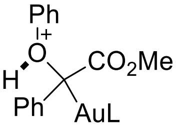

Int-o2

L = (PhO)$_3$P

C	1.83733500	3.13335700	4.51778200
C	2.15804700	2.30940300	3.44976400
C	1.22321400	2.06104100	2.43506000
C	-0.04140800	2.64957000	2.52024100
C	-0.36255800	3.47993200	3.58814800
C	0.57553700	3.72167600	4.58588200
H	2.57139100	3.31998400	5.29870000
H	3.14562800	1.84602400	3.40311100
H	-0.77908400	2.45638800	1.73936200
H	-1.34771200	3.93828400	3.64113000
H	0.32416600	4.37055100	5.42240600
C	1.58130200	1.14572400	1.31745800
C	3.00265600	1.23635700	0.84746800
O	3.75268800	0.26355800	0.89053800
O	3.32678300	2.41777900	0.38498300
C	4.66333400	2.55826500	-0.12851000
H	4.72593400	3.58225500	-0.49583400
H	5.39109400	2.38643300	0.66974100
H	4.82948400	1.84189700	-0.93812000
C	-0.54884300	-1.26223800	2.12159900
C	0.67512800	-1.25085100	1.47988300
C	0.99063900	-2.06069100	0.40181100
C	0.01073500	-2.93588400	-0.05704000
C	-1.23864700	-2.97048300	0.55841600
C	-1.51722400	-2.14040500	1.64169600
H	-0.72926900	-0.60243900	2.96756700
H	1.96920300	-1.99627700	-0.07452900
H	0.22520200	-3.58652000	-0.90157100
H	-2.48992100	-2.18231700	2.12666900
O	1.63672700	-0.31003600	1.94103700
H	2.58958700	-0.57563900	1.68338100
H	-1.99938100	-3.65629000	0.19252200
Au	0.31725500	1.11335400	-0.38922000
P	-1.13727000	0.97887300	-2.33602000
O	-0.52750000	1.72201400	-3.69788200

O	-1.40226400	-0.62045100	-2.73221900
O	-2.63975800	1.64809900	-2.05736300
C	-3.84331700	1.08566500	-2.50222900
C	-4.47491900	1.62044200	-3.61529100
C	-4.40524200	0.05372700	-1.76124500
C	-5.70153300	1.08831400	-4.00234300
H	-4.01281800	2.44441200	-4.15575300
C	-5.62978100	-0.47029200	-2.16184400
H	-3.87677600	-0.32621400	-0.88806400
C	-6.27660700	0.04369300	-3.28383300
H	-6.21046200	1.49936700	-4.87171800
H	-6.08305100	-1.27914100	-1.59234700
H	-7.23614700	-0.36536300	-3.59300800
C	-1.31226600	2.41433100	-4.63327100
C	-1.66341400	3.72965900	-4.35818000
C	-1.65556600	1.79444100	-5.82621100
C	-2.39657000	4.43903900	-5.30372600
H	-1.36272200	4.18212400	-3.41490200
C	-2.38633000	2.51834200	-6.76408600
H	-1.34402500	0.76889600	-6.01392400
C	-2.76182700	3.83353900	-6.50393400
H	-2.67884200	5.47013400	-5.10147000
H	-2.65988000	2.04775800	-7.70616800
H	-3.33237800	4.39246200	-7.24262600
C	-1.50124300	-1.11748800	-4.03802000
C	-0.33308500	-1.34895400	-4.75368400
C	-2.75098100	-1.43846200	-4.54984000
C	-0.42406000	-1.90235200	-6.02680000
H	0.62881400	-1.09205200	-4.31303500
C	-2.82583400	-1.99530500	-5.82312900
H	-3.64258800	-1.26256300	-3.95126600
C	-1.66897900	-2.22247500	-6.56358600
H	0.48292000	-2.08745000	-6.59860900
H	-3.79856800	-2.25477100	-6.23583300
H	-1.73666400	-2.65800800	-7.55825400

Structure and coordinates of **TS-o3**

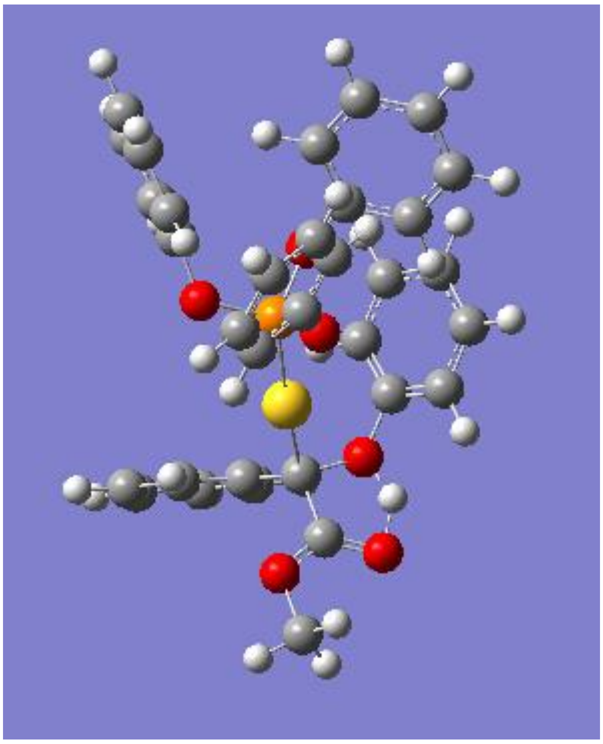

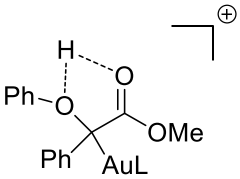

TS-o3
L = (PhO)$_3$P

C	0.98794900	2.36170800	4.79303200
C	1.35475500	1.50592400	3.75922700
C	1.24980600	1.92535000	2.43050000
C	0.78358800	3.21498000	2.15689200
C	0.43993700	4.07229100	3.19436500
C	0.53419800	3.64610500	4.51592000
H	1.06493600	2.01937900	5.82307200
H	1.71767700	0.50706900	3.99274400
H	0.69346700	3.55137800	1.12312200
H	0.08178000	5.07410600	2.96629900
H	0.25012500	4.31281200	5.32725500
C	1.66707300	1.04201000	1.29118300
C	3.06036000	1.24538300	0.79255600
O	3.80913200	0.25379700	0.74526800
O	3.39135500	2.44617700	0.43148200
C	4.73228200	2.63233900	-0.06800300
H	4.83393400	3.70577500	-0.22358900
H	5.45670400	2.27226300	0.66710400
H	4.85149500	2.08856100	-1.00938000
C	-0.37271400	-1.34210200	1.87828100
C	0.88380700	-1.33682300	1.29846500
C	1.25659800	-2.17884500	0.26514200
C	0.30581300	-3.07823800	-0.21008700
C	-0.97561400	-3.09934600	0.33499500
C	-1.31353300	-2.23523400	1.37410600
H	-0.60467100	-0.65354100	2.68880400
H	2.25616700	-2.12698400	-0.16575100
H	0.57252000	-3.76349500	-1.01238600
H	-2.31268800	-2.25989300	1.80239200
O	1.82411000	-0.38392500	1.77209800
H	-1.71630400	-3.79507100	-0.05386200
Au	0.32591500	1.06717800	-0.37269000
P	-1.30204600	0.90790700	-2.17565300
O	-0.61078700	0.49856900	-3.63631200

O	-2.37960700	-0.31068800	-1.78938900
O	-2.22301400	2.27021000	-2.44479700
C	-3.60109600	2.23979600	-2.71089800
C	-4.04556200	2.42424800	-4.01224600
C	-4.48115200	2.09881000	-1.64568100
C	-5.41696900	2.45088300	-4.24843300
H	-3.32563700	2.55578500	-4.81756300
C	-5.84867900	2.12295100	-1.89785800
H	-4.08934700	1.97128500	-0.63808400
C	-6.31767400	2.29488300	-3.19836400
H	-5.78111300	2.59832100	-5.26304500
H	-6.55005300	2.01280900	-1.07338500
H	-7.38807700	2.31719300	-3.39157500
C	-1.09053200	0.92876300	-4.88342300
C	-0.74042500	2.19678400	-5.32894900
C	-1.83453400	0.05508900	-5.66361000
C	-1.16729700	2.60584100	-6.58808600
H	-0.14462900	2.84744200	-4.69135300
C	-2.25196000	0.47746600	-6.92213000
H	-2.06983900	-0.93941200	-5.29061900
C	-1.92640200	1.75004000	-7.38290600
H	-0.90133900	3.59707700	-6.94931100
H	-2.83467800	-0.19738800	-7.54571400
H	-2.25708200	2.07394200	-8.36745400
C	-2.85705100	-1.25868600	-2.69945400
C	-2.04464500	-2.33710900	-3.03074300
C	-4.15006200	-1.13758900	-3.18801200
C	-2.53981900	-3.31219700	-3.89039000
H	-1.03946200	-2.39623200	-2.61345300
C	-4.63372000	-2.12359000	-4.04316500
H	-4.76098900	-0.28715500	-2.89083200
C	-3.83233000	-3.20513600	-4.39916700
H	-1.91373800	-4.15945200	-4.16332100
H	-5.64638300	-2.04267900	-4.43274900
H	-4.21728900	-3.96995600	-5.07033200
H	2.85001100	-0.52102100	1.38172300

Structure and coordinates of **Int-o4A**

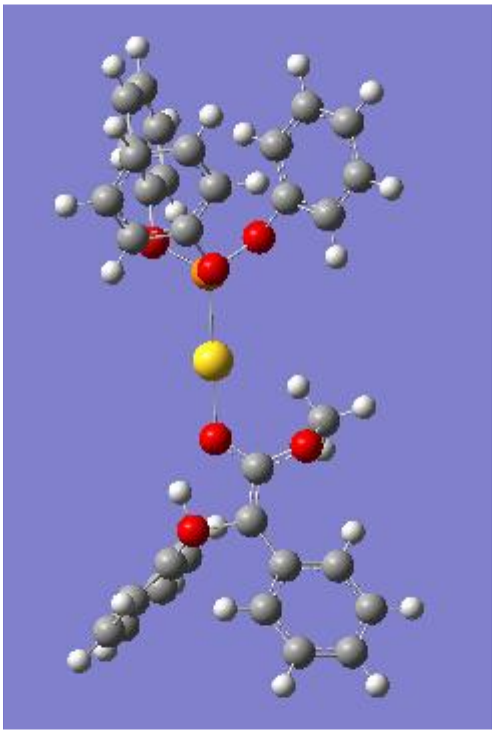

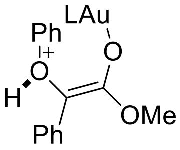

Int-o4A
L = $(PhO)_3P$

C	2.66539800	-1.70755900	4.80178800
C	2.73022400	-0.94903500	3.64252300
C	1.97929200	0.22947700	3.52584400
C	1.15472100	0.61738500	4.59008700
C	1.08473800	-0.15680700	5.74089200
C	1.83975600	-1.31926700	5.85376300
H	3.25745800	-2.61730000	4.87934700
H	3.37118900	-1.26193400	2.82047900
H	0.57915700	1.53952600	4.52732300
H	0.44102100	0.15802300	6.55986300
H	1.78611600	-1.92161700	6.75804300
C	2.06617700	1.02917400	2.32223400
C	2.99399700	1.24671200	1.35752200
O	2.69248700	2.07294000	0.37289900
O	4.20345300	0.67589500	1.42583600
C	4.53515100	-0.16171600	0.31647500
H	5.51120000	-0.59484000	0.54186300
H	3.78676900	-0.96031400	0.21042700
H	4.59405900	0.40881300	-0.61954800
C	-2.69612100	1.36572200	1.92412200
C	-2.76842900	0.31505600	1.01398000
C	-1.61044500	-0.21725400	0.45223800
C	-0.36355300	0.29669800	0.79492800
C	-0.33992200	1.33857600	1.70057500
C	-1.45951800	1.89585200	2.28250800
H	-3.60332000	1.77604000	2.36121100
H	-1.67433900	-1.03823600	-0.25800500
H	0.55916300	-0.10228100	0.37842300
H	-1.36366800	2.71678200	2.98953200
O	0.93425300	1.94201900	2.06412100
H	-3.73757800	-0.09557500	0.74015400
Au	4.19575600	3.34048200	-0.37363300
P	5.86118800	4.74300800	-1.30447200
O	5.27506000	5.68132000	-2.54382500
O	6.51018500	5.77973300	-0.18205900

O	7.08656600	3.81073300	-1.92875200
C	8.45070300	4.13691000	-1.85484800
C	9.08068800	4.67562400	-2.96687600
C	9.13878300	3.83849200	-0.68623200
C	10.44570100	4.93659100	-2.89230500
H	8.51048700	4.87387100	-3.87230600
C	10.50200800	4.10892500	-0.62602700
H	8.60483300	3.40200600	0.15618500
C	11.15455800	4.66132000	-1.72599800
H	10.95649500	5.35694100	-3.75608300
H	11.05564500	3.88135900	0.28252300
H	12.22143100	4.86832400	-1.67644100
C	5.96029700	5.89603300	-3.75119200
C	5.91871800	4.90739300	-4.72496500
C	6.59437800	7.11202000	-3.96021000
C	6.55149300	5.13968400	-5.94164600
H	5.39779000	3.97292100	-4.52332400
C	7.21712600	7.33216700	-5.18563800
H	6.58731700	7.87056800	-3.17999200
C	7.20292500	6.34958200	-6.17166000
H	6.52877300	4.37365200	-6.71381700
H	7.71383100	8.28286100	-5.36804300
H	7.69220200	6.52918700	-7.12665700
C	6.95576900	7.07622700	-0.49780100
C	6.02102000	8.09945800	-0.58463100
C	8.31579100	7.30538100	-0.64165500
C	6.46694800	9.39138400	-0.84523700
H	4.96374500	7.87704400	-0.45370200
C	8.74638500	8.60308100	-0.90067500
H	9.01926500	6.48260700	-0.53554500
C	7.82727000	9.64289100	-1.00915900
H	5.74615000	10.20306000	-0.91840000
H	9.81062500	8.79876600	-1.01405300
H	8.17173700	10.65476900	-1.21163000
H	1.31415400	2.43691300	1.25532500

Structure and coordinates of **Int-o4**

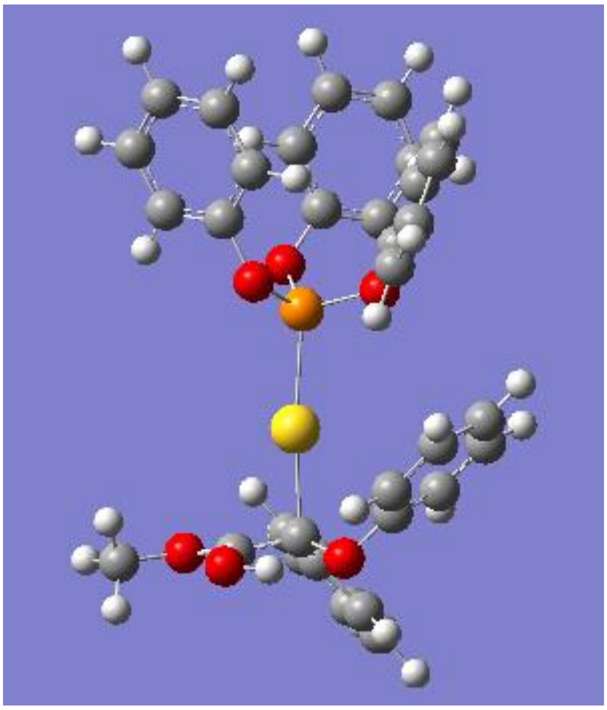

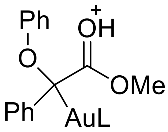

Int-o4
L = (PhO)$_3$P

C	0.96551200	2.14310700	4.82054800
C	1.36397300	1.32565500	3.76803600
C	1.30469800	1.79457200	2.45336500
C	0.84166800	3.09139800	2.21156500
C	0.45745100	3.90821000	3.26785500
C	0.51413800	3.43555800	4.57550400
H	1.01384900	1.76499200	5.83980900
H	1.72220600	0.31703000	3.96501600
H	0.77951700	3.46815900	1.18971400
H	0.09843300	4.91522600	3.06506800
H	0.20261100	4.07182700	5.40116400
C	1.76188100	0.90580200	1.33029700
C	2.98682000	1.27040800	0.65566100
O	3.79515800	0.35224500	0.21547900
O	3.30507600	2.50401000	0.46632100
C	4.47202600	2.81265100	-0.32779100
H	4.52519800	3.90031200	-0.33868600
H	5.36289500	2.38282500	0.13707000
H	4.34009300	2.41665100	-1.33916500
C	-0.42938900	-1.08836600	1.89951400
C	0.83182200	-1.29800800	1.34752700
C	1.06366500	-2.32829400	0.44370700
C	0.00756800	-3.15632900	0.07394100
C	-1.26540600	-2.94460800	0.59549400
C	-1.47969500	-1.91311700	1.50557400
H	-0.58264200	-0.28678100	2.62018800
H	2.06302400	-2.47842900	0.03460400
H	0.18283600	-3.96902100	-0.62903700
H	-2.47050600	-1.75271000	1.92527100
O	1.90287200	-0.46915100	1.67041300
H	-2.09109500	-3.58528000	0.29315700
Au	0.36724800	1.03903300	-0.36310400
P	-1.27598100	0.94339100	-2.15126400
O	-0.55936000	0.55230600	-3.60449100
O	-2.36201100	-0.27132000	-1.78736000

O	-2.20138800	2.30427600	-2.41481400
C	-3.57870400	2.25299700	-2.68860800
C	-4.01815300	2.41924600	-3.99397300
C	-4.46240300	2.10651200	-1.62736400
C	-5.38851000	2.42303100	-4.23829500
H	-3.29581300	2.55447400	-4.79646700
C	-5.82850300	2.10731900	-1.88757500
H	-4.07481300	1.99166500	-0.61669500
C	-6.29260900	2.26171100	-3.19203000
H	-5.74882800	2.55625400	-5.25622800
H	-6.53292200	1.99184800	-1.06647900
H	-7.36208000	2.26536200	-3.39155000
C	-1.03437300	0.96521300	-4.85935300
C	-0.71258700	2.24061700	-5.30503400
C	-1.74428500	0.06863100	-5.64527200
C	-1.13246500	2.63328700	-6.57180500
H	-0.14493000	2.90988400	-4.66108900
C	-2.15557800	0.47526600	-6.91092500
H	-1.95788300	-0.93036100	-5.27097400
C	-1.85754900	1.75425900	-7.37295100
H	-0.88880400	3.63007800	-6.93349900
H	-2.71153300	-0.21735700	-7.53940200
H	-2.18299300	2.06505500	-8.36343600
C	-2.78507900	-1.24318100	-2.69922200
C	-1.93674800	-2.30711300	-2.97983600
C	-4.05859600	-1.15390100	-3.24260300
C	-2.37392800	-3.30374000	-3.84606500
H	-0.95093800	-2.33802000	-2.51595300
C	-4.48464600	-2.16230600	-4.10278800
H	-4.69894400	-0.31187500	-2.98584500
C	-3.64620700	-3.23069300	-4.40971900
H	-1.71943500	-4.14097400	-4.08015200
H	-5.48157800	-2.10881800	-4.53533900
H	-3.98706800	-4.01286100	-5.08474900
H	3.43968000	-0.50084600	0.56695300

Structure and coordinates of **Int-o5**

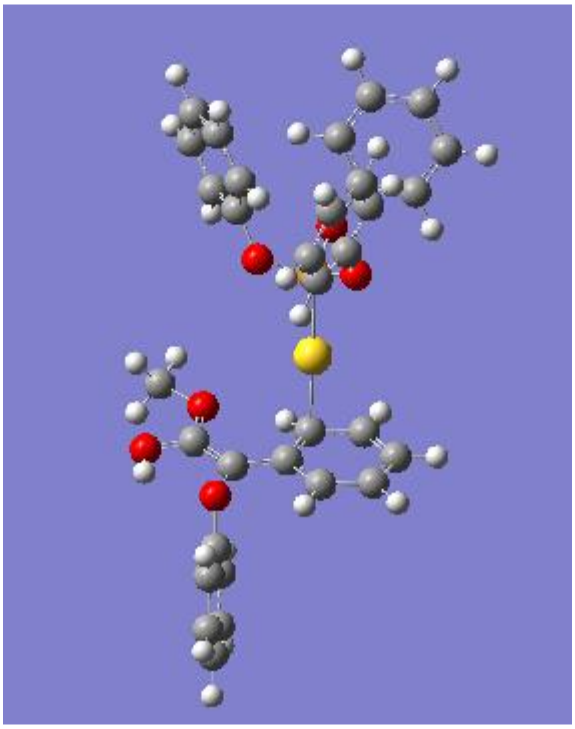

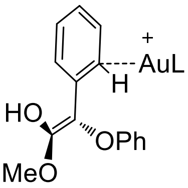

Int-o5
L = (PhO)3P

C	1.09356400	-2.83207400	-5.71342900
C	1.32675900	-1.70450300	-4.94771300
C	0.38424300	-1.25820200	-3.99428900
C	-0.86226400	-1.97838900	-3.91034500
C	-1.04352500	-3.16442800	-4.68934200
C	-0.08756400	-3.57816900	-5.59596800
H	1.85730900	-3.15154300	-6.42033400
H	2.26400100	-1.16438500	-5.05376300
H	-1.48222600	-1.84252000	-3.02361000
H	-1.95431100	-3.74198800	-4.53764200
H	-0.23996700	-4.47247200	-6.19409000
C	0.71349300	-0.14750700	-3.14960200
C	-0.10379100	0.52542700	-2.27775600
O	0.32649100	1.57269500	-1.58995800
O	-1.37473400	0.20677300	-2.10447300
C	-2.13842500	0.96161700	-1.15199400
H	-3.13646100	0.52270100	-1.17538000
H	-1.70293100	0.86557000	-0.15272800
H	-2.17623600	2.01637200	-1.43925300
C	4.29991300	0.28554700	-3.14341500
C	3.06096900	-0.16533800	-2.70171200
C	2.95327900	-1.14361300	-1.72233500
C	4.12050100	-1.67508500	-1.17744500
C	5.36831300	-1.23517300	-1.60366500
C	5.45336100	-0.25247400	-2.58754800
H	4.33887400	1.05320000	-3.91416000
H	1.97654200	-1.48943800	-1.38893800
H	4.04513400	-2.44224600	-0.40913100
H	6.42536500	0.09799100	-2.92894000
O	1.95931900	0.44745600	-3.29634700
H	1.24265500	1.75219400	-1.88021900
H	6.27345300	-1.65640800	-1.17209500
Au	-2.00582100	-0.75273000	-5.36141400
P	-3.24547900	0.61996400	-6.91664200
O	-3.13881100	2.23421800	-6.52195000

O	-2.66134900	0.44817300	-8.46591300
O	-4.86161000	0.22372200	-6.96396300
C	-5.63668400	0.24243900	-8.13618700
C	-6.52453400	1.28827500	-8.34092000
C	-5.54268300	-0.82766100	-9.01681700
C	-7.32997300	1.26418400	-9.47552900
H	-6.59028000	2.09482700	-7.61377600
C	-6.35129600	-0.83525500	-10.14865900
H	-4.83968200	-1.63375000	-8.81459300
C	-7.24100800	0.21088000	-10.38138200
H	-8.03435400	2.07545600	-9.64689900
H	-6.28700100	-1.66565100	-10.84867100
H	-7.87333500	0.19936600	-11.26659600
C	-4.18217600	3.15881500	-6.68928700
C	-5.14887100	3.24951300	-5.69608400
C	-4.18044700	3.99854800	-7.79335600
C	-6.15536900	4.20107100	-5.82480400
H	-5.10693500	2.57810800	-4.84021200
C	-5.19307900	4.94651000	-7.90748300
H	-3.39372900	3.91253500	-8.53985900
C	-6.18076600	5.04665900	-6.93161300
H	-6.91953400	4.28424100	-5.05474600
H	-5.20418200	5.61258800	-8.76766700
H	-6.96746300	5.79179800	-7.02881400
C	-2.49371000	1.50859900	-9.36939900
C	-1.35719600	2.29926200	-9.25986500
C	-3.42472000	1.69313200	-10.38099800
C	-1.15791100	3.31811100	-10.18559400
H	-0.64459800	2.11204200	-8.45838400
C	-3.20889900	2.71365100	-11.30272600
H	-4.29315300	1.04079600	-10.44466100
C	-2.08413200	3.52807400	-11.20469800
H	-0.27236500	3.94588400	-10.11276400
H	-3.92834500	2.86844200	-12.10406400
H	-1.92349500	4.32252300	-11.93035700

Structure and coordinates of **Int-o5A**

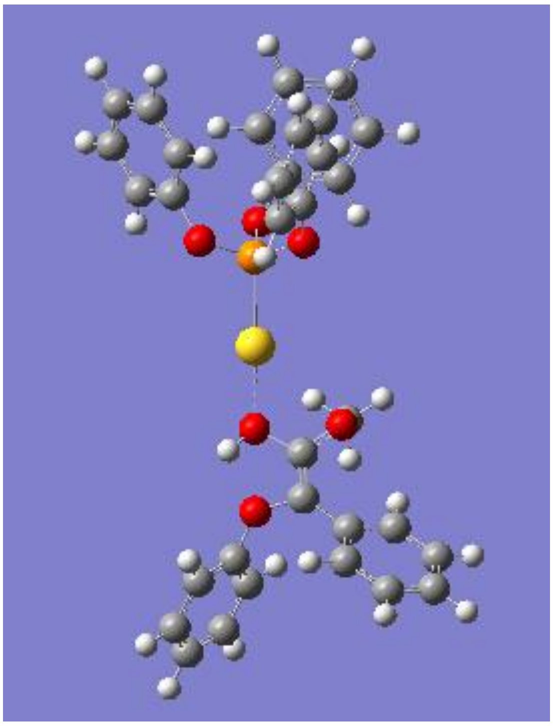

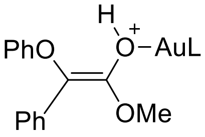

Int-o5A
L = (PhO)$_3$P

C	2.75383800	-0.64457800	5.20491200
C	2.79445200	-0.30026800	3.86128000
C	1.88940600	0.63358900	3.34265200
C	0.93576400	1.20197700	4.19664400
C	0.89715200	0.84998800	5.53882600
C	1.80587200	-0.07183200	6.04831100
H	3.46266500	-1.37248400	5.59481000
H	3.53355200	-0.76329200	3.21182800
H	0.22614700	1.92766700	3.80410300
H	0.15196300	1.30045700	6.19122500
H	1.77343000	-0.34808400	7.10019000
C	1.91281100	1.04174200	1.93708200
C	2.95380000	1.01854700	1.08968700
O	2.76764400	1.49609800	-0.22096300
O	4.22728900	0.73088800	1.34233500
C	4.72255900	-0.44460000	0.68190400
H	5.73933400	-0.59457200	1.04901600
H	4.09688500	-1.31028800	0.93612800
H	4.73315400	-0.30539100	-0.40652100
C	-2.77009700	0.92088400	1.14971700
C	-2.77981600	-0.46897300	1.06377100
C	-1.58082200	-1.17247600	1.10261200
C	-0.36794800	-0.49943300	1.22364500
C	-0.38359000	0.88558300	1.31156600
C	-1.56876000	1.60773200	1.27763300
H	-3.70476300	1.47735400	1.12365400
H	-1.58264400	-2.25863500	1.03802900
H	0.57284800	-1.04657900	1.25711400
H	-1.53259400	2.69277300	1.35542300
O	0.78836700	1.64220600	1.39765400
H	-3.72275300	-1.00282600	0.96955600
Au	4.29779800	2.94558900	-0.82578100
P	5.93221100	4.49997200	-1.54288100
O	5.24113200	5.74818900	-2.38708800
O	6.74251800	5.13758400	-0.24705000

O	7.02617900	3.75556900	-2.54135000
C	8.40566300	4.03660000	-2.54702700
C	8.92602800	4.85822000	-3.53523900
C	9.20720500	3.41774600	-1.59745000
C	10.30063300	5.07629900	-3.55536900
H	8.26567200	5.30418500	-4.27663500
C	10.57860800	3.64953500	-1.62879200
H	8.75595900	2.76761100	-0.84991900
C	11.12456000	4.48155400	-2.60353900
H	10.72764500	5.71596800	-4.32481500
H	11.22154000	3.17410800	-0.89118500
H	12.19767300	4.65827700	-2.62714700
C	5.83112900	6.32999100	-3.52666600
C	5.64803300	5.70787900	-4.75385400
C	6.51423400	7.52967000	-3.39798800
C	6.18651100	6.30318900	-5.88985500
H	5.09343500	4.77285700	-4.80897100
C	7.04383300	8.11455700	-4.54484000
H	6.61441100	7.99619900	-2.42018900
C	6.88728500	7.50273300	-5.78552900
H	6.05313500	5.82905900	-6.85991400
H	7.57900700	9.05826500	-4.46382100
H	7.30411500	7.96634300	-6.67706200
C	7.21175100	6.46540000	-0.19439900
C	6.32061600	7.46556600	0.16891000
C	8.55387700	6.71882700	-0.43293100
C	6.79143500	8.77015700	0.27629800
H	5.27853900	7.21758300	0.36182300
C	9.00972600	8.02881300	-0.31791200
H	9.22534300	5.90125700	-0.68676800
C	8.13298400	9.05305800	0.02881600
H	6.10669100	9.56655800	0.55978400
H	10.06066800	8.24530800	-0.49768900
H	8.49780700	10.07404900	0.11748600
H	1.83339300	1.80126300	-0.25901400

Structure and coordinates of **TS-o6-2w**

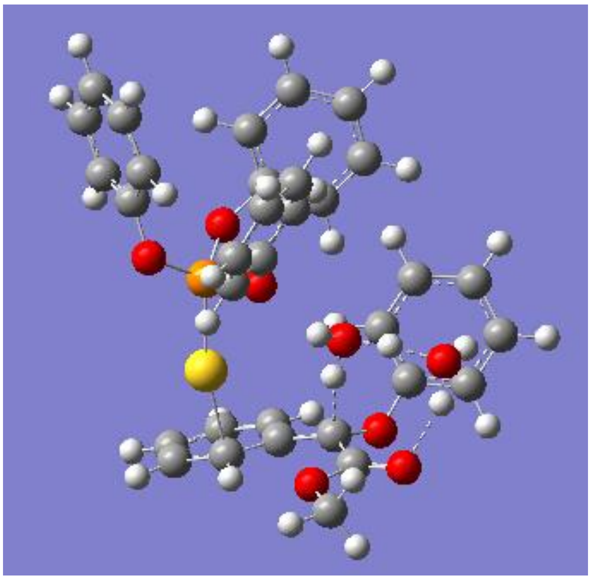

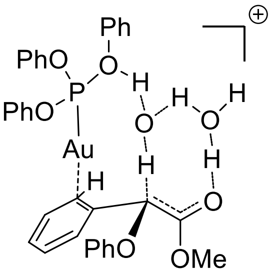

TS-o6-2w
L = (PhO)$_3$P

C	-3.83165000	-4.98433700	-8.75525400
C	-3.79744600	-3.85554900	-7.96096600
C	-4.96750000	-3.32865200	-7.35915300
C	-6.21255100	-3.99468600	-7.69101400
C	-6.19205500	-5.19345600	-8.48206800
C	-5.02576300	-5.67128600	-9.03219000
H	-2.89462000	-5.36209100	-9.16188300
H	-2.84339900	-3.38695500	-7.73563000
H	-7.05966300	-3.84559300	-7.02047400
H	-7.13390500	-5.72227600	-8.62405900
H	-5.02127100	-6.56941900	-9.64382300
C	-4.85230600	-2.15508900	-6.52033900
C	-5.77440800	-1.75205500	-5.46439100
O	-5.51586400	-0.89215300	-4.62726700
O	-7.01032800	-2.27884300	-5.56401600
C	-7.95101300	-1.82164800	-4.58820300
H	-8.87856800	-2.35671300	-4.79877400
H	-7.59808700	-2.04984200	-3.57841900
H	-8.10357100	-0.74069700	-4.67373400
C	-2.85929200	-0.47304400	-7.99472200
C	-2.88766100	-0.77752100	-6.63366800
C	-2.20415100	0.02776200	-5.71967700
C	-1.51617100	1.15231100	-6.16729000
C	-1.50921200	1.47960700	-7.52394400
C	-2.17399000	0.65871700	-8.43084300
H	-3.37312200	-1.11752600	-8.70914500
H	-2.23808600	-0.24900800	-4.66703700
H	-0.98234600	1.77570600	-5.45213200
H	-2.14833100	0.88956700	-9.49558500
H	-5.09178700	0.73589600	-5.17508600
O	-5.72347900	0.10271300	-7.77053600
H	-5.44318300	-0.88193200	-7.38902600
H	-6.65364000	0.18014800	-8.07056700
Au	-6.80695500	-2.68450800	-9.34167300
P	-7.41428500	-0.82594600	-10.77384900
O	-6.14126800	-0.27197000	-11.68679200
O	-8.72035900	-0.94006400	-11.79024400
O	-7.78876000	0.39118500	-9.65625900
C	-8.74520800	1.40054600	-9.88243300

C	-8.32159700	2.66069000	-10.27810000
C	-10.07920000	1.11227400	-9.62752600
C	-9.27839300	3.65839500	-10.44007400
H	-7.26390900	2.85358900	-10.44727000
C	-11.02344400	2.12063800	-9.79462100
H	-10.36482300	0.11028500	-9.31164800
C	-10.62423900	3.39066000	-10.20483600
H	-8.96444800	4.65296900	-10.74952700
H	-12.07330400	1.91145700	-9.60073700
H	-11.36565100	4.17630200	-10.33349800
C	-5.71659300	1.06405800	-11.74937000
C	-4.91666000	1.55301400	-10.72523800
C	-6.04129100	1.82586900	-12.86151100
C	-4.44569000	2.85931700	-10.81224800
H	-4.67156200	0.91566400	-9.87620300
C	-5.55750000	3.12900300	-12.93613500
H	-6.65090000	1.39456600	-13.65252700
C	-4.76799200	3.64919600	-11.91386300
H	-3.81880700	3.25601300	-10.01498000
H	-5.79994800	3.73864000	-13.80400700
H	-4.39402500	4.66859000	-11.98070000
C	-8.80552400	-0.27487200	-13.02975800
C	-8.14213500	-0.81647100	-14.12247700
C	-9.60629500	0.85248600	-13.13458800
C	-8.27667300	-0.19471800	-15.35965100
H	-7.53335400	-1.71019900	-13.99942700
C	-9.73312000	1.46073800	-14.38010000
H	-10.13309000	1.23105400	-12.26090800
C	-9.06794500	0.94453800	-15.48870600
H	-7.76509700	-0.60817500	-16.22616600
H	-10.36261400	2.34229800	-14.48048900
H	-9.17484500	1.42381800	-16.45948500
O	-5.00232500	1.44267500	-5.86785100
H	-5.43656100	0.81139500	-6.95350500
H	-4.04012800	1.58715000	-5.95146300
O	-3.52707200	-1.87212900	-6.12977100
H	-0.96897700	2.35747900	-7.87183400

Structure and coordinates of **TS-o6-1w**

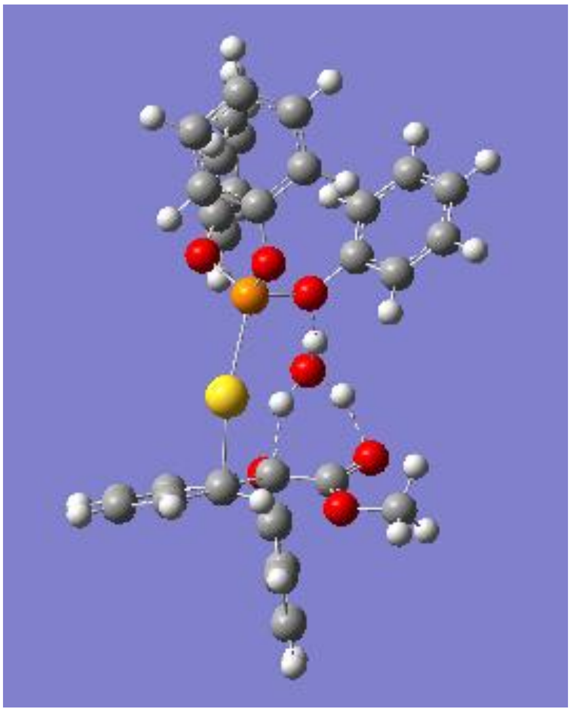

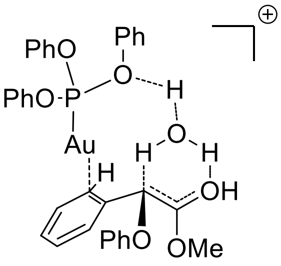

TS-o6-1w
L = $(PhO)_3P$

C	-3.15611800	-4.77943200	-8.16626300
C	-3.40697200	-3.49634700	-7.73572200
C	-4.55603200	-3.18542800	-6.96094100
C	-5.50035600	-4.27430500	-6.73155500
C	-5.15839400	-5.60627900	-7.16134600
C	-4.02431000	-5.85724500	-7.88641300
H	-2.24219300	-4.96991300	-8.72726300
H	-2.69667000	-2.70233500	-7.95009500
H	-6.15115900	-4.19309200	-5.85826300
H	-5.83246500	-6.41525200	-6.88209300
H	-3.78159700	-6.86233000	-8.22029700
C	-4.78012800	-1.83214700	-6.55629500
C	-5.71887600	-1.35256800	-5.55124400
O	-6.06600400	-0.16589500	-5.48426700
O	-6.25604500	-2.27762600	-4.75325000
C	-7.25029700	-1.81073000	-3.83420500
H	-7.56969100	-2.69101800	-3.27451400
H	-6.82313800	-1.06234700	-3.16089300
H	-8.09511400	-1.36680400	-4.37150900
C	-1.94464700	0.39213800	-6.18577400
C	-2.80157300	-0.67098000	-5.91808400
C	-2.64233800	-1.44980500	-4.77723900
C	-1.60786700	-1.14623000	-3.89385400
C	-0.74554500	-0.08660100	-4.14594700
C	-0.91835700	0.68012600	-5.29689600
H	-2.10277200	0.97853500	-7.08873200
H	-3.30950200	-2.28677100	-4.57582100
H	-1.47962800	-1.75321300	-2.99948000
H	-0.24919500	1.51309600	-5.50409000
O	-3.78307400	-0.90094200	-6.86810600
H	-6.16534200	0.28828200	-7.08639500
H	0.05844600	0.14256900	-3.45015700
O	-6.14308800	-0.09988600	-8.03588800
H	-5.66690900	-1.01237400	-7.74122200
H	-7.06521700	-0.30752900	-8.34470700

Au	-6.91308600	-3.71906900	-8.29335500
P	-8.40568600	-2.55388200	-9.81420500
O	-7.79788000	-2.31791000	-11.33984400
O	-9.96667000	-3.08408800	-9.95069200
O	-8.51514300	-0.98806800	-9.13216700
C	-9.71220300	-0.24166400	-9.10032500
C	-9.89356900	0.78438100	-10.01478400
C	-10.63423400	-0.52129600	-8.10086800
C	-11.05424500	1.54782600	-9.92640400
H	-9.13769600	0.98075400	-10.77234300
C	-11.79091400	0.24809700	-8.02892200
H	-10.44430200	-1.33313200	-7.40009800
C	-12.00172300	1.27914000	-8.94220400
H	-11.21404900	2.35924500	-10.63300500
H	-12.52711500	0.04344700	-7.25473300
H	-12.90601600	1.88066600	-8.88151700
C	-7.80348500	-1.08293200	-12.01193300
C	-6.78185000	-0.18324500	-11.73804800
C	-8.77465800	-0.83169400	-12.96893500
C	-6.74752200	1.02092600	-12.43348400
H	-6.02192900	-0.43659600	-10.99953700
C	-8.72274000	0.37488600	-13.66161100
H	-9.54269000	-1.57507800	-13.17133000
C	-7.71918800	1.30149100	-13.39204300
H	-5.95309800	1.73646600	-12.23273600
H	-9.47188600	0.58530400	-14.42185800
H	-7.68574600	2.24087300	-13.93955900
C	-10.75592900	-2.96992300	-11.11419700
C	-10.54198500	-3.85922500	-12.15787600
C	-11.76709700	-2.02117500	-11.14282000
C	-11.36186400	-3.77811000	-13.27922400
H	-9.75056900	-4.60300900	-12.08666600
C	-12.58078600	-1.95679200	-12.26997400
H	-11.92086800	-1.36143300	-10.29149000
C	-12.37673000	-2.82584000	-13.33829500
H	-11.20905000	-4.46917300	-14.10527600
H	-13.38330400	-1.22321900	-12.30775100
H	-13.01768300	-2.77053700	-14.21543000

Structure and coordinates of **Int-o6**

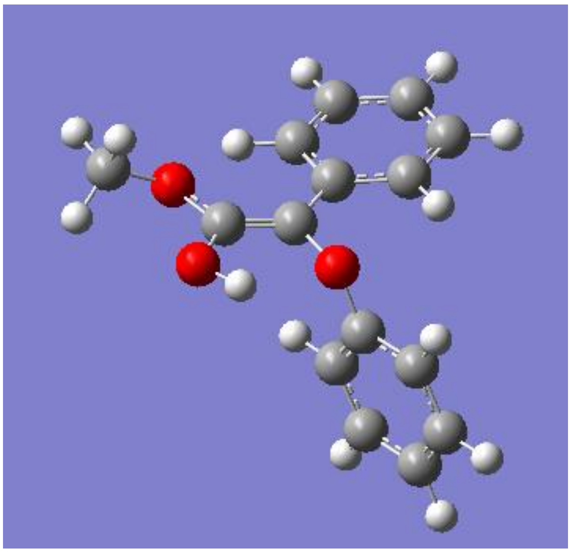

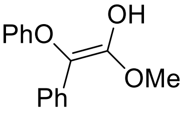

Int-o6

C	-3.15029200	-4.06542100	-8.49450500
C	-2.96620000	-2.96732300	-7.66613900
C	-3.81964400	-2.73580300	-6.57600600
C	-4.85654600	-3.65290400	-6.34075600
C	-5.03675200	-4.74457800	-7.17862300
C	-4.18777000	-4.96130700	-8.25969500
H	-2.47361500	-4.22004200	-9.33373800
H	-2.15536400	-2.26904700	-7.86463700
H	-5.52063500	-3.50644800	-5.49380200
H	-5.84906700	-5.44155100	-6.97631500
H	-4.33126100	-5.82228200	-8.91005500
C	-3.58866000	-1.56943800	-5.73261000
C	-4.39451700	-0.99156400	-4.80915800
O	-4.04405900	0.13425800	-4.16370200
O	-5.58429000	-1.48566500	-4.46104800
C	-6.52529700	-0.58092600	-3.89618400
H	-7.44416800	-1.15694100	-3.76843500
H	-6.18253700	-0.20551200	-2.92626000
H	-6.70979100	0.26616400	-4.56804600
C	-0.05950600	-0.78202200	-5.90540200
C	-1.23085500	-1.36662200	-5.43081400
C	-1.18696400	-2.38916100	-4.49004600
C	0.05107600	-2.82104800	-4.02266700
C	1.22900600	-2.24402000	-4.48311300
C	1.16699000	-1.22204900	-5.42721800
H	-0.13575000	0.01054900	-6.64778200
H	-2.11002500	-2.84296500	-4.13613800
H	0.08916400	-3.62358900	-3.28784400
H	2.08249600	-0.76560600	-5.80000100
O	-2.40412900	-0.84874900	-5.94081600
H	2.19100300	-2.59162600	-4.11202400
H	-3.21151900	0.42883800	-4.57612100

Structure and coordinates of **TS-o7-2w**

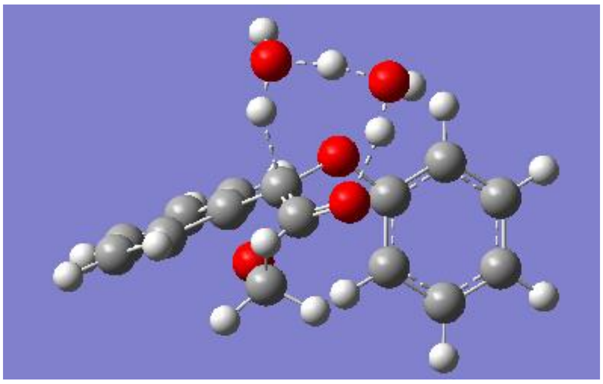

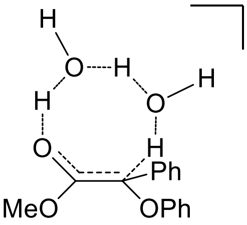

TS-o7-2w

C	-5.06022500	-4.47114900	-8.12674400
C	-4.64023800	-3.27160300	-7.56517500
C	-5.22939200	-2.76727100	-6.39348300
C	-6.27821300	-3.51484100	-5.82758900
C	-6.68952100	-4.71330200	-6.39268000
C	-6.08558700	-5.20698600	-7.54533000
H	-4.57340900	-4.83278300	-9.03198700
H	-3.83920400	-2.71396600	-8.04571800
H	-6.77247700	-3.14860000	-4.93294000
H	-7.50178400	-5.26716100	-5.92270900
H	-6.41348700	-6.14766400	-7.98417400
C	-4.79757500	-1.46592600	-5.86082500
C	-4.79868300	-1.04299500	-4.49837300
O	-4.27426500	0.00970000	-4.07187700
O	-5.47897600	-1.82715300	-3.64117400
C	-5.62004600	-1.32208600	-2.32045500
H	-6.18990900	-2.07823500	-1.77578900
H	-4.64214100	-1.17281900	-1.85114000
H	-6.15845100	-0.36758500	-2.31844600
C	-1.52533600	-0.41851600	-7.11653400
C	-2.46344200	-1.14334600	-6.38077100
C	-2.04369400	-2.11442400	-5.47493900
C	-0.68212800	-2.33606500	-5.30255800
C	0.26207500	-1.60790100	-6.01959400
C	-0.16791000	-0.64926600	-6.93169100
H	-1.87963500	0.30402200	-7.85371000
H	-2.77833700	-2.69341400	-4.91908700
H	-0.35686900	-3.09570500	-4.59351600
H	0.55652500	-0.07797800	-7.50992900
O	-3.77447300	-0.82774500	-6.62147200
H	-4.12111300	1.16774100	-5.04184600
H	1.32453400	-1.79020900	-5.87270100
O	-4.18292300	1.80722300	-5.85798300
O	-6.13822000	0.69607300	-6.73877500
H	-5.16043400	1.35589300	-6.37058300
H	-5.85706200	-0.26654900	-6.36672600
H	-3.44175400	1.50021800	-6.40867100
H	-6.14412800	0.60311500	-7.70367200

Structure and coordinates of TS-**o7-1w**

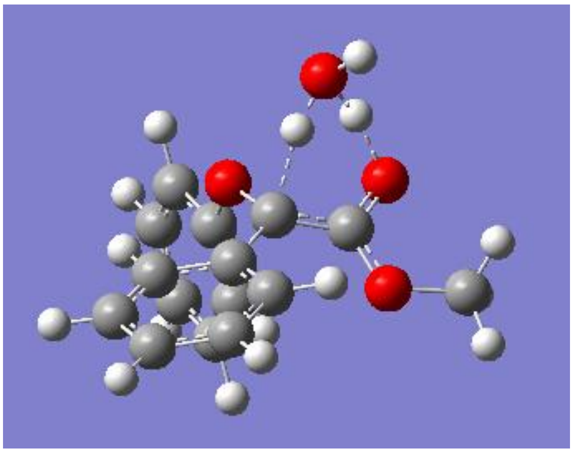

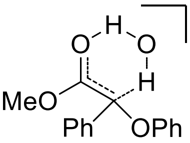

TS-o7-1w

C	-5.03480900	-4.56654800	-8.09440700
C	-4.57213200	-3.35864800	-7.58604500
C	-5.19923500	-2.74782000	-6.49224000
C	-6.32343600	-3.38588600	-5.94421300
C	-6.77817700	-4.59455600	-6.45101700
C	-6.13644700	-5.19736600	-7.52934400
H	-4.52314800	-5.01824100	-8.94331600
H	-3.71543600	-2.87486400	-8.04969200
H	-6.84925600	-2.92680200	-5.10896300
H	-7.65025100	-5.06766800	-6.00087100
H	-6.49731900	-6.14425600	-7.92712800
C	-4.73803800	-1.43393800	-6.01148800
C	-4.73462400	-1.02061100	-4.64020500
O	-4.47488900	0.17638500	-4.30961000
O	-5.12031000	-1.88282200	-3.70437800
C	-5.30373800	-1.36022000	-2.39034100
H	-5.64184100	-2.20314900	-1.78495400
H	-4.36222000	-0.96218000	-1.99982100
H	-6.05447100	-0.56258300	-2.38744400
C	-1.38419200	-0.47023700	-7.04879700
C	-2.38340900	-1.18923600	-6.39271100
C	-2.04728000	-2.17961400	-5.47202300
C	-0.70360400	-2.43406800	-5.20943600
C	0.29930200	-1.71976300	-5.85332900
C	-0.05070800	-0.73727900	-6.77718100
H	-1.68310800	0.29551300	-7.76190300
H	-2.82233300	-2.76184100	-4.97754700
H	-0.44461500	-3.21193100	-4.49257200
H	0.72403900	-0.16994600	-7.29080500
O	-3.66717500	-0.85864700	-6.73921200
H	-4.91561800	0.85766400	-5.31924300
H	1.34604300	-1.92757700	-5.64028900
O	-5.55021000	1.00691300	-6.27452200
H	-5.55652600	-0.12242800	-6.36914900
H	-6.43704800	1.28664500	-5.99884800

Structure and coordinates of **Int-o7**

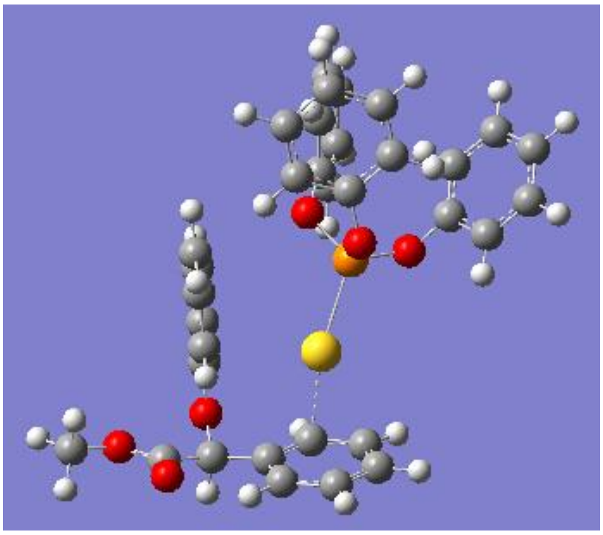

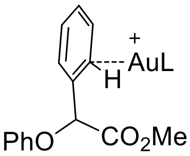

Int-o7
L = (PhO)$_3$P

C	-7.18141400	-5.15764300	-7.56424200
C	-6.03705500	-4.37384500	-7.45603600
C	-6.08903500	-3.14614100	-6.79236800
C	-7.32570500	-2.69196900	-6.25834100
C	-8.48220900	-3.50261500	-6.39034700
C	-8.40371500	-4.72368000	-7.04718300
H	-7.12253900	-6.12100700	-8.06675400
H	-5.08981500	-4.70770200	-7.87756400
H	-7.33402700	-1.84774400	-5.56258200
H	-9.41779700	-3.17003200	-5.94481400
H	-9.28935100	-5.34719000	-7.14190700
C	-4.85078300	-2.28846600	-6.70563100
C	-3.72204100	-3.10278100	-6.06912500
O	-2.85976900	-3.67459400	-6.68422300
O	-3.88263500	-3.12178500	-4.74795400
C	-2.91916000	-3.89757900	-4.02292700
H	-3.21088200	-3.83077600	-2.97438900
H	-2.93406900	-4.93698700	-4.36498700
H	-1.91681600	-3.48462100	-4.17207500
C	-4.11470900	0.46303600	-7.96499900
C	-3.64383000	-0.83588200	-8.12434400
C	-2.31565300	-1.09202400	-8.43708300
C	-1.44372700	-0.01810800	-8.59087600
C	-1.89850300	1.28767800	-8.43061000
C	-3.23427000	1.52827500	-8.11890900
H	-5.17257100	0.62868100	-7.75119300
H	-1.98889600	-2.12298500	-8.54698900
H	-0.40033100	-0.20635400	-8.83628000
H	-3.59459500	2.54912100	-8.00310500
H	-5.04903400	-1.41053400	-6.06647100
Au	-7.62707500	-1.53555100	-8.18995900
P	-7.83330700	-0.05361500	-10.07008500
O	-6.43087900	0.83309100	-10.14356100
O	-7.97323600	-0.81785200	-11.53780100
O	-9.11700600	0.99041200	-9.91955600
C	-9.81581600	1.52850400	-11.01743700
C	-9.50993600	2.80633000	-11.46124000
C	-10.84519100	0.77970200	-11.57168300
C	-10.25471100	3.33856500	-12.50956600
H	-8.71487800	3.37399100	-10.98254000

C	-11.57989900	1.32532900	-12.61931700
H	-11.06004400	-0.21346600	-11.18177100
C	-11.28204200	2.60158800	-13.09211000
H	-10.02995700	4.34105500	-12.86747700
H	-12.39015900	0.75153700	-13.06400400
H	-11.85991000	3.02677900	-13.90981100
C	-6.34668400	2.21004500	-10.38075500
C	-6.64202900	3.08541900	-9.34357700
C	-5.89455600	2.65135300	-11.61549900
C	-6.49146400	4.45188200	-9.55837600
H	-6.98974800	2.69202200	-8.38833000
C	-5.74488300	4.02082900	-11.81322500
H	-5.65815400	1.92810700	-12.39415800
C	-6.04506600	4.91924100	-10.79257300
H	-6.72097500	5.15173200	-8.75761800
H	-5.38699200	4.38605800	-12.77358300
H	-5.92456900	5.98800900	-10.95619300
C	-7.28743500	-0.37824600	-12.68627300
C	-5.95112300	-0.72412800	-12.83726600
C	-7.98466900	0.32472600	-13.65728000
C	-5.29025800	-0.33692300	-13.99843400
H	-5.44411500	-1.27900900	-12.04939500
C	-7.31049900	0.70023900	-14.81550900
H	-9.03731700	0.55732000	-13.50853400
C	-5.96719000	0.37645000	-14.98550700
H	-4.24279900	-0.59852000	-14.13249300
H	-7.84394900	1.24636200	-15.59063200
H	-5.44675200	0.67282900	-15.89374200
O	-4.53847000	-1.89789800	-8.02016800
H	-1.21011000	2.12138100	-8.55229200

Structure and coordinates of **Pro-o8**

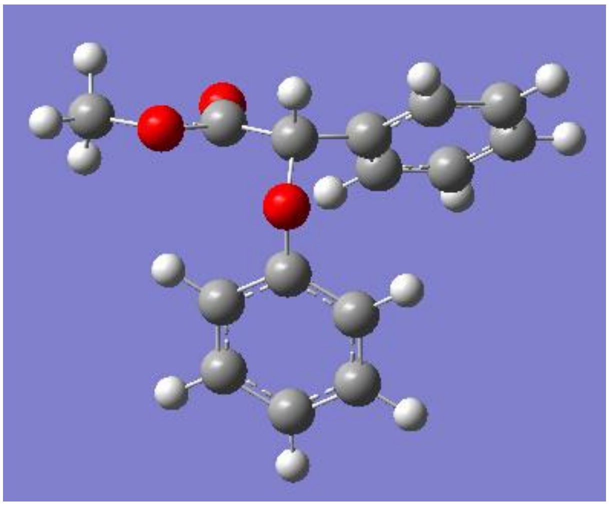

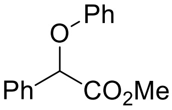

Pro-o8

C	0.63122700	3.42877200	3.92219000
C	1.14539200	2.94894700	2.72356100
C	0.51033500	1.89799700	2.05726100
C	-0.63854900	1.33855900	2.61745000
C	-1.15352000	1.81704200	3.81636000
C	-0.51692000	2.86594100	4.47083800
H	1.13390700	4.24879600	4.43206500
H	2.04447700	3.39349400	2.30266500
H	-1.13119300	0.50979500	2.10700700
H	-2.05355200	1.37254800	4.23776800
H	-0.91616800	3.24671500	5.40964700
C	1.08005600	1.24809100	0.82657700
C	1.65388000	2.21926900	-0.20539300
O	1.63483900	3.42314400	-0.14174000
O	2.15453900	1.53958000	-1.24767700
C	2.73086100	2.35166000	-2.26575800
H	3.09222700	1.66227200	-3.03095000
H	1.98654900	3.03784600	-2.68374300
H	3.55986200	2.94313300	-1.85896300
C	5.37133900	1.43706500	1.95159900
C	5.37854100	1.27334200	3.33291700
C	4.25586100	0.75707700	3.97426900
C	3.12574800	0.41435600	3.24164500
C	3.12393100	0.58652300	1.86010900
C	4.24655600	1.09233100	1.20893700
H	6.25122200	1.82934000	1.44415800
H	4.25708300	0.62442000	5.05480800
H	2.23026500	0.02186900	3.72081900
H	4.23184300	1.18893900	0.12484100
O	2.01249000	0.21033700	1.13845800
H	0.27558400	0.70825400	0.30432900
H	6.26133300	1.54272400	3.90988300

Section 11: Cartesian coordinates of structures in Figure 6

Structure and coordinates of **TS-o9**

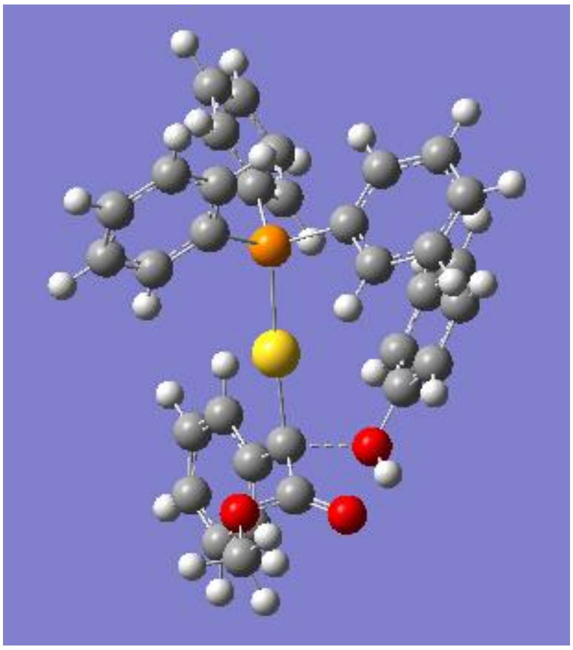

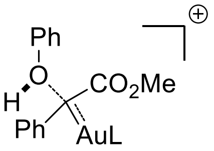

TS-o9

L = Ph_3P

C	2.22905400	2.06608300	5.24735400
C	2.43210200	1.77246900	3.91020200
C	1.40471500	1.97502800	2.96972000
C	0.16552200	2.46751300	3.41247800
C	-0.03545200	2.76551500	4.75212400
C	0.99557000	2.56521400	5.66717800
H	3.02745100	1.90882400	5.96896200
H	3.39276700	1.37320000	3.58269200
H	-0.63584500	2.61783100	2.68650800
H	-0.99393800	3.15582200	5.08678000
H	0.83780800	2.79916700	6.71831100
C	1.60518000	1.64464200	1.56907600
C	3.00228700	1.62139500	1.03890400
O	3.55756000	0.63324000	0.58138200
O	3.52831500	2.83103700	1.06754100
C	4.83406300	2.96545700	0.48209400
H	5.09498400	4.01773600	0.59390300
H	5.54985100	2.32880000	1.00995700
H	4.80332100	2.68182200	-0.57400100
C	-0.84395100	-0.88480200	1.67925700
C	0.32157500	-0.73918200	0.94050300
C	0.38072200	-1.02156500	-0.41878000
C	-0.77605900	-1.45611100	-1.05833200
C	-1.96107100	-1.60026800	-0.34036000
C	-1.99173800	-1.32290800	1.02475100
H	-0.83404100	-0.66231900	2.74449200
H	1.31313900	-0.87967400	-0.96793200
H	-0.75257900	-1.66540200	-2.12717700
H	-2.91542000	-1.45057700	1.58680300
O	1.43496400	-0.23337900	1.60688800
H	2.27786400	-0.41995000	1.10630100
H	-2.86348900	-1.93407300	-0.84881000
Au	0.24852400	2.24575800	0.07024300
P	-1.39933100	2.79347700	-1.63252800
C	-1.46394400	1.44461300	-2.91107100
C	-2.65330900	0.79242200	-3.22771300

C	-0.27244400	1.08722400	-3.54636000
C	-2.64762700	-0.22441300	-4.18017100
H	-3.58205500	1.07222800	-2.73043900
C	-0.27363700	0.07754800	-4.50084800
H	0.65621500	1.60267300	-3.29342100
C	-1.46181900	-0.58173100	-4.81462000
H	-3.57583200	-0.73468300	-4.43041400
H	0.65243500	-0.19651300	-5.00256400
H	-1.46198100	-1.37380600	-5.56087200
C	-3.09786000	2.87318100	-0.89167900
C	-4.06449300	3.75301300	-1.37571300
C	-3.40399800	1.98639300	0.14303100
C	-5.34015500	3.74525500	-0.81857400
H	-3.82354500	4.44676400	-2.18081200
C	-4.68257500	1.97773500	0.68803400
H	-2.64283000	1.29504300	0.51347000
C	-5.64867200	2.85950300	0.20918100
H	-6.09475100	4.43549100	-1.19022100
H	-4.92425600	1.28602000	1.49322600
H	-6.64735100	2.85714200	0.64139200
C	-1.12662900	4.36994300	-2.56315600
C	-1.60816500	4.51912100	-3.86493700
C	-0.47494500	5.42561900	-1.92480300
C	-1.43859600	5.73220300	-4.52358400
H	-2.11244700	3.68996100	-4.36247700
C	-0.30918900	6.63599500	-2.58954800
H	-0.09646900	5.29880200	-0.90932000
C	-0.79142600	6.78761900	-3.88622400
H	-1.81257000	5.85315500	-5.53824700
H	0.20024400	7.46041400	-2.09487600
H	-0.65914700	7.73471900	-4.40558900

Structure and coordinates of **Ts-c9**

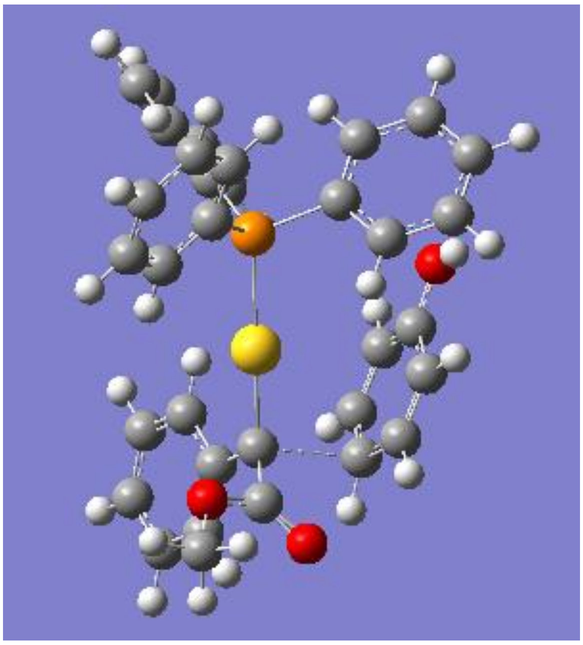

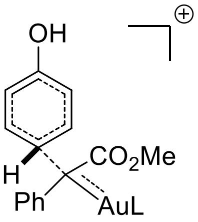

TS-c9

L = Ph$_3$P

Au	-3.49552600	1.50267700	2.94750000
P	-2.87658800	3.26596600	4.51256400
C	-3.62436900	4.89267500	3.99644500
C	-4.38081300	5.66590000	4.87492100
C	-3.43377200	5.31227500	2.67768100
C	-4.96108100	6.85142300	4.42965200
H	-4.52962300	5.33819200	5.90362900
C	-4.01271900	6.49894800	2.23622600
H	-2.83474900	4.70486000	1.99601700
C	-4.78319600	7.26613800	3.11278500
H	-5.55677900	7.45188600	5.11394300
H	-3.85497300	6.83293600	1.21162700
H	-5.23681100	8.19303000	2.76722100
C	-3.57452800	2.92914700	6.19854200
C	-2.85212200	3.18892600	7.36119300
C	-4.88045400	2.43730900	6.27509000
C	-3.43874800	2.95153700	8.60182600
H	-1.83310700	3.57054600	7.30333900
C	-5.46287100	2.21147700	7.51606800
H	-5.44558800	2.24587200	5.35926000
C	-4.73951800	2.46557100	8.67941600
H	-2.87486800	3.15035100	9.51094100
H	-6.48176000	1.83422200	7.57750100
H	-5.19404500	2.28444600	9.65141200
C	-1.06407400	3.58242300	4.70809500
C	-0.60068600	4.83122900	5.12674300
C	-0.16849800	2.53991200	4.46702100
C	0.76328400	5.03266500	5.30482500
H	-1.30428500	5.64370600	5.31126100
C	1.19455400	2.74809500	4.64930400
H	-0.53876200	1.56892100	4.13457800
C	1.65802500	3.99233000	5.06647600
H	1.12924600	6.00440100	5.63001800
H	1.89669300	1.93858500	4.46106200
H	2.72524200	4.15426700	5.20448600
C	-5.25294300	-3.62181300	1.94177700
C	-4.76059100	-2.44080900	1.41132800

C	-4.65991000	-1.27922900	2.20559000
C	-5.09006600	-1.34732300	3.54654400
C	-5.57887900	-2.52939600	4.07606000
C	-5.66081800	-3.66790900	3.27443700
H	-5.31904800	-4.51132500	1.31936100
H	-4.45297100	-2.40526300	0.36562300
H	-5.02703000	-0.45097600	4.16591600
H	-5.89491200	-2.57064700	5.11604200
H	-6.04658700	-4.59683800	3.69009500
C	-4.10468500	-0.04603300	1.67046300
C	-3.22220900	-0.25434200	0.47073600
O	-3.50173700	-0.14559700	-0.70050600
O	-2.01619100	-0.61667500	0.92159200
C	-1.05004900	-0.92255400	-0.09086600
H	-0.14961500	-1.22476400	0.44503400
H	-1.41085600	-1.73568600	-0.72892400
H	-0.85497000	-0.04122200	-0.70965300
C	-6.80600100	0.86851100	1.66161000
C	-5.86997900	0.81119800	0.59793700
C	-5.32136400	2.02671900	0.12438200
C	-5.55198900	3.21184000	0.78836600
C	-6.39055800	3.22015700	1.91638700
C	-7.04797400	2.04315900	2.32881600
H	-7.30209400	-0.04665200	1.98227200
H	-4.68377100	2.00584400	-0.75592800
H	-5.10600400	4.14390800	0.44212200
H	-7.73440600	2.10363900	3.17050200
O	-6.61462800	4.31523600	2.63991100
H	-6.04900700	5.05276000	2.34289100
H	-5.84630500	-0.05508300	-0.05967200

Structure and coordinates of **Int-o10**

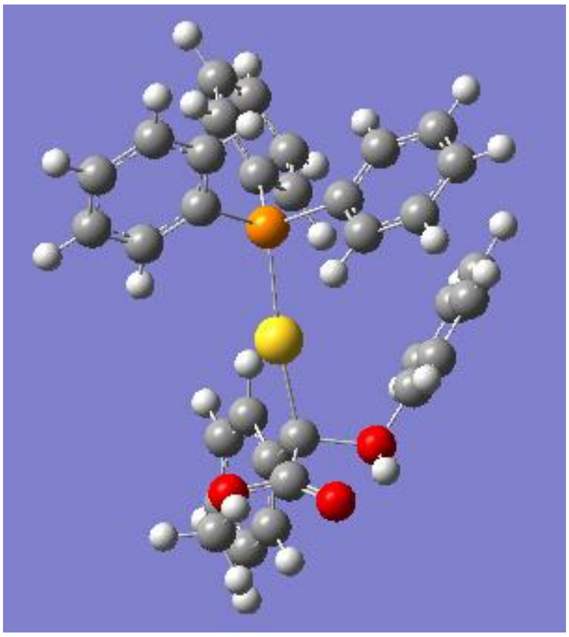

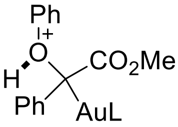

Int-o10
L = Ph_3P

C	2.12317200	1.93953400	5.25360900
C	2.34762400	1.64716300	3.91675700
C	1.33959300	1.84417400	2.96272200
C	0.09738400	2.33015000	3.38255600
C	-0.12877200	2.62504100	4.72246300
C	0.88323900	2.43115900	5.65698400
H	2.91400000	1.78620300	5.98483700
H	3.31765100	1.25545200	3.60482700
H	-0.69646100	2.47942100	2.64752000
H	-1.09642400	3.01000700	5.03744100
H	0.70641500	2.66410300	6.70520200
C	1.58679300	1.48255100	1.54230600
C	2.97359700	1.68950100	1.03047600
O	3.62045500	0.76654200	0.53717400
O	3.39332800	2.92953500	1.12584400
C	4.69930400	3.19920300	0.58993800
H	4.86779800	4.26278800	0.75817900
H	5.45127400	2.60009000	1.11150200
H	4.72165600	2.96507400	-0.47845700
C	-0.65413600	-0.97270700	1.64905900
C	0.45592500	-0.72904200	0.86130000
C	0.50611900	-0.99132700	-0.49821700
C	-0.63353600	-1.51487400	-1.10118500
C	-1.77425000	-1.76242100	-0.34070800
C	-1.78342900	-1.50010000	1.02748000
H	-0.62600000	-0.74774100	2.71315500
H	1.40026300	-0.75545300	-1.07716800
H	-0.63333800	-1.70857600	-2.17279800
H	-2.67227100	-1.71078900	1.61850800
O	1.56888700	-0.11476000	1.49291600
H	2.44810900	-0.28029500	1.00007300
H	-2.66366800	-2.16450700	-0.82191600
Au	0.22869800	2.21793400	0.05960800
P	-1.41589300	2.81143600	-1.62400200
C	-1.53730100	1.43085800	-2.86620700
C	-2.73605300	0.76358000	-3.10658400
C	-0.37802000	1.06790000	-3.55637500

C	-2.77329000	-0.27046000	-4.04020600
H	-3.63991600	1.04630000	-2.56720700
C	-0.42278600	0.04253000	-4.49318500
H	0.55837200	1.59638500	-3.36621200
C	-1.62154200	-0.62900800	-4.73334100
H	-3.71058700	-0.78919700	-4.23272400
H	0.47661800	-0.23190800	-5.04129100
H	-1.65705300	-1.43074700	-5.46843200
C	-3.10497800	2.96988200	-0.87401800
C	-4.03726400	3.88445400	-1.36071200
C	-3.44008500	2.11223300	0.17675300
C	-5.30550500	3.94218900	-0.78986400
H	-3.77491200	4.55542400	-2.17830600
C	-4.71090600	2.16951500	0.73629500
H	-2.70561700	1.39416500	0.55041700
C	-5.64161900	3.08716600	0.25501200
H	-6.03268400	4.65984000	-1.16431500
H	-4.97416100	1.50287600	1.55530600
H	-6.63360300	3.13833300	0.69940700
C	-1.09298600	4.35012900	-2.60000900
C	-1.56756700	4.47781000	-3.90660500
C	-0.40692300	5.40112500	-1.99029200
C	-1.35466700	5.66424200	-4.60021000
H	-2.09853600	3.65198000	-4.38129100
C	-0.19918100	6.58522000	-2.68967300
H	-0.03518800	5.29085200	-0.97043400
C	-0.67285600	6.71498700	-3.99189200
H	-1.72169000	5.76808800	-5.61927800
H	0.33606000	7.40630600	-2.21725300
H	-0.50607900	7.64095600	-4.53869400

Structure and coordinates of **Int-c10**

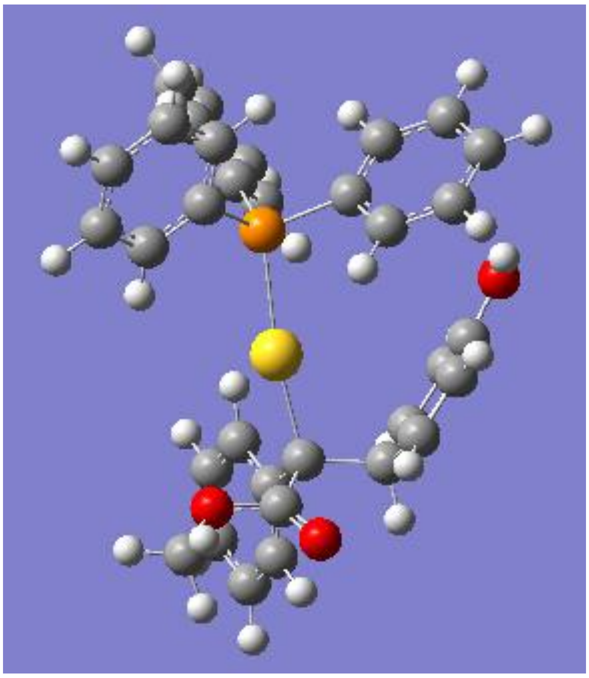

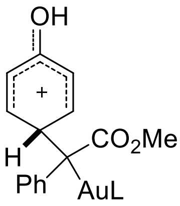

Int-c10
L = Ph_3P

Au	-3.44437900	1.47084800	2.96291800
P	-2.82162800	3.22204400	4.54933100
C	-3.67002500	4.79976000	4.01187800
C	-4.53652800	5.49764600	4.85229700
C	-3.44518800	5.25548700	2.71026300
C	-5.18618700	6.64012600	4.39013100
H	-4.71087000	5.14600400	5.86901500
C	-4.09064400	6.40165300	2.25074700
H	-2.76173200	4.71133800	2.05506900
C	-4.96630400	7.09447700	3.09166500
H	-5.86049900	7.18225100	5.04981400
H	-3.89141400	6.77177600	1.24531400
H	-5.46107300	7.99758300	2.73835700
C	-3.48005400	2.88783900	6.25145000
C	-2.78185900	3.25830700	7.39935200
C	-4.73022900	2.27178300	6.35510700
C	-3.33707100	3.00903200	8.65162000
H	-1.80384900	3.73217600	7.31907300
C	-5.28355900	2.03549900	7.60752000
H	-5.27166100	1.98385500	5.45024600
C	-4.58410000	2.40135800	8.75539300
H	-2.79005300	3.29045300	9.54914400
H	-6.25784500	1.55786800	7.69083700
H	-5.01407000	2.20946900	9.73644700
C	-1.02933600	3.64817700	4.71438400
C	-0.61799000	4.92857200	5.09041900
C	-0.09035000	2.63882600	4.49710800
C	0.73748200	5.19330200	5.25158900
H	-1.35494900	5.71560700	5.25593000
C	1.26328800	2.91022300	4.66352700
H	-0.41982800	1.64311600	4.19700300
C	1.67503800	4.18520400	5.03971200
H	1.06318800	6.18974400	5.54326100
H	1.99827500	2.12593000	4.49503600
H	2.73491900	4.39701400	5.16614500
C	-5.60894300	-3.45958400	1.77093000

C	-5.12000000	-2.25358900	1.28420900
C	-4.77796100	-1.20617800	2.15010300
C	-4.95764800	-1.40187700	3.52191600
C	-5.45063800	-2.60732500	4.01365200
C	-5.77705800	-3.63947700	3.14107100
H	-5.85626400	-4.26225700	1.07872200
H	-4.98372000	-2.12878100	0.20693200
H	-4.69610900	-0.60155300	4.21740800
H	-5.57231300	-2.74104400	5.08711100
H	-6.15832300	-4.58287400	3.52687400
C	-4.26823300	0.09145300	1.55096400
C	-3.20944100	-0.20865800	0.52698900
O	-3.25288300	0.09089000	-0.65133300
O	-2.18926000	-0.88288900	1.06924200
C	-1.17746500	-1.30030200	0.15288200
H	-0.44555100	-1.84743900	0.74877200
H	-1.60450100	-1.94998700	-0.61849900
H	-0.71202000	-0.43487600	-0.33017900
C	-6.60720900	0.97202400	1.73778700
C	-5.47855800	0.84171100	0.80053600
C	-5.01215400	2.12325500	0.24358900
C	-5.44607600	3.32561000	0.70180000
C	-6.42332600	3.35819200	1.73019400
C	-7.05718600	2.16900000	2.18597700
H	-7.04472700	0.05046000	2.12040000
H	-4.26166400	2.05643300	-0.54181000
H	-5.05439600	4.26225700	0.30788200
H	-7.85446300	2.26489200	2.91912900
O	-6.79731100	4.47071600	2.30631600
H	-6.21566600	5.22733700	2.07303200
H	-5.75386300	0.17578800	-0.04078400

Structure and coordinates of **TS-o11**

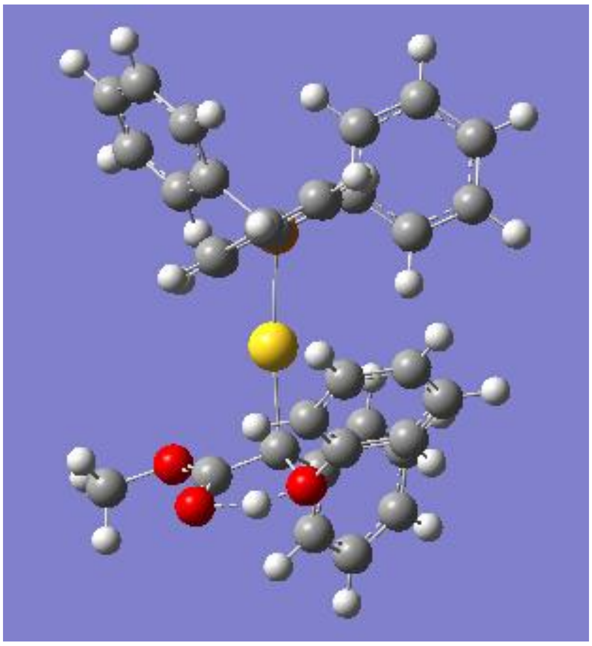

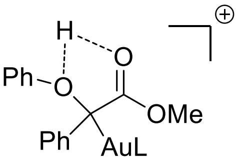

TS-o11
L = Ph_3P

C	2.08047500	1.95688600	5.26558800
C	2.31156500	1.64886000	3.93295500
C	1.31579800	1.85226700	2.96863800
C	0.07772200	2.35932200	3.37294900
C	-0.15615300	2.66764000	4.70881400
C	0.84442900	2.46869600	5.65433800
H	2.86314800	1.79824000	6.00450600
H	3.27760500	1.23729500	3.63404500
H	-0.70920400	2.51133800	2.63095000
H	-1.12199200	3.06677200	5.01158500
H	0.66128500	2.71228900	6.69901300
C	1.57355700	1.46142200	1.55346700
C	2.94587900	1.67724200	1.03980700
O	3.54548400	0.69961300	0.54429900
O	3.43886000	2.87704000	1.11615000
C	4.75278500	3.07849700	0.56006800
H	5.01302800	4.10830300	0.80290300
H	5.46173600	2.37810100	1.00981300
H	4.72130300	2.93048800	-0.52320500
C	-0.63622700	-0.94965100	1.65239700
C	0.47101900	-0.69650100	0.86058400
C	0.50986500	-0.97277400	-0.49789000
C	-0.62803000	-1.51068800	-1.09165600
C	-1.76199000	-1.76484200	-0.32384400
C	-1.76416200	-1.49271800	1.04256700
H	-0.60520300	-0.71744900	2.71504400
H	1.39833900	-0.73943000	-1.08587100
H	-0.63036100	-1.71423800	-2.16154400
H	-2.64698600	-1.70836200	1.64105900
O	1.58149900	-0.07996500	1.47560700
H	2.56612000	-0.13135500	0.92086800
H	-2.64968400	-2.18168100	-0.79563400
Au	0.23302500	2.23253300	0.05616100
P	-1.41399600	2.82057200	-1.62598700
C	-1.52673500	1.43932600	-2.86773100
C	-2.72145600	0.76475200	-3.10771200
C	-0.36518100	1.08253600	-3.55717100

C	-2.75224500	-0.27015200	-4.04049400
H	-3.62692500	1.04192700	-2.56827500
C	-0.40335500	0.05575300	-4.49252400
H	0.56802100	1.61651400	-3.36677000
C	-1.59808700	-0.62284600	-4.73254300
H	-3.68627600	-0.79484300	-4.23259100
H	0.49799000	-0.21459400	-5.03944900
H	-1.62837100	-1.42600300	-5.46630400
C	-3.10198000	2.96556700	-0.87130300
C	-4.03829700	3.88110000	-1.34831000
C	-3.43154000	2.09705400	0.17229000
C	-5.30565000	3.92904200	-0.77453500
H	-3.77994100	4.56011600	-2.16048300
C	-4.70154900	2.14484400	0.73462500
H	-2.69443600	1.37725800	0.53738400
C	-5.63646500	3.06346900	0.26325400
H	-6.03628600	4.64708200	-1.14140700
H	-4.96084700	1.46934200	1.54759900
H	-6.62789300	3.10668000	0.70972500
C	-1.10501600	4.36373000	-2.59923700
C	-1.57980800	4.48904200	-3.90593500
C	-0.43115900	5.42085800	-1.98655600
C	-1.37927200	5.67938400	-4.59665200
H	-2.10193800	3.65873800	-4.38259000
C	-0.23579200	6.60877100	-2.68296000
H	-0.05979400	5.31229500	-0.96639100
C	-0.70963600	6.73621700	-3.98538600
H	-1.74675500	5.78162700	-5.61571600
H	0.28976500	7.43472200	-2.20810300
H	-0.55302800	7.66540300	-4.52972900

Structure and coordinates of **TS-c11**

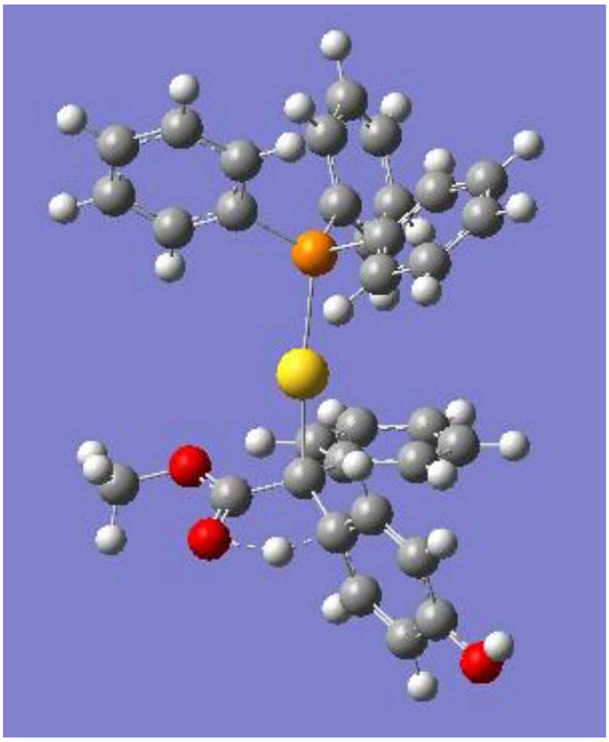

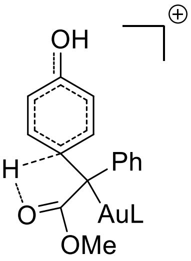

TS-c11
L = Ph_3P

Au	-5.02469400	3.23613500	1.66856200
P	-4.95756200	5.47171800	2.59472500
C	-5.31929200	6.84890700	1.40600100
C	-6.03287500	7.98010300	1.80114600
C	-4.82272700	6.74649800	0.10548100
C	-6.25182000	9.00776200	0.88833900
H	-6.42003700	8.06050300	2.81696400
C	-5.03992500	7.77901300	-0.79878400
H	-4.26449100	5.85840900	-0.19568600
C	-5.75709400	8.90704100	-0.40807700
H	-6.81231500	9.88954100	1.19244600
H	-4.65314600	7.70130300	-1.81292800
H	-5.93189200	9.71106500	-1.12020500
C	-6.14925700	5.68606200	4.00190000
C	-5.73479100	6.16587400	5.24206900
C	-7.49335600	5.37559600	3.77897600
C	-6.66870900	6.33332100	6.26184400
H	-4.68642100	6.40640400	5.41612900
C	-8.42136300	5.55583100	4.79696700
H	-7.81392800	5.00712700	2.80205400
C	-8.00780600	6.03246600	6.03947100
H	-6.34515200	6.70503100	7.23206400
H	-9.47055400	5.32536900	4.62157600
H	-8.73578900	6.17036800	6.83654700
C	-3.25938600	5.78584900	3.27151500
C	-2.77905200	7.08973900	3.40274300
C	-2.47452000	4.70083400	3.66688300
C	-1.51131700	7.30522600	3.93210200
H	-3.39275300	7.93451500	3.08811400
C	-1.20801900	4.92439500	4.19651700
H	-2.84790500	3.67913800	3.55976900
C	-0.72746300	6.22407700	4.32672300
H	-1.13198300	8.32016000	4.03253700
H	-0.59405800	4.07823800	4.49860800

H	0.26621000	6.39599600	4.73602900
C	-1.11412300	0.83132200	2.22468000
C	-2.21840600	1.04693900	1.40518300
C	-3.51920000	0.88179200	1.90025000
C	-3.66321500	0.50309000	3.24626900
C	-2.55827900	0.29050000	4.06014300
C	-1.27224000	0.45358500	3.55346000
H	-0.11583800	0.95816600	1.80892900
H	-2.06169600	1.35080900	0.37464100
H	-4.66308600	0.38195900	3.66822500
H	-2.70604300	-0.00525900	5.09737900
H	-0.40407800	0.28163900	4.18632700
C	-4.76850000	1.14953800	1.09921700
C	-4.77434800	1.21633300	-0.37208300
O	-5.77747000	0.79316000	-0.98688200
O	-3.75759100	1.75442100	-1.01020600
C	-3.88497300	1.86341500	-2.43632400
H	-2.95251800	2.31362000	-2.77814300
H	-4.02293700	0.87399200	-2.88177900
H	-4.73914200	2.49892300	-2.68888400
C	-5.68883400	-1.18745100	1.49097200
C	-5.95704100	0.23028800	1.43913100
C	-7.07321200	0.68227300	2.21870000
C	-7.90575500	-0.18842800	2.87457300
C	-7.62690400	-1.56833500	2.84146500
C	-6.51048200	-2.06599900	2.13972200
H	-4.81461700	-1.55686600	0.95389100
H	-7.27628900	1.75441000	2.24968400
H	-8.77022600	0.17561200	3.42917300
H	-6.32823400	-3.13771900	2.14795100
O	-8.38707700	-2.46409000	3.45443700
H	-9.13583000	-2.04491600	3.90914300
H	-6.36685900	0.31379800	0.23664000

Structure and coordinates of **Int-o12**

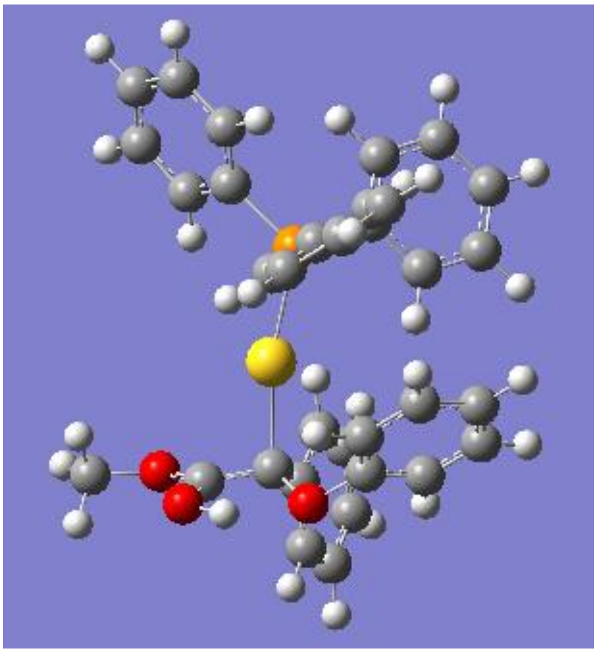

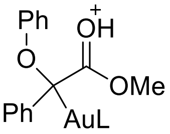

Int-o12
L = Ph_3P

C	1.53407100	1.48007100	5.15514100
C	1.88522300	1.14254400	3.85570300
C	1.22084500	1.71993200	2.76639500
C	0.19654600	2.63523100	3.00496000
C	-0.14968400	2.98140700	4.30852300
C	0.51574400	2.40380700	5.38306800
H	2.05634500	1.02327500	5.99344200
H	2.67213700	0.40942600	3.67534500
H	-0.34277100	3.08060500	2.16631800
H	-0.94863200	3.69958100	4.48121300
H	0.24077400	2.66997200	6.40176600
C	1.62918900	1.27534700	1.39219200
C	2.90732400	1.70519100	0.91168200
O	3.62441900	0.93662900	0.13772800
O	3.38110500	2.86522600	1.22795400
C	4.56311100	3.34137400	0.55387300
H	4.76779700	4.31480200	0.99793600
H	5.39635100	2.65422000	0.72141000
H	4.35878500	3.43919200	-0.51740500
C	-0.61056500	-0.85843500	1.87228700
C	0.34947700	-0.66765700	0.88553800
C	0.09399700	-0.98655700	-0.44459000
C	-1.15702900	-1.48042500	-0.80140600
C	-2.13440400	-1.66477700	0.17369300
C	-1.85659600	-1.36154000	1.50514900
H	-0.37577300	-0.61745600	2.90724900
H	0.87532100	-0.84151300	-1.19193100
H	-1.36310300	-1.71896000	-1.84341100
H	-2.61452000	-1.52114100	2.27008700
O	1.59589700	-0.14386500	1.21237800
H	3.15052600	0.07065100	0.11456000
H	-3.11194000	-2.05453300	-0.10302900
Au	0.41435300	2.18474700	-0.22222800
P	-1.27858300	2.84391500	-1.82790200
C	-1.47505200	1.54933500	-3.14478300
C	-2.73135500	1.06624400	-3.50775000
C	-0.32580500	1.07687400	-3.78209900

C	-2.83407800	0.10851300	-4.51375400
H	-3.62717700	1.43445200	-3.00751800
C	-0.43511100	0.12249400	-4.78623400
H	0.65466900	1.45898100	-3.49122200
C	-1.68993700	-0.36181800	-5.15089000
H	-3.81333800	-0.26898300	-4.80142400
H	0.45900700	-0.24452900	-5.28633300
H	-1.77578300	-1.11024100	-5.93620800
C	-2.89869900	2.91331100	-0.92716100
C	-3.86136700	3.87573100	-1.22663800
C	-3.14381000	1.94407300	0.04869100
C	-5.07356200	3.86703600	-0.54183200
H	-3.66732700	4.63281000	-1.98648800
C	-4.35997600	1.93643300	0.72055400
H	-2.38677000	1.18932000	0.27657500
C	-5.32214900	2.90055000	0.42806200
H	-5.82589800	4.62035700	-0.76642700
H	-4.55250900	1.17968100	1.47934400
H	-6.27068600	2.89959600	0.96149600
C	-1.06248800	4.46307200	-2.69659400
C	-1.59873400	4.66506600	-3.96914100
C	-0.40580800	5.49945100	-2.03227400
C	-1.47413000	5.91117300	-4.57462400
H	-2.11149200	3.85256600	-4.48481700
C	-0.28777300	6.74312600	-2.64244800
H	0.01264700	5.33120700	-1.03854100
C	-0.82100400	6.94688100	-3.91235500
H	-1.88888800	6.07259900	-5.56759300
H	0.22450300	7.55349800	-2.12782200
H	-0.72393200	7.91958200	-4.39057600

Structure and coordinates of **Int-c12**

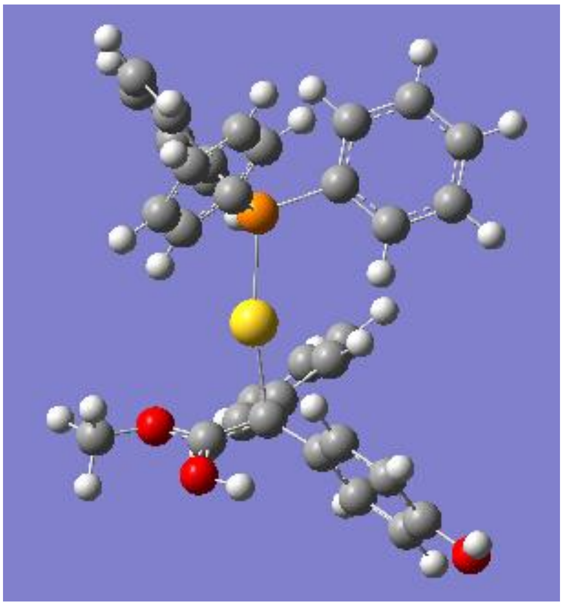

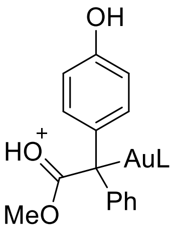

Int-c12
L = Ph_3P

Au	-4.70025600	3.19883800	1.64472100
P	-4.81765200	5.44858300	2.49661500
C	-5.72859900	6.67083500	1.44688300
C	-6.48177500	7.69129400	2.02724600
C	-5.59919000	6.58888400	0.05947600
C	-7.10688400	8.63019200	1.21261700
H	-6.58372200	7.75269200	3.11063600
C	-6.22245600	7.53304700	-0.74789200
H	-5.01038400	5.78566200	-0.38661400
C	-6.97661400	8.55169200	-0.17075000
H	-7.69811900	9.42551800	1.66181700
H	-6.12405700	7.47123500	-1.82971800
H	-7.46795600	9.28748700	-0.80412700
C	-5.64774000	5.41019200	4.15510700
C	-5.26531300	6.30026100	5.15955000
C	-6.67568300	4.49234200	4.37896200
C	-5.91858900	6.27214300	6.38744100
H	-4.45691600	7.01107300	4.98533000
C	-7.32510700	4.47080600	5.60830300
H	-6.96730200	3.79340100	3.59141900
C	-6.94528200	5.35939100	6.61076400
H	-5.62108800	6.96311000	7.17352500
H	-8.12448800	3.75433500	5.78599700
H	-7.45079400	5.33713200	7.57415200
C	-3.12035300	6.13431900	2.78918800
C	-2.81235700	7.46165000	2.49606700
C	-2.16107600	5.29023400	3.35349800
C	-1.53445700	7.94353800	2.76713100
H	-3.56290800	8.11852100	2.05646800
C	-0.89017700	5.78096600	3.62535100
H	-2.40971100	4.25128500	3.58341500
C	-0.57685700	7.10603800	3.32945600
H	-1.28850900	8.97816200	2.53645400
H	-0.14051000	5.12564000	4.06399900
H	0.42111100	7.48671900	3.53742100
C	-1.47937900	-0.32055400	3.19090400
C	-2.34427200	0.00065100	2.14879000
C	-3.55166700	0.65735100	2.40319400

C	-3.87734200	0.95745600	3.73624000
C	-3.01277200	0.63729900	4.77407200
C	-1.80468100	-0.00024700	4.50388600
H	-0.54623900	-0.83560100	2.97024600
H	-2.07125200	-0.26938800	1.13162600
H	-4.83529200	1.43274400	3.95961900
H	-3.28735000	0.88090100	5.79864000
H	-1.12618100	-0.25596900	5.31509100
C	-4.56085600	0.98496900	1.33357500
C	-4.15472000	1.12599400	-0.02112700
O	-4.98596200	0.99808700	-1.03330400
O	-2.91920700	1.39298300	-0.35131500
C	-2.63745800	1.82658900	-1.69168500
H	-1.56760100	2.03435200	-1.70372100
H	-2.88635700	1.04219300	-2.41126500
H	-3.20725000	2.73610600	-1.91442400
C	-5.99960400	-0.97811000	1.96339200
C	-5.91876900	0.34846100	1.50730100
C	-7.11881800	0.99929000	1.19208400
C	-8.34853300	0.35602200	1.30985100
C	-8.40154000	-0.95952600	1.76638200
C	-7.21441900	-1.62307400	2.09574800
H	-5.08327600	-1.50820700	2.22174700
H	-7.10117500	2.04318000	0.86566400
H	-9.26737400	0.88851600	1.06264500
H	-7.27471900	-2.64965500	2.45019600
O	-9.55025800	-1.64522500	1.91811800
H	-10.30670800	-1.09783900	1.66221100
H	-5.85748800	0.70316400	-0.68928400

Structure and coordinates of **Int-o13**

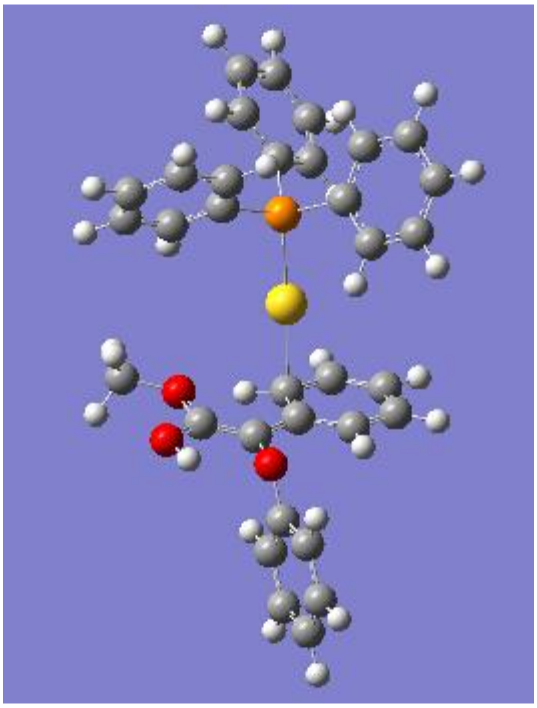

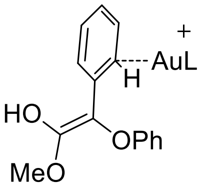

Int-o13
L = Ph_3P

C	1.62241100	0.69281500	2.66451500
C	1.47065500	2.08718100	2.42198400
C	1.65229200	2.60369200	1.09414600
C	1.90720200	1.67283400	0.06553900
C	2.01703000	0.31798500	0.33335100
C	1.87243200	-0.19139100	1.62750900
H	1.56122200	0.33898900	3.69275800
H	1.52129900	2.77240300	3.26786300
H	2.04246300	2.03612900	-0.95003200
H	2.22974200	-0.36346600	-0.48856100
H	1.97732300	-1.25592700	1.81993500
C	1.60654700	4.00683000	0.77888900
C	1.16526000	5.03752300	1.56272200
O	1.10105400	6.28304100	1.10843000
O	0.73246600	4.85827700	2.80155000
C	0.20598800	5.98994600	3.50295500
H	-0.11324500	5.60273300	4.47232800
H	0.97721000	6.75490500	3.63646600
H	-0.64539300	6.41628900	2.96249000
C	5.57814800	4.55546300	-0.51443100
C	5.80946700	4.71328400	-1.87583200
C	4.73110300	4.75516900	-2.75688800
C	3.43112200	4.63821900	-2.28201600
C	3.21941500	4.48502100	-0.91619300
C	4.28022300	4.44039600	-0.02102000
H	6.41499200	4.52189500	0.18063200
H	4.90166000	4.87790500	-3.82467900
H	2.57187400	4.66581500	-2.94973100
H	4.09893100	4.31921900	1.04510800
O	1.88515000	4.39717400	-0.52571700
H	1.36500100	6.25774400	0.16796200
Au	-0.77335200	1.84277900	2.32382600
P	-3.16594200	1.73169300	2.12496300
C	-3.66523200	0.79763300	0.60507500
C	-4.86173200	0.08014900	0.57462400

C	-2.84422200	0.85197200	-0.52246900
C	-5.23358000	-0.58322700	-0.59014800
H	-5.49923200	0.03510800	1.45795300
C	-3.22453600	0.18920500	-1.68367900
H	-1.90592700	1.40992900	-0.49204400
C	-4.41683200	-0.52895000	-1.71605500
H	-6.16279100	-1.14872700	-0.61561200
H	-2.58530500	0.22697900	-2.56331300
H	-4.70882500	-1.05450600	-2.62301800
C	-3.97910100	0.89167300	3.56296800
C	-5.16324400	1.38287300	4.11109100
C	-3.40258200	-0.28079900	4.05480800
C	-5.76736500	0.69637200	5.16074800
H	-5.61195300	2.29753500	3.72380100
C	-4.01439300	-0.96271100	5.09899000
H	-2.47735100	-0.66088100	3.61810600
C	-5.19483200	-0.47218600	5.65277800
H	-6.68999100	1.07787200	5.59347400
H	-3.56827000	-1.87699700	5.48490600
H	-5.67083600	-1.00414300	6.47404600
C	-3.91583500	3.42314400	2.00437400
C	-4.92164800	3.70294200	1.08183200
C	-3.47943300	4.40492300	2.89690400
C	-5.48936800	4.97427000	1.05147900
H	-5.25921100	2.93500000	0.38618800
C	-4.05651900	5.66840400	2.86636900
H	-2.69642700	4.17368300	3.62239400
C	-5.05882000	5.95297000	1.94104600
H	-6.27198800	5.19752900	0.32938800
H	-3.72598700	6.43387400	3.56628200
H	-5.50628800	6.94447400	1.91409700
H	6.82621900	4.80303600	-2.25147400

Structure and coordinates of **Int-c13**

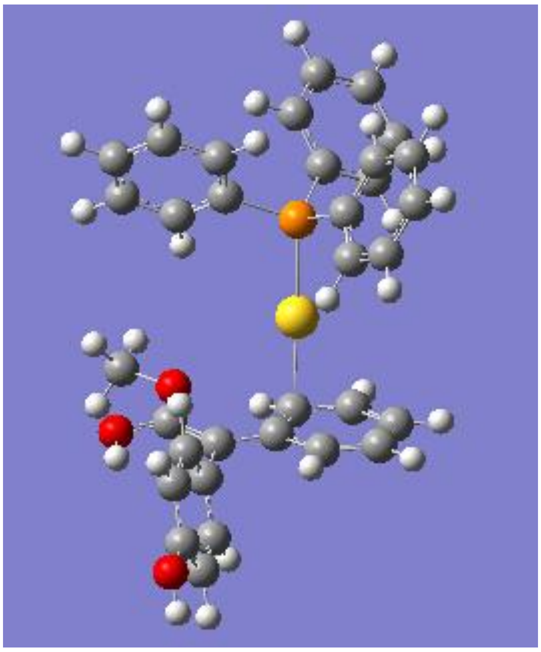

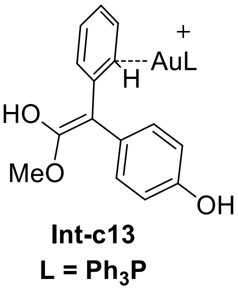

C	1.58444500	0.77656100	2.62111300
C	1.43824800	2.17577400	2.39941000
C	1.59001200	2.72218800	1.07765400
C	1.78503700	1.78725600	0.03439600
C	1.88780400	0.42733100	0.27710000
C	1.79093000	-0.09948300	1.56853800
H	1.55055700	0.41320200	3.64739900
H	1.51025500	2.84169200	3.25755800
H	1.88703500	2.15369000	-0.98460200
H	2.05556600	-0.24412600	-0.56347600
H	1.89131000	-1.16737500	1.74420300
C	1.58464000	4.14061400	0.77256800
C	1.19547400	5.12591100	1.64304100
O	1.22041200	6.42390200	1.34435300
O	0.72630600	4.87514500	2.86035000
C	0.40434000	5.97676500	3.71340000
H	0.04727000	5.52652100	4.64175100
H	1.29070900	6.58853000	3.90813300
H	-0.37710600	6.60017800	3.26709900
C	1.04245900	5.07714800	-1.50495600
C	1.99160000	4.59945300	-0.58749500
C	3.33205300	4.56301800	-0.99122900
C	3.71428300	4.98130700	-2.25785200
C	2.75393600	5.45945000	-3.15282200
C	1.41182700	5.50968800	-2.76942600
H	-0.00900100	5.10334500	-1.21332800
H	4.08531900	4.19229900	-0.29554500
H	4.76364000	4.94642300	-2.55308600
H	0.68031300	5.88421800	-3.48211800
O	3.05638700	5.88644900	-4.39542000
H	4.00891700	5.81170000	-4.55122400
H	1.58079100	6.52469000	0.44250000
Au	-0.80547500	1.92495400	2.32045700
P	-3.19439900	1.74377800	2.13356600
C	-3.66725200	0.72760600	0.65577900
C	-4.81342800	-0.06768100	0.67612400
C	-2.87896400	0.80206600	-0.49375500

C	-5.16988000	-0.78650100	-0.46052800
H	-5.42441500	-0.12887300	1.57713500
C	-3.24287900	0.08253600	-1.62599200
H	-1.97869100	1.42032300	-0.50202700
C	-4.38659800	-0.71156500	-1.60835000
H	-6.06157500	-1.40995400	-0.44723000
H	-2.62867900	0.13596400	-2.52256800
H	-4.66627200	-1.27938700	-2.49350900
C	-3.98306100	0.92798600	3.59963200
C	-5.15182300	1.42954400	4.16947600
C	-3.39968700	-0.24176000	4.09103700
C	-5.73476900	0.75582700	5.23969100
H	-5.60604400	2.34202800	3.78399000
C	-3.99063600	-0.91112800	5.15483300
H	-2.48658400	-0.63061700	3.63671300
C	-5.15689100	-0.41055200	5.72977400
H	-6.64578000	1.14557700	5.68932200
H	-3.53992100	-1.82394100	5.53898000
H	-5.61735500	-0.93347300	6.56558500
C	-4.00067900	3.39954700	1.93065300
C	-5.15363000	3.54262800	1.15904200
C	-3.46001000	4.49628700	2.60343800
C	-5.76320500	4.78955400	1.06032900
H	-5.57257900	2.68532500	0.63179800
C	-4.07681000	5.73805700	2.50439700
H	-2.55608500	4.37301700	3.20344600
C	-5.22578600	5.88409500	1.73123700
H	-6.66026800	4.90535100	0.45571000
H	-3.65956200	6.59582800	3.02853200
H	-5.70358500	6.85834000	1.64917200

Structure and coordinates of **TS-o14-2w**

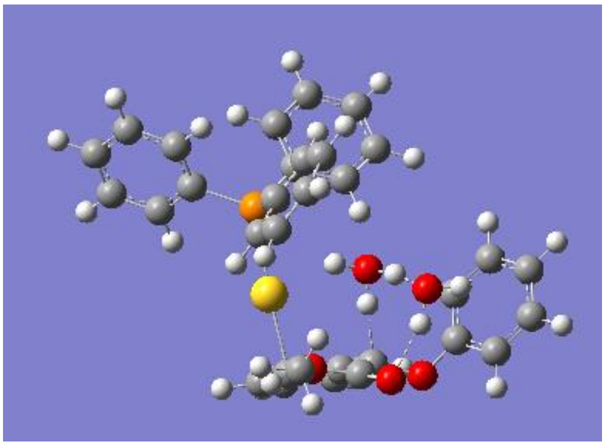

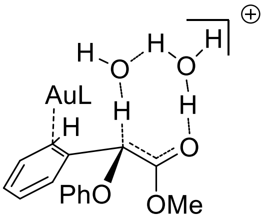

TS-o14-2w
L = Ph_3P

C	1.69694900	0.81884500	3.23535500
C	1.37054200	2.17380900	2.91963900
C	1.66692200	2.70132400	1.61030700
C	2.19056800	1.78551200	0.66803400
C	2.45228800	0.46841600	0.99861100
C	2.20746400	-0.03903800	2.28158800
H	1.55240400	0.48537100	4.26244200
H	1.24143100	2.87822900	3.74118600
H	2.44775500	2.15224400	-0.32204300
H	2.87970800	-0.18532500	0.23982500
H	2.44277500	-1.07141300	2.52658100
C	1.45565800	4.07661000	1.20163900
C	1.25809100	5.21828900	2.06758900
O	1.38348100	6.38987200	1.71636100
O	0.69129200	4.91183900	3.26644700
C	0.27099900	6.03694800	4.03307500
H	-0.12353200	5.63200900	4.96815600
H	1.10644600	6.71388300	4.23357700
H	-0.51124900	6.59621300	3.50037500
C	0.46500600	4.14911600	-1.62161500
C	1.48295400	4.89488500	-1.02657500
C	1.83697000	6.14049800	-1.54334300
C	1.14690800	6.65255800	-2.63873400
C	0.10194500	5.92862400	-3.21664800
C	-0.22829600	4.67402600	-2.70869900
H	0.21481900	3.16487100	-1.22240800
H	2.63789200	6.69094100	-1.05384700
H	1.42478700	7.62482800	-3.04222000
H	-1.02685800	4.09465600	-3.16960700
Au	-0.77871100	1.66958600	2.60657300
P	-3.16103500	1.61382100	2.24644200
C	-3.63755900	0.98612600	0.56971200
C	-4.68180200	0.07756300	0.41013000
C	-2.92358900	1.45114600	-0.53890200
C	-5.01107400	-0.36998400	-0.86669700
H	-5.23332900	-0.28649000	1.27627200
C	-3.26322800	1.00309200	-1.80851400
H	-2.11393700	2.17331500	-0.40901600
C	-4.30355600	0.08973100	-1.97169900
H	-5.82116000	-1.08479900	-0.99477400
H	-2.70936300	1.35870900	-2.67530700
H	-4.56111100	-0.26576600	-2.96726800
C	-4.14616300	0.70280300	3.51619900

C	-5.47494900	1.05622300	3.76069600
C	-3.55576400	-0.35915800	4.20138100
C	-6.21155900	0.33989100	4.69680200
H	-5.93096100	1.88908400	3.22396200
C	-4.29960800	-1.07102000	5.13625700
H	-2.51646200	-0.62526700	4.00453900
C	-5.62362700	-0.72031800	5.38302000
H	-7.24634100	0.61152100	4.89463300
H	-3.84190000	-1.89743900	5.67574100
H	-6.20215900	-1.27535100	6.11866500
C	-3.71860100	3.39156400	2.31066000
C	-4.57482400	3.92868200	1.35153200
C	-3.20305100	4.20670100	3.32697000
C	-4.89672200	5.28364400	1.39428800
H	-4.97728900	3.29670100	0.56042300
C	-3.53055300	5.55999700	3.36378100
H	-2.54430700	3.78118700	4.08785500
C	-4.36997600	6.09998100	2.39034200
H	-5.56024200	5.70283100	0.64074800
H	-3.13396400	6.19218900	4.15662400
H	-4.61725600	7.15906500	2.41301000
O	-0.76493400	6.90445300	0.15148500
H	0.06907300	7.01870700	0.67586300
H	-0.47718100	6.94761300	-0.78135100
O	-1.23237000	4.57700700	0.76710200
H	-0.21319300	4.24167800	0.91845600
H	-1.11386700	5.63421400	0.44720300
H	-1.70980400	4.54171200	1.62365800
O	2.18453300	4.42890200	0.04880000
H	-0.43712000	6.33361800	-4.07039500

Structure and coordinates of **Int-o14**

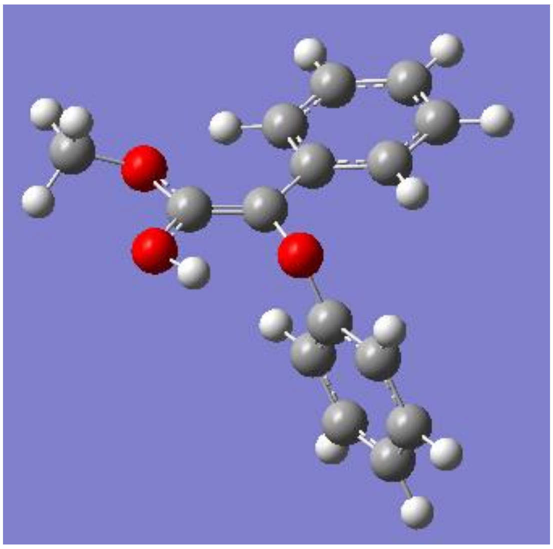

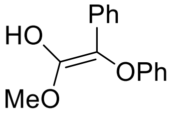

Int-o14

C	-3.16169900	-4.03289000	-8.48542900
C	-2.98202800	-2.93728700	-7.65282000
C	-3.83821200	-2.71171100	-6.56356200
C	-4.87336900	-3.63216600	-6.33368100
C	-5.04908300	-4.72135200	-7.17573000
C	-4.19736100	-4.93220300	-8.25581400
H	-2.48291600	-4.18284000	-9.32380700
H	-2.17256000	-2.23630900	-7.84729500
H	-5.53972100	-3.49039400	-5.48766200
H	-5.86001000	-5.42113000	-6.97756500
H	-4.33734400	-5.79123300	-8.90950500
C	-3.61164800	-1.54778300	-5.71555700
C	-4.41978800	-0.97637000	-4.79008900
O	-4.07342800	0.14791800	-4.13984900
O	-5.60797800	-1.47596500	-4.44456600
C	-6.55059800	-0.57856400	-3.87084100
H	-7.46750800	-1.15839200	-3.74629500
H	-6.20741400	-0.20995900	-2.89843300
H	-6.73889600	0.27345900	-4.53535300
C	-0.08508700	-0.74596100	-5.87892100
C	-1.25508600	-1.33732600	-5.40932900
C	-1.20904500	-2.36323600	-4.47236600
C	0.02972700	-2.79179000	-4.00385900
C	1.20628700	-2.20805900	-4.45933900
C	1.14214100	-1.18271800	-5.39963200
H	-0.16293200	0.04914800	-6.61841500
H	-2.13099200	-2.82222900	-4.12229200
H	0.06948200	-3.59703200	-3.27208300
H	2.05654900	-0.72092700	-5.76849500
O	-2.42930900	-0.82243700	-5.92009800
H	-3.24196900	0.44728700	-4.55095500
H	2.16888000	-2.55311200	-4.08742100

Structure and coordinates of **TS-c14-2w**

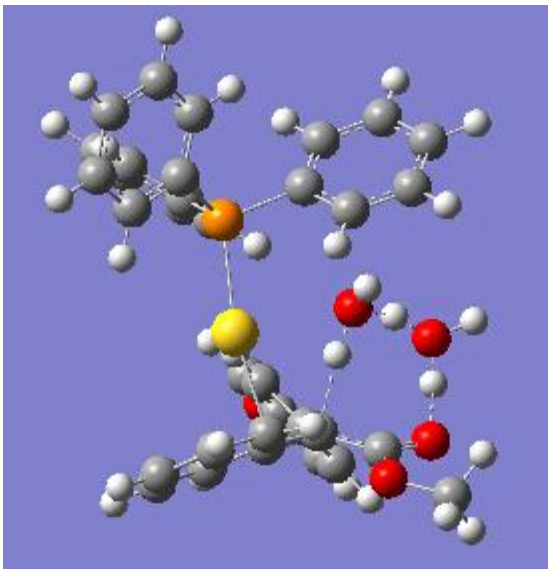

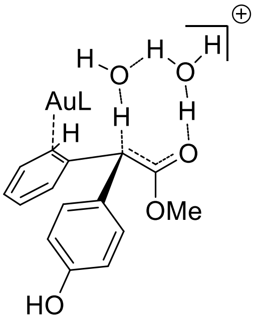

TS-c14-2w
L = Ph_3P

C	1.56932800	1.11695400	3.56024400
C	1.27229900	2.39522600	3.00237200
C	1.57768100	2.68831400	1.62340200
C	2.05685200	1.60181000	0.85808100
C	2.31084000	0.36142800	1.41925600
C	2.07072500	0.09693100	2.77299500
H	1.40849200	0.96917100	4.62762500
H	1.12727800	3.23224500	3.68747800
H	2.28601500	1.76694000	-0.19223200
H	2.72797900	-0.42149700	0.78767200
H	2.29117700	-0.87868700	3.19857300
C	1.36274000	4.00789400	1.02442500
C	1.76784900	5.20709300	1.71273200
O	1.61374400	6.36723800	1.28452500
O	2.31600200	5.03456900	2.92867200
C	2.68958400	6.21856200	3.62667300
H	3.08427800	5.88167600	4.58733800
H	3.45860100	6.76775000	3.07429800
H	1.82761000	6.87754600	3.77754700
C	0.36466400	3.30682400	-1.17369700
C	1.29817200	4.08685500	-0.47067900
C	2.13899100	4.90478600	-1.23447500
C	2.03692100	4.96474500	-2.61933800
C	1.09845000	4.18195400	-3.28936500
C	0.26515600	3.33786300	-2.55430800
H	-0.28994500	2.62857800	-0.62170000
H	2.88752800	5.51963000	-0.73827800
H	2.70476000	5.61566100	-3.18577500
H	-0.44934500	2.71205900	-3.08645100
O	0.94967700	4.19022900	-4.63408300
H	1.59564400	4.79280400	-5.02915100
Au	-0.87034200	1.83817900	2.62774100
P	-3.23550500	1.75138300	2.16889000
C	-3.63013600	1.35756700	0.39918300
C	-4.37553100	0.22859200	0.06672400
C	-3.13517500	2.20317600	-0.59815400
C	-4.62380900	-0.05586000	-1.27389600
H	-4.76186400	-0.42770800	0.84598400
C	-3.39644000	1.91559800	-1.93169800

H	-2.55962900	3.09122700	-0.33123400
C	-4.13704000	0.78407700	-2.26938400
H	-5.20439700	-0.93733700	-1.53812400
H	-3.02093600	2.57977800	-2.70845500
H	-4.33653100	0.55833600	-3.31503300
C	-4.21701700	0.58831800	3.21752100
C	-5.56407900	0.84350200	3.47865600
C	-3.61385600	-0.58357200	3.67498900
C	-6.30583500	-0.07931600	4.20694900
H	-6.03081600	1.75978800	3.11627000
C	-4.36476100	-1.50540800	4.39657800
H	-2.56031100	-0.77436000	3.46476900
C	-5.70706700	-1.25129400	4.66284600
H	-7.35487100	0.11679800	4.41894900
H	-3.89971000	-2.42092600	4.75597300
H	-6.29158500	-1.97082200	5.23255500
C	-3.89649700	3.46529500	2.47761200
C	-4.83051300	4.05326100	1.62650800
C	-3.41486400	4.17192000	3.58490300
C	-5.27109700	5.35132100	1.87463500
H	-5.20521700	3.50591400	0.76236000
C	-3.86003500	5.46820800	3.82863000
H	-2.69699800	3.70325600	4.26171300
C	-4.78452300	6.05922000	2.96856100
H	-5.99854700	5.80866600	1.20702500
H	-3.49157400	6.01398600	4.69520800
H	-5.13159000	7.07297500	3.15842100
O	-0.49238600	6.77871300	-0.12187000
H	-0.83559100	7.64526000	0.14558700
H	0.46120700	6.70471500	0.23944300
O	-1.24009100	4.81344400	1.03419000
H	-0.25910700	4.40108600	1.17951300
H	-1.00637000	5.84565200	0.46595200
H	-1.68564500	4.91640100	1.89464100

Structure and coordinates of **Int-c14**

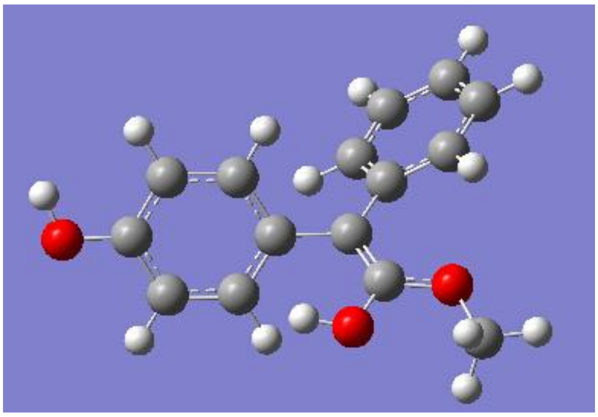

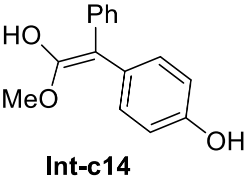

Int-c14

C	2.57205400	-3.63580300	0.98296100
C	2.60864400	-2.26890900	1.22599600
C	2.73217600	-1.35003000	0.17292600
C	2.83274800	-1.86646300	-1.12838400
C	2.79865600	-3.23311300	-1.36970600
C	2.66601700	-4.12944900	-0.31420300
H	2.47898200	-4.32462200	1.82193200
H	2.54590800	-1.90363600	2.24761700
H	2.93243600	-1.17503600	-1.96474300
H	2.87260200	-3.59982800	-2.39279000
H	2.63958300	-5.20191500	-0.49994400
C	2.79387400	0.10965700	0.37537800
C	2.14951000	0.75922800	1.37973800
O	2.24039200	2.08360100	1.61793500
O	1.35214000	0.13216800	2.25218200
C	0.65829000	0.90481000	3.21700900
H	0.06807900	0.18799900	3.79247900
H	1.35031900	1.43440300	3.88179600
H	-0.00800100	1.63478200	2.74245000
C	3.08700600	1.99110600	-1.29208100
C	3.61837500	0.91817100	-0.55922500
C	4.97364700	0.61825000	-0.75269200
C	5.76326500	1.35702700	-1.62026500
C	5.21517300	2.43335000	-2.32004500
C	3.87032000	2.75047200	-2.15261900
H	2.02398900	2.21878300	-1.19916400
H	5.40839100	-0.22010000	-0.20898900
H	6.81680200	1.10402500	-1.75320100
H	3.45161400	3.57881400	-2.72038500
O	5.94132900	3.19669100	-3.17651700
H	6.84632700	2.85743600	-3.21226400
H	2.87859900	2.45528500	0.98408400

Structure and coordinates of **TS-o15-2w**

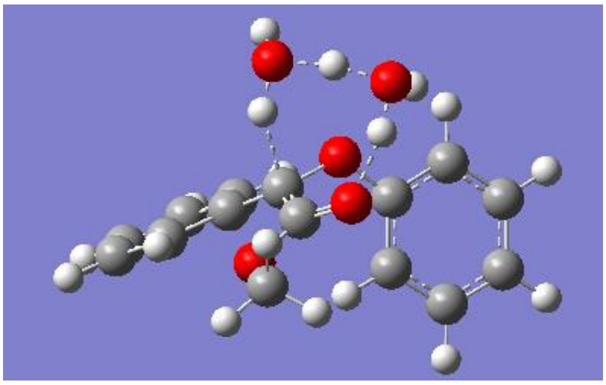

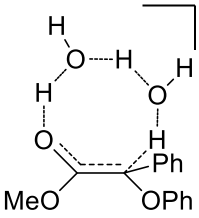

TS-o15-2w

C	-5.06022500	-4.47114900	-8.12674400
C	-4.64023800	-3.27160300	-7.56517500
C	-5.22939200	-2.76727100	-6.39348300
C	-6.27821300	-3.51484100	-5.82758900
C	-6.68952100	-4.71330200	-6.39268000
C	-6.08558700	-5.20698600	-7.54533000
H	-4.57340900	-4.83278300	-9.03198700
H	-3.83920400	-2.71396600	-8.04571800
H	-6.77247700	-3.14860000	-4.93294000
H	-7.50178400	-5.26716100	-5.92270900
H	-6.41348700	-6.14766400	-7.98417400
C	-4.79757500	-1.46592600	-5.86082500
C	-4.79868300	-1.04299500	-4.49837300
O	-4.27426500	0.00970000	-4.07187700
O	-5.47897600	-1.82715300	-3.64117400
C	-5.62004600	-1.32208600	-2.32045500
H	-6.18990900	-2.07823500	-1.77578900
H	-4.64214100	-1.17281900	-1.85114000
H	-6.15845100	-0.36758500	-2.31844600
C	-1.52533600	-0.41851600	-7.11653400
C	-2.46344200	-1.14334600	-6.38077100
C	-2.04369400	-2.11442400	-5.47493900
C	-0.68212800	-2.33606500	-5.30255800
C	0.26207500	-1.60790100	-6.01959400
C	-0.16791000	-0.64926600	-6.93169100
H	-1.87963500	0.30402200	-7.85371000
H	-2.77833700	-2.69341400	-4.91908700
H	-0.35686900	-3.09570500	-4.59351600
H	0.55652500	-0.07797800	-7.50992900
O	-3.77447300	-0.82774500	-6.62147200
H	-4.12111300	1.16774100	-5.04184600
H	1.32453400	-1.79020900	-5.87270100
O	-4.18292300	1.80722300	-5.85798300
O	-6.13822000	0.69607300	-6.73877500
H	-5.16043400	1.35589300	-6.37058300
H	-5.85706200	-0.26654900	-6.36672600
H	-3.44175400	1.50021800	-6.40867100
H	-6.14412800	0.60311500	-7.70367200

Structure and coordinates of **Int-o15**

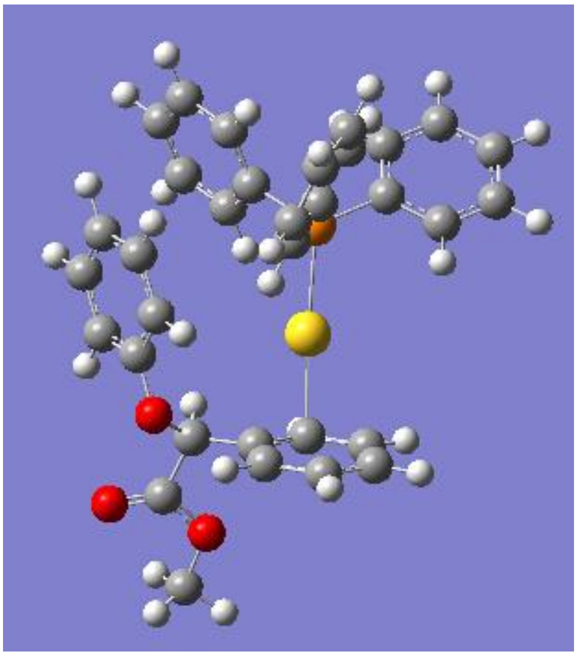

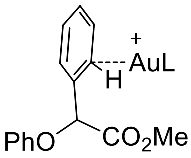

Int-o15
L = Ph_3P

C	1.22452800	1.15611700	3.80260300
C	1.13753300	2.51086000	3.39818800
C	1.69183500	2.91041200	2.15695600
C	2.28394100	1.94923400	1.33341900
C	2.36613600	0.62516200	1.75018000
C	1.83343200	0.22087300	2.97598200
H	0.83005300	0.86560000	4.77452000
H	0.84381700	3.27384400	4.12468400
H	2.68572500	2.26076000	0.37115500
H	2.85083600	-0.10724200	1.10753400
H	1.90337000	-0.81859000	3.28731300
C	1.66490000	4.36061200	1.73774300
C	2.90152000	5.16269700	2.15582300
O	3.27492800	6.14450800	1.57540300
O	3.43099800	4.66623600	3.27500800
C	4.54224100	5.40465300	3.80044600
H	4.86918000	4.85986300	4.68685000
H	5.34429400	5.45761200	3.05829400
H	4.23045300	6.42082000	4.06176200
C	-0.42118200	3.92290700	-0.90372500
C	0.28631000	4.82726700	-0.11834000
C	-0.21408800	6.09888000	0.14508200
C	-1.44357900	6.46424700	-0.39274400
C	-2.16077700	5.57014600	-1.18483700
C	-1.65027700	4.30044000	-1.43787900
H	0.02857300	2.95153100	-1.11131600
H	0.39212400	6.79676900	0.72266800
H	-1.83613800	7.46304500	-0.20840500
H	-2.21083100	3.59669500	-2.05336100
Au	-0.90988100	2.21254700	2.37443300
P	-3.17656500	1.96575300	1.62390600
C	-3.33560600	0.86040700	0.14677100
C	-4.53214100	0.18124300	-0.09117700
C	-2.26495400	0.73555900	-0.73842300
C	-4.65638500	-0.60904800	-1.22899100
H	-5.36104800	0.26456700	0.61245400
C	-2.39641100	-0.05526000	-1.87451000

H	-1.32796400	1.25516800	-0.53108900
C	-3.59251100	-0.72435300	-2.11953600
H	-5.58684400	-1.14051300	-1.41769800
H	-1.56227400	-0.15557300	-2.56585500
H	-3.69308100	-1.34640700	-3.00650300
C	-4.23219000	1.20221500	2.94597600
C	-5.44834900	1.77166000	3.31807700
C	-3.79547600	0.01226300	3.53259400
C	-6.22792000	1.14507200	4.28741200
H	-5.78860100	2.69913700	2.85816600
C	-4.58163100	-0.60877700	4.49419500
H	-2.84436700	-0.43053200	3.23062800
C	-5.79650300	-0.04027100	4.87250000
H	-7.17675300	1.58720500	4.58407600
H	-4.24596500	-1.53733700	4.95141200
H	-6.40880300	-0.52592000	5.62950600
C	-3.94678200	3.59983300	1.22159600
C	-4.91540600	3.70634700	0.22375400
C	-3.60302400	4.70773100	1.99554800
C	-5.54586000	4.92794000	0.00896200
H	-5.18055600	2.84086000	-0.38372400
C	-4.23727800	5.92496900	1.77610500
H	-2.84069600	4.61775600	2.77196700
C	-5.20908600	6.03382000	0.78477500
H	-6.30435800	5.01544200	-0.76646900
H	-3.96969200	6.79127200	2.37795400
H	-5.70611000	6.98718200	0.61581200
H	0.81843800	4.86258400	2.24454400
O	1.52540500	4.45617100	0.34833900
H	-3.11892300	5.86547500	-1.60814400

Structure and coordinates of **TS-c15-2w**

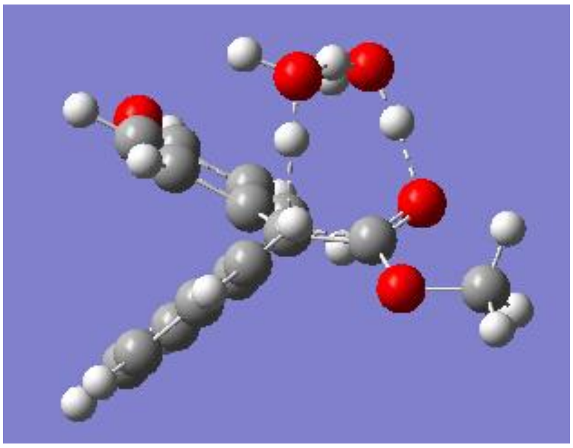

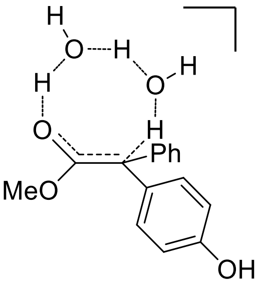

TS-c15-2w

C	5.57020800	-4.68418700	-8.67403400
C	5.00861000	-3.76270400	-7.79791300
C	5.68413400	-3.35059500	-6.64168600
C	6.94868900	-3.90716500	-6.40052200
C	7.51859000	-4.81753100	-7.28024400
C	6.83040400	-5.21625300	-8.42271300
H	5.01938600	-4.98175600	-9.56573100
H	4.02332500	-3.34676100	-8.00871000
H	7.49370800	-3.60844700	-5.50419600
H	8.50436000	-5.22896500	-7.06572000
H	7.27146700	-5.93711300	-9.10928000
C	5.06466400	-2.40009200	-5.67747400
C	5.74295000	-1.20121200	-5.31642000
O	5.36292100	-0.36081900	-4.45839900
O	6.93503100	-1.00516200	-5.91114300
C	7.70309400	0.08596300	-5.43154000
H	8.63611300	0.06211700	-5.99962000
H	7.18581200	1.03754600	-5.59671700
H	7.91081100	-0.01402800	-4.35977100
C	1.39733200	-1.41479300	-5.20307900
C	0.80029800	-2.65891800	-5.01518800
C	1.59022500	-3.80625600	-5.08160900
C	2.95143500	-3.69454800	-5.31503500
C	3.58970800	-2.45322200	-5.50454600
C	2.76404300	-1.31980500	-5.46045100
H	0.78751800	-0.50933800	-5.17491800
H	1.11632400	-4.77662100	-4.94507200
H	3.55187600	-4.60466600	-5.37763600
H	3.20442200	-0.33524400	-5.60682400
O	-0.52691500	-2.81741100	-4.76393900
H	4.53861400	-0.88372800	-3.33319500
O	4.04710300	-1.47197500	-2.61454900
O	5.38633300	-3.39472900	-3.16998800
H	4.63999500	-2.47510200	-2.75720600
H	5.39874700	-3.10706000	-4.20138100
H	3.16266300	-1.60482600	-3.00415100
H	4.94941200	-4.25849100	-3.13107900
H	-0.95922300	-1.95299600	-4.79704700

Structure and coordinates of **Int-c15**

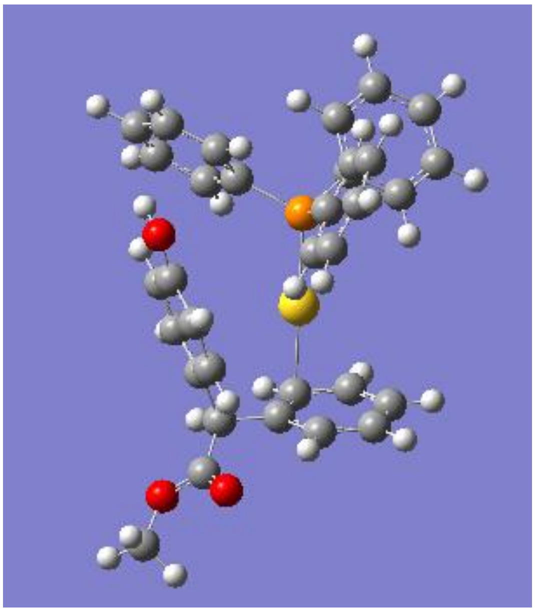

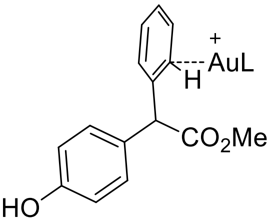

Int-c15

L = Ph_3P

C	0.78345500	1.47944600	4.47503900
C	1.04564000	2.70417800	3.81364300
C	1.82103700	2.72351700	2.62210000
C	2.28515600	1.51236600	2.11212800
C	2.01861600	0.31393500	2.77599000
C	1.26548800	0.28385800	3.94475300
H	0.24760400	1.49133900	5.42282700
H	0.83737100	3.64537100	4.33024000
H	2.89429300	1.51665800	1.21186600
H	2.41431900	-0.61465200	2.36871700
H	1.07474000	-0.65695000	4.45557700
C	2.06139500	4.06125400	1.94882800
C	3.47440400	4.21076700	1.40802300
O	4.03155600	3.41237000	0.68930400
O	4.00573000	5.36438600	1.80148500
C	5.31901900	5.63729900	1.29814400
H	5.59299200	6.61362400	1.69940300
H	6.02058900	4.86876200	1.63690800
H	5.30848100	5.65616400	0.20421500
C	1.16462600	3.70662900	-0.42003900
C	1.04435200	4.30132700	0.84038600
C	-0.07788800	5.08843300	1.10323900
C	-1.07189700	5.26373400	0.14657900
C	-0.95433300	4.64179700	-1.09621400
C	0.17971400	3.87699900	-1.38200900
H	2.04978700	3.11602300	-0.65430400
H	-0.17999100	5.58375600	2.07158200
H	-1.93821600	5.88933200	0.36572000
H	0.26596400	3.42261900	-2.36750700
Au	-0.99844500	2.42958600	2.75450500
P	-3.13343900	2.15724900	1.68369700
C	-2.97477400	1.10282000	0.17169200
C	-4.05876100	0.34802700	-0.28003500
C	-1.77543300	1.12191400	-0.54033800
C	-3.93613700	-0.38645200	-1.45439600
H	-4.99225200	0.33081100	0.28319900
C	-1.66463500	0.39170500	-1.71831000
H	-0.93066400	1.71335000	-0.17954000

C	-2.74296400	-0.36237500	-2.17252500
H	-4.77548900	-0.98108600	-1.80887700
H	-0.73166600	0.40790100	-2.27871200
H	-2.65352000	-0.93822900	-3.09143900
C	-4.37957700	1.33596200	2.78301100
C	-5.71360600	1.74210700	2.79398700
C	-3.96322900	0.24863200	3.55341600
C	-6.63107100	1.05654800	3.58529300
H	-6.03831600	2.58838400	2.18889100
C	-4.88639300	-0.43452000	4.33483200
H	-2.91768300	-0.06450700	3.53670700
C	-6.21877600	-0.02834300	4.35218800
H	-7.67225100	1.37195100	3.59952700
H	-4.56649400	-1.28390000	4.93488200
H	-6.93939100	-0.56151900	4.96881700
C	-3.88490700	3.76703100	1.14668100
C	-4.37737800	3.93366400	-0.14607000
C	-3.96582800	4.80905000	2.07351500
C	-4.94709400	5.15325500	-0.51220700
H	-4.30713900	3.12424300	-0.87239800
C	-4.53547400	6.02143800	1.70354200
H	-3.57706900	4.67230100	3.08445000
C	-5.02326800	6.19501300	0.40889500
H	-5.34752100	5.28125400	-1.51719300
H	-4.60033200	6.83285100	2.42550000
H	-5.47068900	7.14393400	0.12001300
H	1.92198100	4.84864600	2.70214500
O	-1.90133300	4.74043500	-2.05393400
H	-2.65246000	5.25062200	-1.71085100

Structure and coordinates of **Pro-o8**

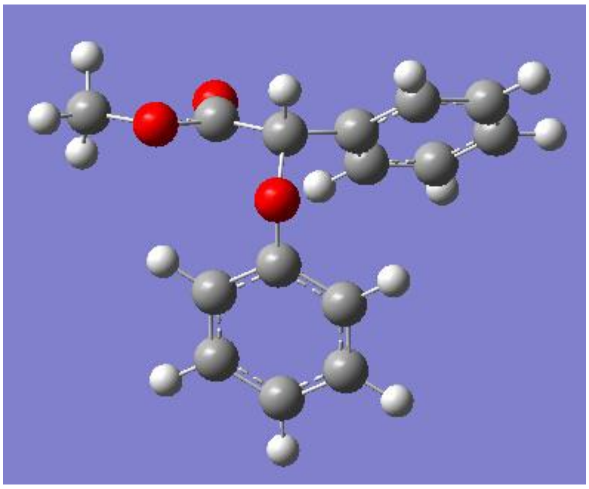

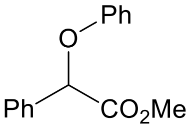

C	0.63122700	3.42877200	3.92219000
C	1.14539200	2.94894700	2.72356100
C	0.51033500	1.89799700	2.05726100
C	-0.63854900	1.33855900	2.61745000
C	-1.15352000	1.81704200	3.81636000
C	-0.51692000	2.86594100	4.47083800
H	1.13390700	4.24879600	4.43206500
H	2.04447700	3.39349400	2.30266500
H	-1.13119300	0.50979500	2.10700700
H	-2.05355200	1.37254800	4.23776800
H	-0.91616800	3.24671500	5.40964700
C	1.08005600	1.24809100	0.82657700
C	1.65388000	2.21926900	-0.20539300
O	1.63483900	3.42314400	-0.14174000
O	2.15453900	1.53958000	-1.24767700
C	2.73086100	2.35166000	-2.26575800
H	3.09222700	1.66227200	-3.03095000
H	1.98654900	3.03784600	-2.68374300
H	3.55986200	2.94313300	-1.85896300
C	5.37133900	1.43706500	1.95159900
C	5.37854100	1.27334200	3.33291700
C	4.25586100	0.75707700	3.97426900
C	3.12574800	0.41435600	3.24164500
C	3.12393100	0.58652300	1.86010900
C	4.24655600	1.09233100	1.20893700
H	6.25122200	1.82934000	1.44415800
H	4.25708300	0.62442000	5.05480800
H	2.23026500	0.02186900	3.72081900
H	4.23184300	1.18893900	0.12484100
O	2.01249000	0.21033700	1.13845800
H	0.27558400	0.70825400	0.30432900
H	6.26133300	1.54272400	3.90988300

Structure and coordinates of **Pro-c8**

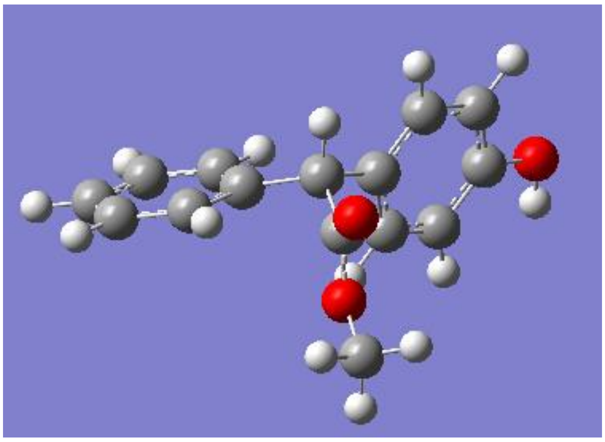

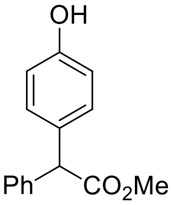

C	-0.14328100	3.86915800	2.74796600
C	0.25296600	3.19791000	1.59857800
C	0.82235200	1.92389100	1.67345700
C	0.97127500	1.33189800	2.92645100
C	0.57228200	2.00209300	4.07881200
C	0.01731000	3.27327900	3.99519200
H	-0.58181400	4.86256000	2.66740000
H	0.10868300	3.66843500	0.62438300
H	1.40459800	0.33584700	3.00198500
H	0.69772600	1.52385700	5.04911800
H	-0.29092000	3.79869900	4.89746300
C	1.18463800	1.18192400	0.39334300
C	1.66148200	2.15810600	-0.67044500
O	1.06195200	2.41145200	-1.68709900
O	2.82407500	2.73928900	-0.33480900
C	3.30808200	3.69307700	-1.27395200
H	4.24957000	4.06732000	-0.86733800
H	3.46911700	3.22585000	-2.25134000
H	2.58909600	4.51140400	-1.39257600
C	1.79885100	-1.24380700	0.11684800
C	2.15389000	0.03151200	0.55927900
C	3.41542000	0.20007900	1.13520000
C	4.29043300	-0.86926600	1.26369800
C	3.91716300	-2.13564500	0.81274800
C	2.66394000	-2.32203000	0.23599700
H	0.81884300	-1.39678100	-0.33608000
H	3.71496500	1.18558600	1.48831200
H	5.27211900	-0.72196800	1.71760500
H	2.38717800	-3.31559900	-0.10950700
O	4.73141200	-3.21727000	0.91203400
H	5.56522900	-2.94871900	1.32201700
H	0.25381600	0.77496200	-0.03048300

Section 12: ^{1}H-NMR spectrum.

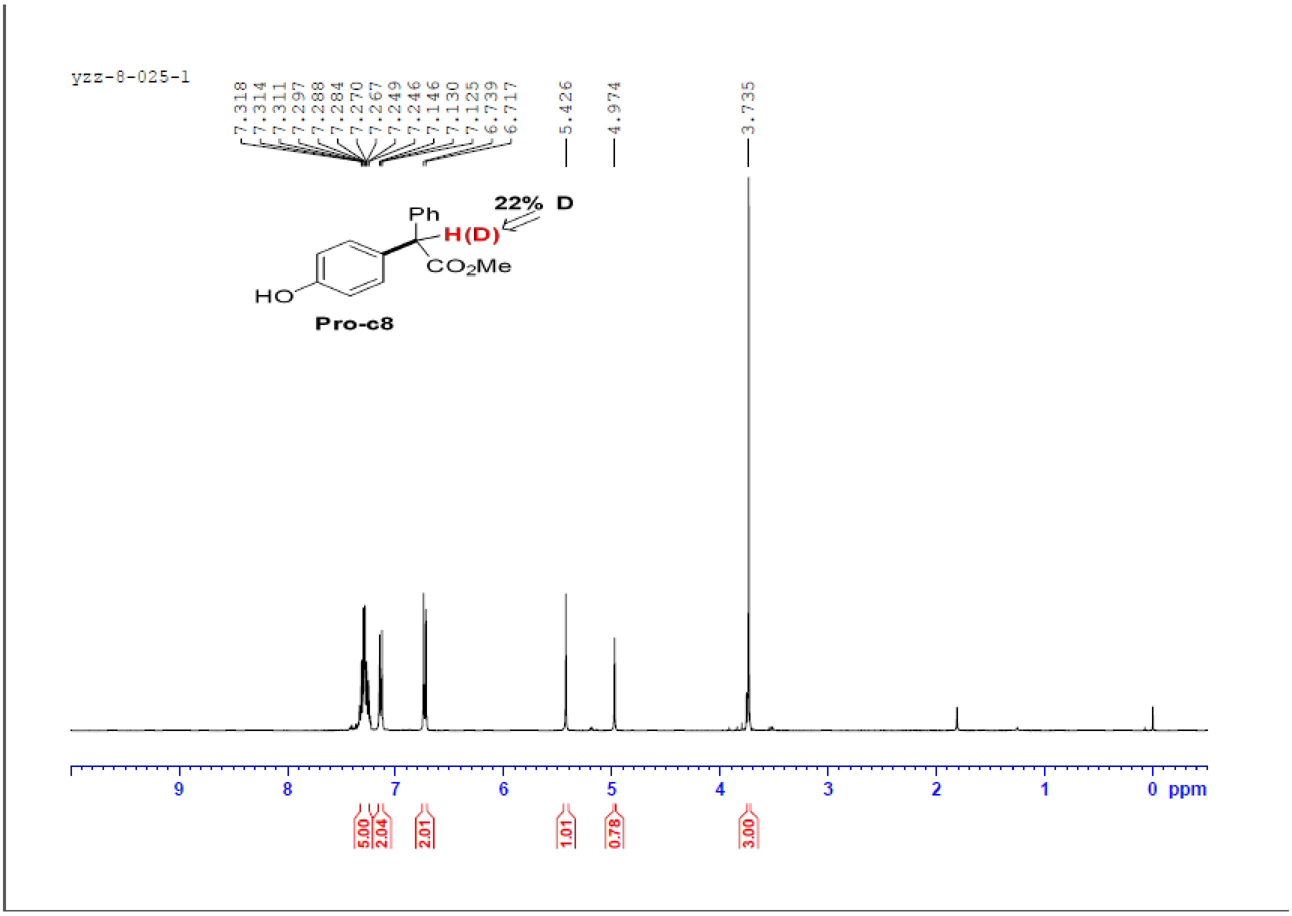

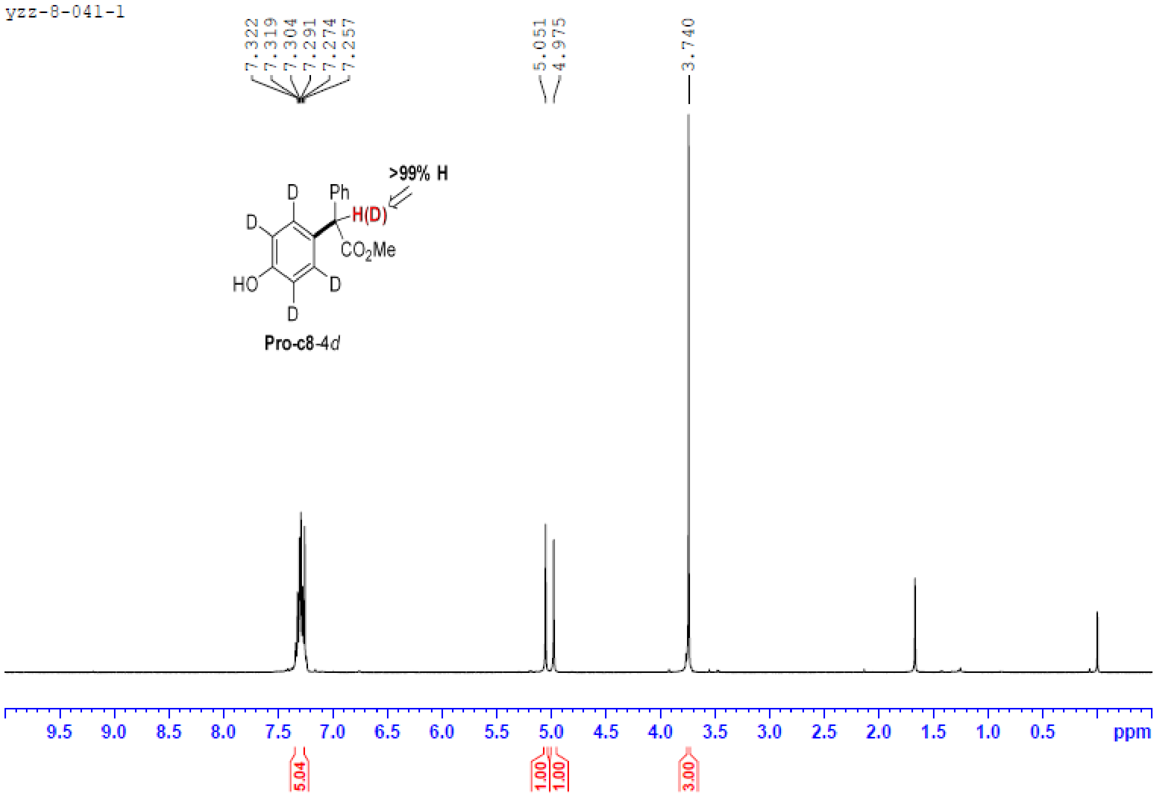

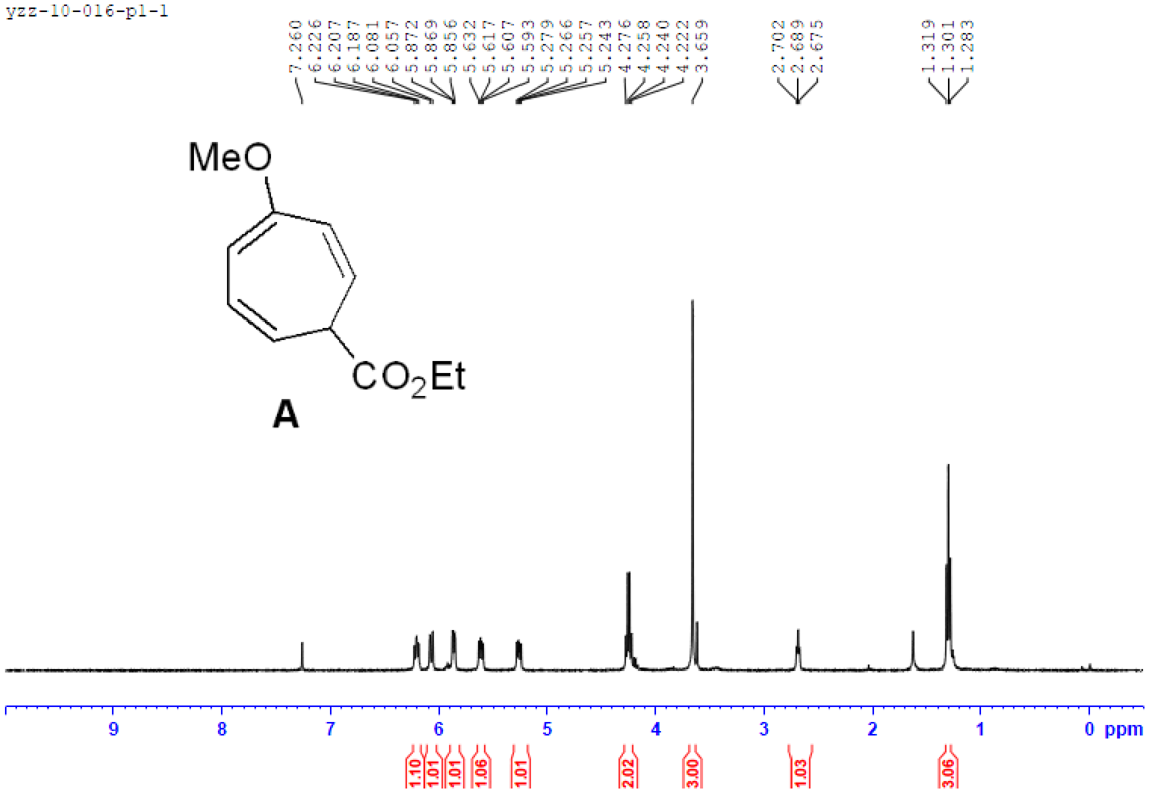
yzz-10-016-p1-1
7.260
6.226
6.207
6.187
6.081
6.057
5.872
5.869
5.856
5.632
5.617
5.607
5.593
5.279
5.266
5.257
5.243
4.276
4.258
4.240
4.222
3.659
2.702
2.689
2.675
1.319
1.301
1.283
MeO
CO2Et
A
9 8 7 6 5 4 3 2 1 0 ppm
1.10 1.01 1.01 1.06 1.01 2.02 3.00 1.03 3.06

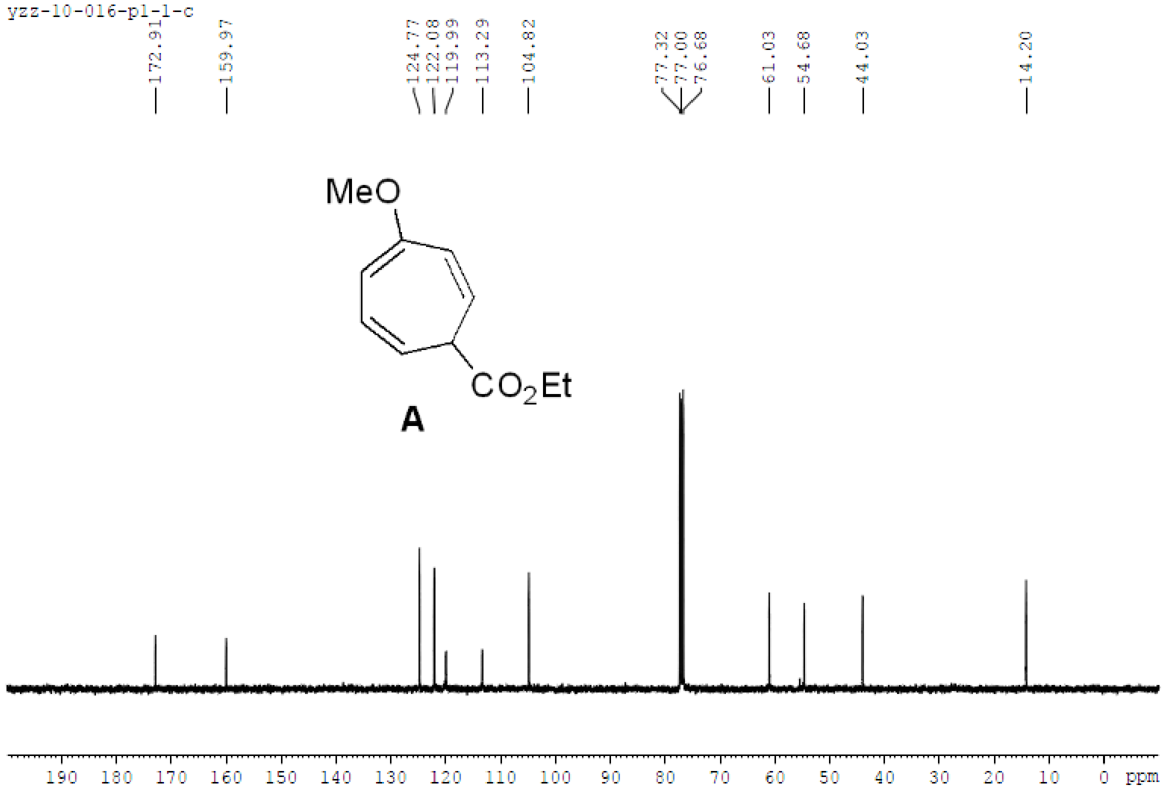
yzz-10-016-p1-1-c
172.91
159.97
124.77
122.08
119.99
113.29
104.82
77.32
77.00
76.68
61.03
54.68
44.03
14.20
MeO
CO2Et
A
190 180 170 160 150 140 130 120 110 100 90 80 70 60 50 40 30 20 10 0 ppm
